# Supplementary material for: Status of zoonotic disease research in refugees, asylum seekers and internally displaced people, globally: A scoping review of forty clinically important zoonotic pathogens
Source: PLoS Negl Trop Dis. 2024 May 20;18(5):e0012164. doi: 10.1371/journal.pntd.0012164 (PMC11142688; doi:10.1371/journal.pntd.0012164)
Supplement: S2 Text — (DOCX) [file pntd.0012164.s002.docx]

**S2 Text: Pubmed search string.** ‘Displaced people’, ‘zoonoses’ and ‘disease specific name’ search strings separated by Boolean operators in bold.

(“refugees”[MeSH] OR “refugee*”[tiab] OR “refugee camps”[MeSH] OR “asylum seeker*”[tiab] OR “migrant*”[tiab] OR “displaced person*”[tiab] OR “IDP”[tiab] OR ”IDPs”[tiab] OR “displaced people”[tiab] OR “displaced women”[tiab] OR “displaced men”[tiab] OR “displaced woman”[tiab] OR “displaced man”[tiab] OR “displaced child*”[tiab] OR “displaced minor*”[tiab]) **AND** ((“zoonoses”[MeSH] OR "Ectoparasitic Infestations"[Mesh:NoExp] OR “flea infestations”[MESH] OR “lice infestations”[MESH] OR “mite infestations”[MESH] OR “tick infestations”[MESH] OR "Neglected Diseases"[Mesh] OR “zoono*”[tiab] OR “neglected tropical disease”[tiab] OR “ectoparasit*”[tiab] OR “tick*”[tiab] OR “flea*”[tiab] OR “mite*”[tiab] OR “lice”[tiab] OR “louse”[tiab] OR “pediculos*”[tiab] OR “Acariasis”[tiab] OR “Mange”[tiab]) **OR** (“brucella”[MesH] OR “brucell*”[tiab] OR “brucellosis”[MeSH] OR “brucella vaccine”[MeSH] OR “malta fever”[tiab] OR “fever, malta”[tiab] OR “Gibraltar fever”[tiab] OR “fever, Gibraltar”[tiab] OR “rock fever”[tiab] OR “fever, rock”[tiab] OR “cyprus fever”[tiab] OR “fever, cyprus”[tiab] OR “undulant fever”[tiab] OR “fever, undulant”[tiab] OR “rickettsia”[MeSH] OR “rickettsia infections”[MeSH] OR “rickettsial vaccines”[MeSH] OR “rickettsi*”[tiab] OR “typhus”[tiab] OR “spotted fever”[tiab] OR “Tick-Borne Lymphadenopath*”[tiab] OR “Tick Borne Lymphadenopath*”[tiab] OR “TIBOLA”[tiab] OR “tick-bite fever”[tiab] OR “tick bite fever”[tiab] OR “jail fever”[tiab] OR “fever, jail”[tiab] OR “Brill-Zinsser Disease”[tiab] OR “Brill Zinsser Disease”[tiab] OR “Brill’s Disease”[tiab] OR “Brills Disease”[tiab] OR “Brill Disease”[tiab] OR “leptospira”[MesH] OR “leptospirosis”[MesH] OR “leptospir*”[tiab] OR “stuttgard disease”[tiab] OR “mud fever”[tiab] OR “fever, mud”[tiab] OR “rice-field fever”[tiab] OR “rice field fever”[tiab] OR “fever, rice-field”[tiab] OR “cane-cutter fever”[tiab] OR “cane cutter fever”[tiab] OR “fevers, cane-cutter”[tiab] OR “swineherd’s disease*”[tiab] OR “canicola*”[tiab] OR “weil disease”[tiab] OR “weil’s disease”[tiab] OR “weil’s disease”[tiab] OR “disease, weil”[tiab] OR “disease, weil’s”[tiab] OR “spirochetal jaundice”[tiab] OR “jaundice, spirochetal”[tiab] OR “boutonneuse fever”[tiab] OR “Marseilles fever”[tiab] OR “Orientia tsutsugamushi”[MeSH] OR “Scrub typhus”[MeSH] OR “Orientia”[tiab] OR “tsutsugamushi”[tiab] OR “Scrub typhus”[tiab] OR “Q fever”[MesH] OR “Q fever”[tiab] OR “query fever”[tiab] OR “Coxiella burnetii”[MesH] OR “Coxiella burnetii”[tiab] OR “coxiellosis”[tiab] OR “Relapsing fever”[MesH] OR “Borrelia recurrentis”[supplementary concept] OR “Relapsing fever*”[tiab] OR “Borrelia recurrentis”[tiab] OR “Louse-borne relapsing fever”[tiab] OR “LBRF”[tiab] OR “protomycetum recurrentis”[tiab] OR “spiroschaudinnia recurrentis”[tiab] OR “salmonella typhi”[MesH] OR “salmonell*”[tiab] OR “typhoid fever”[MesH] OR “typhoid-paratyphoid vaccines”[MeSH] OR “typhoid fever”[tiab] OR “Bacillus typhi”[tiab] OR “Bacillus typhosus”[tiab] OR “Bacterium typhi”[tiab] OR “Bacterium typhosum”[tiab] OR “Eberth bacillus”[tiab] OR “Eberthella typhi”[tiab] OR “Eberthella typhosa”[tiab] OR “typhoid bacillus”[tiab] OR “typhus bacillus”[tiab] OR “enteric fever”[tiab] OR “plague”[MesH] OR “plague vaccines”[MeSH] OR “plague”[tiab] OR “Yersinia pestis”[MesH] OR “pestis”[tiab] OR “rift valley fever”[MeSH] OR “rift valley fever virus”[MeSH] OR “rift valley fever”[tiab] OR “RVF”[tiab] OR “rift valley virus”[tiab] OR “RVFV”[tiab] OR “Middle east respiratory syndrome coronavirus”[MeSH] OR “Middle east respiratory syndrome”[tiab] OR “MERS*”[tiab] OR “rabies”[MeSH] OR “rabies vaccines”[MeSH] OR “rabies virus”[MeSH] OR “rabies”[tiab] OR “lyssa*”[tiab] OR “hubert disease”[tiab] OR “rabbia”[tiab] OR “hydrophobia agent”[tiab] OR “hydrophobia virus”[tiab] OR “ebolavirus”[MeSH] OR “hemorrhagic fever, ebola”[MeSH] OR “ebola vaccines”[MeSH] OR “ebola*”[tiab] OR “hemorrhagic fever virus, crimean-congo”[MeSH] OR “hemorrhagic fever, Crimean”[MeSH] OR “Crimean-Congo Hemorrhagic”[tiab] OR “Crimean Congo Hemorrhagic”[tiab] OR “Crimean-Congo Haemorrhagic”[tiab] OR “Crimean Congo Haemorrhagic”[tiab] OR “congo virus”[tiab] OR “crimean hemorrhagic”[tiab] OR “Crimean haemorrahagic”[tiab] OR “CCHF”[tiab] OR “Flavivirus”[MeSH] OR “Flavivirus infections”[MeSH] OR “Flavivirus*”[tiab] OR “Flavi virus*”[tiab] OR “Dengue vaccines”[MeSH] OR “Dengue”[tiab] OR “dandy fever”[tiab] OR “denque haemorrhagic virus”[tiab] OR “denque hemorrhagic virus”[tiab] OR “DENV”[tiab] OR “breakbone”[tiab] OR “Zika”[tiab] OR “zikV”[tiab] OR “Japanese Encephalitis”[tiab] OR “Japanese encephalitis vaccines”[MeSH] OR “Murry river encephalitis”[tiab] OR “St Louis encephalitis”[tiab] OR “Usutu”[tiab] OR “Meningoencephaliti*”[tiab] OR “Lethargic Encephalitis”[tiab] OR “Tick-borne encephalitis”[tiab] OR “Tick borne encephalitis”[tiab] OR “Louping ill”[tiab] OR “Powassan”[tiab] OR “central European encephalitis”[tiab] OR “Russian encephalitis”[tiab] OR “Russian sping summer encephalitis”[tiab] or “Russian sping-summer encephalitis”[tiab] OR “Omsk hemorrhagic”[tiab] OR “Omsk haemorrhagic”[tiab] OR “Kyasanur Forest disease”[tiab] OR “Alkhurma virus”[tiab] OR “Al-khurma virus”[tiab] OR “Al khurma virus”[tiab] OR “Al Khurma Hemorrhagic Fever”[tiab] OR “Al-Khurma Hemorrhagic Fever”[tiab] OR “AlKhurma Hemorrhagic Fever”[tiab] OR “Al Khurma Haemorrhagic Fever”[tiab] OR “Al-Khurma Haemorrhagic Fever”[tiab] OR “AlKhurma Haemorrhagic Fever”[tiab] OR “Yellow fever*”[tiab] OR “yellow fever vaccine”[MeSH] OR “west nile fever”[tiab] OR “west nile virus vaccines”[MeSH] OR “WNF”[tiab] OR “West Nile virus”[tiab] OR “WNFV”[tiab] OR “Egypt 101 virus”[tiab] OR “Chikungunya virus”[MeSH] OR “Chikungunya fever”[MeSH] OR “Chikungunya”[tiab] OR “Chickungunya”[tiab] OR “Marburgvirus”[MeSH] OR “Marburg Virus Disease”[MeSH] OR “Marburg*”[tiab] OR “lassa fever”[MeSH] OR “lassa virus”[MeSH] OR “lassa”[tiab] OR “nipah virus”[MeSH] OR “henipavirus infections”[MeSH] OR “nipah”[tiab] OR “niV”[tiab] OR “henipavirus*”[tiab] OR “hepatitis E virus”[MeSH] OR “hepatitis E”[MeSH] OR “hepatitis E”[tiab] OR “HEV”[tiab] OR “water-borne hepatiti*”[tiab] OR “water borne hepatitis*”[tiab] OR “Hepatitis, Water-Borne”[tiab] OR “Hepatitis, Water Borne”[tiab] OR “Hepatitides, Water-Borne”[tiab] OR “ET-NANBH”[tiab] OR “Hepatitis, Viral, Non-A, Non-B, Enterically-Transmitted”[tiab] OR “Enterically-Transmitted Non-A, Non-B Hepatitis”[tiab] OR “Enterically Transmitted Non A, Non B Hepatitis”[tiab] OR “Epidemic Non-A, Non-B Hepatitis”[tiab] OR “Epidemic Non A, Non B Hepatitis”[tiab] OR “hantavirus”[MeSH] OR “hantavirus infections”[MeSH] OR “hantavirus*”[tiab] OR “HARDS”[tiab] OR “andes virus*”[tiab] OR “andes hantavirus*”[tiab] OR “Hantavirus, Andes”[tiab] OR “Hantaviruses, Andes”[tiab] OR “orthohantavirus*”[tiab] OR “Dobrava-Belgrade Virus*”[tiab] OR “Dobrava Belgrade Virus*”[tiab] OR “Cysticercosis”[MeSH] OR “Taenia solium”[MeSH] OR “Cysticercos*”[tiab] OR “solium*”[tiab] OR “tapeworm, pork”[tiab] OR “pork tapeworm*”[tiab] OR “Neurocysticercos*”[tiab] OR “Neurocoenuros*”[tiab] OR “Coenur*”[tiab] OR “Taenia*”[tiab] OR “Echinococcosis”[MeSH] OR “Echinococcus”[MeSH] OR “Echinococc*”[tiab] OR “hydati*”[tiab] OR “toxoplasmosis”[MeSH] OR “toxoplasma”[MeSH] OR “toxoplasm*”[tiab] OR “filariasis”[MeSH] OR “Filarioidea”[MeSH] OR “filarioidea*”[tiab] OR “filaria*”[tiab] OR “Litomosoide*”[tiab] OR “elaeophoriasis”[tiab] OR “Acanthocheilonema*”[tiab] OR “Brugia*”[tiab] OR “Dipetalonema*”[tiab] OR “Dirofilaria*”[tiab] OR “dog heartworm*”[tiab] OR “loa”[tiab] OR “loas”[tiab] OR “Mansonella*”[tiab] OR “Tetrapetalonema*”[tiab] OR “Microfilaria*”[tiab] OR “Onchocerca*”[tiab] OR “river blindness”[tiab] OR “Setaria*”[tiab] OR “Wuchereria*”[tiab] OR “schistosoma”[MeSH] OR “schistosomiasis”[MeSH] OR “schistosom*”[tiab] OR “Bilharzi*”[tiab] OR “Katayama Fever”[tiab] OR “fever, Katayama”[tiab] OR “Fasciola”[MeSH] OR “Fascioliasis”[MeSH] OR “Fasciol*”[tiab] OR “hepatic distomiasis”[tiab] OR “liver fluke disease”[tiab] OR “liver fluke infection*”[tiab] OR “Trichinella”[MeSH] OR “Trichinellosis”[MeSH] OR “Trichin*”[tiab] OR “trichenosis”[tiab] OR “Cryptosporidiosis”[MeSH] OR “Cryptosporidium”[MeSH] OR “Cryptospori*”[tiab] OR “Leishmania”[MeSH] OR “leishmaniasis vaccines”[MeSH] OR “Leishmaniasis”[MeSH] OR “Leishman*”[tiab] OR “Giardia”[MeSH] OR “Giardiasis”[MeSH] OR “Giardi*”[tiab] OR “lamblias*”[tiab] OR “trypanosoma”[MeSH] OR “trypanosomiasis”[MeSH] OR “trypanosom*”[tiab] OR “Nannomona*”[tiab] OR “Chagas”[tiab] OR “sleeping sickness*”[tiab] OR “HAT”[tiab] OR “nagana”[tiab] OR “Sickness, African Sleeping”[tiab] OR “Sicknesses, African Sleeping”[tiab] OR “Anaplasma”[MeSH] OR “Anaplasmosis”[MeSH] OR “Anaplasm*”[tiab] OR “Tularemia”[MeSH] OR “Francisella tularensis”[MeSH] OR “Tularemia*”[tiab] OR “tularens*”[tiab] OR “ohara disease”[tiab] OR “tularaemia”[tiab] OR “yato bya”[tiab] OR “SARS virus”[MeSH] OR “Severe Acute Respiratory Syndrome”[MeSH] OR “SARS*”[tiab] OR “Severe Acute Respiratory Syndrome*”[tiab] OR “Respiratory Syndrome, Severe Acute”[tiab] OR “Respiratory Syndrome, Acute, Severe”[tiab]) **OR** (((Coronavirus[mh:noexp] OR Betacoronavirus[mh:noexp] OR Coronavirus Infections[mh:noexp]) AND (Disease Outbreaks[mh:noexp] OR Epidemics[mh:noexp] OR Pandemics[mh])) OR COVID-19 testing [MeSH] OR COVID-19 drug treatment [Supplementary Concept] OR COVID-19 serotherapy [Supplementary Concept] OR COVID-19 vaccines [MeSH] OR spike glycoprotein, COVID-19 virus [Supplementary Concept] OR COVID-19 [MeSH] OR SARS-CoV-2 [MeSH] OR nCoV[tiab] OR nCoV[tt] OR 2019nCoV[tiab] OR 2019nCoV[tt] OR 19nCoV[tiab] OR 19nCoV[tt] OR COVID19*[tiab] OR COVID19*[tt] OR COVID[tiab] OR COVID[tt] OR SARS-CoV-2[tiab] OR SARS-CoV-2[tt] OR SARSCOV-2[tiab] OR SARSCOV-2[tt] OR SARSCOV2[tiab] OR SARSCOV2[tt] OR Severe Acute Respiratory Syndrome Coronavirus 2[tiab] OR Severe Acute Respiratory Syndrome Coronavirus 2[tt] OR ((severe acute respiratory syndrome[tiab] OR severe acute respiratory syndrome[tt]) AND (corona virus 2[tiab] OR corona virus 2[tt])) OR new coronavirus[tiab] OR (new[tt] AND coronavirus[tt]) OR novel coronavirus[tiab] OR novel coronavirus[tt] OR novel corona virus[tiab] OR (novel[tt] AND corona virus[tt]) OR novel CoV[tiab] OR (novel[tt] AND CoV[tt]) OR novel HCoV[tiab] OR (novel[tt] AND HCoV[tt]) OR ((“19″[tiab] OR “19”[tt] OR “2019”[tiab] OR “2019”[tt] OR Wuhan[tiab] OR Wuhan[tt] OR Hubei[tiab] OR Hubei[tt]) AND (coronavirus*[tiab] OR coronavirus*[tt] OR corona virus*[tiab] OR corona virus*[tt] OR CoV[tiab] OR CoV[tt] OR HCoV[tiab] OR HCoV[tt])) OR ((coronavirus*[tiab] OR coronavirus*[tt] OR corona virus*[tiab] OR corona virus*[tt] OR betacoronavirus*[tiab] OR betacoronavirus*[tt]) AND (outbreak*[tiab] OR outbreak*[tt] OR epidemic*[tiab] OR epidemic*[tt] OR pandemic*[tiab] OR pandemic*[tt] OR crisis[tiab] OR crisis[tt])) OR ((Wuhan[tiab] OR Wuhan[tt] OR Hubei[tiab] OR Hubei[tt]) AND (pneumonia[tiab] OR pneumonia[tt])) AND 2019/10/31:3000/12/31[Date – Publication]))

**Filters applied:** *Case Reports, Classical Article, Clinical Study, Clinical Trial, Clinical Trial, Phase I, Clinical Trial, Phase II, Clinical Trial, Phase III, Clinical Trial, Phase IV, Clinical Trial, Veterinary, Comparative Study, Controlled Clinical Trial, Corrected and Republished Article, Evaluation Study, Historical Article, Introductory Journal Article, Meta-Analysis, Multicenter Study, Observational Study, Pragmatic Clinical Trial, Randomized Controlled Trial, Review, Systematic Review, Validation Study, Observational Study, Veterinary*.
