# Supplementary material for: Status of zoonotic disease research in refugees, asylum seekers and internally displaced people, globally: A scoping review of forty clinically important zoonotic pathogens
Source: PLoS Negl Trop Dis. 2024 May 20;18(5):e0012164. doi: 10.1371/journal.pntd.0012164 (PMC11142688; doi:10.1371/journal.pntd.0012164)
Supplement: S1 Table — (DOCX) [file pntd.0012164.s003.docx]

**S1 Table: Publications included in the scoping review**

| Pathogen | References |
| --- | --- |
| Bacteria |  |
| *Brucella* spp. | [1-4] |
| *Rickettsia* spp. | [5-12] |
| *Leptospira* spp. | [10, 13-16] |
| *Coxiella burnetii* | [9, 17] |
| *Borrelia recurrentis* | [6, 13, 18-38] |
| *Orientia tsutsugamushi* | [10] |
| *Salmonella enterica* subsp. *enterica* serovar typhi | [1, 6, 18, 27, 39-52] |
| *Yersinia pestis* | Not applicable |
| *Anaplasma* spp. | Not applicable |
| *Francisella tularensis* | Not applicable |
| Parasites |  |
| *Schistosoma* spp. | [6, 36, 44, 50, 53-147] |
| *Giardia lamblia* | [1, 6, 19, 40, 44, 45, 47, 50, 57, 62, 66, 67, 70-73, 77, 80, 82, 83, 85, 88, 89, 94, 97, 104, 105, 109-114, 116, 121, 125-127, 132, 137, 138, 142, 145, 146, 148-184] |
| *Leishmania* spp. | [1, 6, 27, 36, 52, 89, 122, 185-236] |
| *Taenia solium* | [66, 94, 97, 127, 137, 156, 237-246] |
| *Echinococcus* spp. | [1, 30, 122, 134, 247-257] |
| *Toxoplasma gondii* | [1, 71, 122, 138, 258-265] |
| *Filarioidea* | [59, 105, 127, 146, 266-272] |
| *Fasciola* spp. | [6, 156, 157] |
| *Trichinella* spp. | [273, 274] |
| *Cryptosporidia* spp. | [40, 45, 47, 66, 72, 113, 138, 153, 168, 179, 275] |
| *Trypanosoma* spp. | [27, 276-278] |
| Viruses |  |
| Hepatitis E virus | [16, 27, 40, 134, 279-301] |
| Rift valley fever virus | [6, 27, 302] |
| Ebola virus | [27] |
| MERS-CoV | [1] |
| Rabies virus | [303] |
| SARS-CoV | Not applicable |
| SARS-CoV-2 | [304-339] |
| Chikungunya virus | [302, 340] |
| Marburg virus | [27] |
| Lassa virus | [27] |
| Nipah virus | Not applicable |
| Hantavirus | Not applicable |
| Crimean-Congo haemorrhagic fever virus | [1, 72, 302, 341-343] |
| Dengue virus | [10, 27, 302, 340, 341, 343-345] |
| West Nile virus | [302, 341] |
| Yellow fever virus | [27, 302, 346] |
| Zika virus | [302, 340] |
| Japanese encephalitis virus | [347] |
| Tick-borne encephalitis virus | Not applicable |

**Reference**

1. Aliskin O, Savas N. Notifiable communicable diseases in Turkey and their notification status: Antakya sample. Flora the Journal of Infectious Diseases and Clinical Microbiology. 2019;24(1):11-21.

2. Grunow R, Jacob D, Klee S, Schlembach D, Jackowski-Dohrmann S, Loenning-Baucke V, et al. Brucellosis in a refugee who migrated from Syria to Germany and lessons learnt, 2016. Eurosurveillance. 2016;21(31):5-8.

3. Jensenius M, Hoiby EA, Berild D, Stiris M, Ringertz SH. Difficulties in diagnosing Brucella spondylitis. Scand J Infect Dis. 2000;32(4):425-6.

4. Enkelmann J, Stark K, Faber M. Epidemiological trends of notified human brucellosis in Germany, 2006–2018. Int J Infect Dis. 2020;93:353-8.

5. Badiaga S, Brouqui P, Raoult D. Autochthonous epidemic typhus associated with Bartonella quintana bacteremia in a homeless person. Am J Trop Med Hyg. 2005;72(5):638-9.

6. Berger SA, Schwartz T, Michaeli D. Infectious disease among Ethiopian immigrants in Israel. Arch Intern Med. 1989;149(1):117-9.

7. Brown AE, Meek SR, Maneechai N, Lewis GE. Murine typhus among Khmers living at an evacuation site on the Thai-Kampuchean border. Am J Trop Med Hyg. 1988;38(1):168-71.

8. Duffy PE, Le Guillouzic H, Gass RF, Innis BL. Murine typhus identified as a major cause of febrile illness in a camp for displaced Khmers in Thailand. Am J Trop Med Hyg. 1990;43(5):520-6.

9. Gray GC, Rodier GR, Matras-Maslin VC, Honein MA, Ismail EA, Botros BA, et al. Serologic evidence of respiratory and rickettsial infections among Somali refugees. Am J Trop Med Hyg. 1995;52(4):349-53. doi: 10.4269/ajtmh.1995.52.349.

10. McGready R, Ashley EA, Wuthiekanun V, Tan SO, Pimanpanarak M, Viladpai-Nguen SJ, et al. Arthropod borne disease: The leading cause of fever in pregnancy on the thai-burmese border. PLoS Negl Trop Dis. 2010;4(11).

11. Raoult D, Ndihokubwayo JB, Tissot-Dupont H, Roux V, Faugere B, Abegbinni R, et al. Outbreak of epidemic typhus associated with trench fever in Burundi. The Lancet. 1998;352(9125):353-8.

12. Um J, Nam Y, Lim JN, Kim M, An Y, Hwang SH, et al. Seroprevalence of scrub typhus, murine typhus and spotted fever groups in North Korean refugees. International Journal of Infectious Diseases. 2021;106:23-8.

13. Cutuli SL, De Pascale G, Spanu T, Dell'Anna AM, Bocci MG, Pallavicini F, et al. Lice, rodents, and many hopes: a rare disease in a young refugee. Crit Care. 2017;21:3.

14. Lin CY, Chen TC, Dai CY, Yu ML, Lu PL, Yen JH, et al. Serological investigation to identify risk factors for post-flood infectious diseases: a longitudinal survey among people displaced by Typhoon Morakot in Taiwan. BMJ Open. 2015;5(5):e007008.

15. Hanapi IRM, Sahimin N, Maackara MJB, Annisa AS, Mutalib R, Lewis JW, et al. Prevalence of anti-Leptospira antibodies and associated risk factors in the Malaysian refugee communities. BMC Infect Dis. 2021;21(1). doi:10.1186/s12879-021-06830-0.

16. Mazhar MKA, Finger F, Evers ES, Kuehne A, Ivey M, Yesurajan F, et al. An outbreak of acute jaundice syndrome (AJS) among the Rohingya refugees in Cox’s Bazar, Bangladesh: Findings from enhanced epidemiological surveillance. PLoS One. 2021;16(4). doi:10.1371/journal.pone.0250505.

17. Qazi M, Weimer AC, Bedard BA, Kennedy BS. Q-fever in a refugee after exposure to a central New York State livestock farm. Annals of Tropical Medicine and Public Health. 2016;9(4):266-70.

18. Ackermann N, Marosevic D, Hörmansdorfer S, Eberle U, Rieder G, Treis B, et al. Screening for infectious diseases among newly arrived asylum seekers, Bavaria, Germany, 2015. Eurosurveillance. 2018;23(10).

19. Alberer M, Malinowski S, Sanftenberg L, Schelling J. Notifiable infectious diseases in refugees and asylum seekers: experience from a major reception center in Munich, Germany. Infection. 2018;46(3):375-83.

20. Antinori S, Mediannikov O, Corbellino M, Gr, e R, Parravicini C, et al. Louse-Borne Relapsing Fever (Borrelia recurrentis) in a Somali Refugee Arriving in Italy: A Re-emerging Infection in Europe? PLoS Negl Trop Dis. 2016;10(5).

21. Antinori S, Mediannikov O, Corbellino M, Raoult D. Louse-borne relapsing fever among East African refugees in Europe. Travel Med Infect Dis. 2016;14(2):110-4.

22. Antinori S, Tonello C, Edouard S, Parravicini C, Gastaldi D, Gr, et al. Diagnosis of louse-borne relapsing fever despite negative microscopy in two asylum seekers from Eastern Africa. Am J Trop Med Hyg. 2017;97(6):1669-72.

23. Bloch-Infanger C, Bättig V, Kremo J, Widmer AF, Egli A, Bingisser R, et al. Increasing prevalence of infectious diseases in asylum seekers at a tertiary care hospital in Switzerland. PLoS One. 2017;12(6):e0179537.

24. Brown V, Larouze B, Desve G, Rousset JJ, Thibon M, Fourrier A, et al. Clinical presentation of louse-born relapsing fever among Ethiopian refugees in northern Somalia. Ann Trop Med Parasitol. 1988;82(5):499-502.

25. Costescu Strachinaru DI, Cambier J, et-Yattara H, Konopnicki D. Relapsing fever in asylum seekers from Somalia arriving in Belgium in August 2015. Acta Clin Belg. 2016;71(5):353-5.

26. Darcis G, Hayette MP, Bontems S, Sauvage AS, Meuris C, Van Esbroeck M, et al. Louse-borne relapsing fever in a refugee from Somalia arriving in Belgium. J Travel Med. 2016;23(3):3.

27. Desai AN, Ramatowski JW, Marano N, Madoff LC, Lassmann B. Infectious disease outbreaks among forcibly displaced persons: An analysis of ProMED reports 1996-2016. Conflict and Health. 2020;14(1).

28. Goldenberger D, Claas GJ, Bloch-Infanger C, Breidthardt T, Suter B, Martinez M, et al. Louse-borne relapsing fever (Borrelia recurrentis) in an Eritrean refugee arriving in Switzerland, August 2015. Euro Surveill. 2015;20(32):2-5.

29. Grecchi C, Zanotti P, Pontarelli A, Chiari E, Tomasoni LR, Gulletta M, et al. Louse-borne relapsing fever in a refugee from Mali. Infection. 2017;45(3):373-6.

30. Hertting O, Luth, er J, Giske CG, Bennet R, Eriksson M. Acute infection as cause of hospitalization of asylum-seeking children and adolescents in Stockholm, Sweden 2015-2016. European Journal of Pediatrics. 2021;180(3):893-8.

31. Hoch M, Wieser A, Löscher T, Margos G, Pürner F, Zühl J, et al. Louse-borne relapsing fever (Borrelia recurrentis) diagnosed in 15 refugees from northeast Africa: Epidemiology and preventive control measures, Bavaria, Germany, July to October 2015. Euro Surveill. 2015;20(42).

32. Hytönen J, Khawaja T, Grönroos JO, Jalava A, Meri S, Oksi J. Louse-borne relapsing fever in Finland in two asylum seekers from Somalia. APMIS. 2017;125(1):59-62.

33. Keller C, Zumblick M, Streubel K, Eickmann M, Müller D, Kerwat M, et al. Hemorrhagic diathesis in Borrelia recurrentis infection imported to Germany. Emerg Infect Dis. 2016;22(5):917-9.

34. Lucchini A, Lipani F, Costa C, Scarvaglieri M, Balbiano R, Carosella S, et al. Louseborne relapsing fever among East African refugees, Italy, 2015. Emerg Infect Dis. 2016;22(2):298-301.

35. Osthoff M, Schibli A, Fadini D, Lardelli P, Goldenberger D. Louse-borne relapsing fever - report of four cases in Switzerland, June-December 2015. BMC Infect Dis. 2016;16(1).

36. Ravensbergen SJ, Lokate M, Cornish D, Kloeze E, Ott A, Friedrich AW, et al. High prevalence of infectious diseases and drug-resistant microorganisms in asylum seekers admitted to hospital; no carbapenemase producing Enterobacteriaceae until September 2015. PLoS One. 2016;11(5):e0154791.

37. Schmid M, Dodt C. Multiple organ failure in a young asylum-seeker. Dtsch Arztebl Int. 2017;114(37):625.

38. Wilting KR, Stienstra Y, Sinha B, Braks M, Cornish D, Grundmann H. Louse-borne relapsing fever (Borrelia recurrentis) in asylum seekers from Eritrea, the Netherlands, July 2015. Euro Surveill. 2015;20(30):2-4.

39. Addams J, Lainhart W. The Brief Case: Salmonella enterica Serovar Typhi in a Central American refugee. Clin Microbiol Infect. 2021;59(5).

40. Bliss J, Bouhenia M, Hale P, Couturier BA, Iyer AS, Rumunu J, et al. High prevalence of shigella or enteroinvasive Escherichia coli carriage among residents of an internally displaced persons camp in South Sudan. Am J Trop Med Hyg. 2018;98(2):595-7.

41. Bradarić N, Punda-Polić V, Milas I, Ivić I, Grgić D, Radosević N, et al. Two outbreaks of typhoid fever related to the war in Bosnia and Herzegovina. European Journal of Epidemiology. 1996;12(4):409-12.

42. Elias AF, Peterson SN, Huntington MK. Typhoid fever in a young immigrant child: a case report and review of the literature. South Dakota medicine: the journal of the South Dakota State Medical Association. 2008;61(7):255-8.

43. Kjersem H, Jepsen S, Larsen L, Black F. Salmonella and Shigella carriers among refugees from the Middle East and Sri Lanka in Denmark. Scandinavian Journal of Social Medicine. 1990;18(3):175-8.

44. Martin JA, Mak DB. Changing faces: a review of infectious disease screening of refugees by the Migrant Health Unit, Western Australia in 2003 and 2004. Medical Journal of Australia. 2006;185(11):607-10.

45. Mekonnen GK, Mengistie B, Sahilu G, Kloos H, Mulat W. Etiologies of diarrhea and drug susceptibility patterns of bacterial isolates among under-five year children in refugee camps in Gambella Region, Ethiopia: a case control study. BMC Infect Dis. 2019;19(1).

46. Nyamusore J, Nahimana MR, Ngoc CT, Olu O, Isiaka A, Ndahindwa V, et al. Risk factors for transmission of Salmonella Typhi in Mahama refugee camp, Rwanda: a matched case-control study. Pan African Medical Journal. 2018;29:13.

47. Taylor DN, Echeverria P, Pitarangsi C, Seriwatana J, Sethabutr O, Bodhidatta L, et al. Application of DNA hybridization techniques in the assessment of diarrheal disease among refugees in Thailand. Am J Epidemiol. 1988;127(1):179-87.

48. Taylor R. Typhoid fever in the Basque Refugee Camp. British Medical Journal. 1937;1937:760-1.

49. Yasar AS, Karaman K, Geylan H, Cetin M, Guven B, Oner AF. Typhoid fever accompanied with hematopoetic lymphohistiocytosis and rhabdomyolysis in a refugee child. Journal of Pediatric Hematology Oncology. 2019;41(4):E233-E4.

50. Hershko C, Nesher G, Yinnon AM. Medical problems in Ethiopian refugees airlifted to Israel: Experience in 131 patients admitted to a general hospital. J Trop Med Hyg. 1986;89(3):107-12.

51. Van Boetzelaer E, Fotso A, Angelova I, Huisman G, Thorson T, Hadj-Sahraoui H, et al. Health conditions of migrants, refugees and asylum seekers on search and rescue vessels on the central Mediterranean Sea, 2016-2019: A retrospective analysis. BMJ Open. 2022;12(1). doi:10.1136/bmjopen-2021-053661.

52. Sharara SL, Kanj SS. War and infectious diseases: challenges of the Syrian civil war. PLoS Pathog. 2014;10(10):e1004438. doi: 10.1371/journal.ppat.1004438.

53. Abd Rahman MM, Bryant P, Guppy D, Buttery J, Burgner D. Intermittent fever, splenomegaly and eosinophilia in a recently resettled African refugee. J Paediatr Child Health. 2012;48(10):939-41.

54. Aldulaimi S, Mendez A. Chronic abdominal pain and hepatosplenomegaly in a refugee patient. Travel Med Infect Dis. 2021;41:102009.

55. Beltrame A, Guerriero M, Angheben A, Gobbi F, Requena-Mendez A, Zammarchi L, et al. Accuracy of parasitological and immunological tests for the screening of human schistosomiasis in immigrants and refugees from African countries: An approach with Latent Class Analysis. PLoS Negl Trop Dis. 2017;11(6).

56. Benson J. Asymptomatic schistosomiasis in a young Sudanese refugee. Australian Family Physician. 2007;36(4):249-51.

57. Benzeguir AK, Capraru T, Aust-Kettis A, Björkman A. High frequency of gastrointestinal parasites in refugees and asylum seekers upon arrival in Sweden. Scand J Infect Dis. 1999;31(1):79-82.

58. Borch M, Kiernan M, Rust K, Baron B, Simmons B, Hattala P, et al. Schistosomiasis: a case study. Urol Nurs. 2009;29(1):26-9.

59. Brodine SK, Thomas A, Huang R, Harbertson J, Mehta S, Leake J, et al. Community based parasitic screening and treatment of sudanese refugees: Application and assessment of centers for disease control guidelines. Am J Trop Med Hyg. 2009;80(3):425-30.

60. Buonfrate D, Gobbi F, Marchese V, Postiglione C, Monteiro GB, Giorli G, et al. Extended screening for infectious diseases among newly-arrived asylum seekers from Africa and Asia, Verona province, Italy, April 2014 to June 2015. Eurosurveillance. 2019;23(16):7-14.

61. Ceccarelli G, d'Ettorre G, Riccardo F, Ceccarelli C, Chiaretti M, Picciarella A, et al. Urinary schistosomiasis in asylum seekers in Italy: an emergency currently undervalued. J Immigr Minor Health. 2013;15(4):846-50.

62. Chang AH, Perry S, Du JNT, Agunbiade A, Polesky A, Parsonnet J. Decreasing intestinal parasites in recent northern California refugees. Am J Trop Med Hyg. 2013;88(1):191-7.

63. Chernet A, Kling K, Sydow V, Kuenzli E, Hatz C, Utzinger J, et al. Accuracy of diagnostic tests for Schistosoma mansoni infection in asymptomatic Eritrean refugees: Serology and point-of-care circulating cathodic antigen against stool microscopy. Clinical Infectious Diseases. 2017;65(4):568-74.

64. Daveson J, Macdonald G. A case of periportal fibrosis in a Sudanese refugee. Medical Journal of Australia. 2008;188(11):677-8.

65. Dawson-Hahn EE, Greenberg SLM, Domachowske JB, Olson BG. Eosinophilia and the seroprevalence of schistosomiasis and strongyloidiasis in newly arrived pediatric refugees: An examination of centers for disease control and prevention screening guidelines. Journal of Pediatrics. 2010;156(6):1016-U194.

66. De Vetten G, Dirksen M, Weaver R, Turin T, Aucoin MW. Parasitic stool testing in newly arrived refugees in Calgary, Alta. Canadian Family Physician. 2017;63(12):e518-e25.

67. Edosomwan EU, Evbuomwan IO, Agbalalah C, Dahunsi SO, Abhulimhen-Iyoha BI. Malaria coinfection with Neglected Tropical Diseases (NTDs) in children at Internally Displaced Persons (IDP) camp in Benin City, Nigeria. Heliyon. 2020;6(8).

68. Ehlkes L, George M, Knautz D, Burckhardt F, Jahn K, Vogt M, et al. Negligible import of enteric pathogens by newly-arrived asylum seekers and no impact on incidence of notified Salmonella and Shigella infections and outbreaks in Rhineland-Palatinate, Germany, January 2015 to May 2016. Euro Surveill. 2018;23(20):7-14.

69. Enabulele EE, Platt RN, Adeyemi E, Agbosua E, Aisien MSO, Ajakaye OG, et al. Urogenital schistosomiasis in Nigeria post receipt of the largest single praziquantel donation in Africa. Acta Tropica. 2021:105916.

70. Franco-Paredes C, Dismukes R, Nicolls D, Hidron A, Workowski K, Rodriguez-Morales A, et al. Persistent and untreated tropical infectious diseases among Sudanese refugees in the United States. Am J Trop Med Hyg. 2007;77(4):633-5.

71. Garg PK, Perry S, Dorn M, Hardcastle L, Parsonnet J. Risk of intestinal helminth and protozoan infection in a refugee population. Am J Trop Med Hyg. 2005;73(2):386-91.

72. Geltman PL, Cochran J, Hedgecock C. Intestinal parasites among African refugees resettled in Massachusetts and the impact of an overseas pre-departure treatment program. Am J Trop Med Hyg. 2003;69(6):657-62.

73. Godue CB, Gyorkos TW. Intestinal parasites in refugee claimants: a case study for selective screening? Can J Public Health. 1990;81(3):191-5.

74. Harris AR, Russell RJ, Charters AD. A review of schistosomiasis in immigrants in Western Australia, demonstrating the unusual longevity of Schistosoma mansoni. Trans R Soc Trop Med Hyg. 1984;78(3):385-8.

75. Hofstetter M, Nash TE, Cheever AW. Infection with Schistosoma mekongi in Southeast Asian refugees. J Infect Dis. 1981;144(5):420-6.

76. Jama S, Manivel JC, Abd Alla MD, Stauffer WM. Appendectomy to remember. J Travel Med. 2009;16(4):295-6.

77. Janda A, Eder K, Fressle R, Geweniger A, Diffloth N, Heeg M, et al. Comprehensive infectious disease screening in a cohort of unaccompanied refugee minors in Germany from 2016 to 2017: A cross-sectional study. PLoS Medicine. 2020;17(3).

78. Jenkins-Holick DS, Kaul TL. Schistosomiasis. Urol Nurs. 2013;33(4):163-70.

79. Keittivuti B, D'Agnes T, Keittivuti A, Viravaidya M. Prevalence of schistosomiasis and other parasitic diseases among Cambodian refugees residing in Bang-Kaeng holding center, Prachinburi Province, Thailand. Am J Trop Med Hyg. 1982;31(5):988-90.

80. Keittivuti B, Keittivuti A, D'Agnes T. Schistosomiasis in Cambodian refugees at Ban-Kaeng holding centre, Prachinburi province, Thailand. Southeast Asian J Trop Med Public Health. 1982;13(2):216-9.

81. Keittivuti B, Keittivuti A, O'Rourke T, D'Agnes T. Treatment of Schistosoma mekongi with praziquantel in Cambodian refugees in holding centres in Prachinburi Province, Thailand. Trans R Soc Trop Med Hyg. 1984;78(4):477-9.

82. Keittivuti B, Keittivuti A, O'Rourke TF. Parasitic diseases with emphasis on schistosomiasis in Cambodian refugees, in Prachinburi Province Thailand. Southeast Asian J Trop Med Public Health. 1983;14(4):491-4.

83. Lifson AR, Thai D, O'Fallon A, Mills WA, Hang K. Prevalence of tuberculosis, hepatitis B virus, and intestinal parasitic infections among refugees to Minnesota. Public Health Rep. 2002;117(1):69-77.

84. Ly TDA, Dao TL, Hoang VT, Braunstein D, Brouqui P, Lagier JC, et al. Pattern of infections in French and migrant homeless hospitalised at Marseille infectious disease units, France: A retrospective study, 2017–2018. Travel Med Infect Dis. 2020;36.

85. Maaßen W, Wiemer D, Frey C, Kreuzberg C, Tannich E, Hinz R, et al. Microbiological screenings for infection control in unaccompanied minor refugees: The German Armed Forces Medical Service's experience. Military Medical Research volume. 2017;4(1).

86. Marnell F, Guillet A, Holl, C. A survey of the intestinal helminths of refugees in Juba, Sudan. Annals of Tropical Medicine and Parasitology. 1992;86(4):387-93.

87. Masters PJ, Lanfranco PJ, Sneath E, Wade AJ, Huffam S, Pollard J, et al. Health issues of refugees attending an infectious disease refugee health clinic in a regional Australian hospital. Australian Journal of General Practice. 2018;47(5):305-10.

88. Miller JM, Boyd HA, Ostrowski SR, Cookson ST, Parise ME, Gonzaga PS, et al. Malaria, intestinal parasites, and schistosomiasis among Barawan Somali refugees resettling to the United States: A strategy to reduce morbidity and decrease the risk of imported infections. Am J Trop Med Hyg. 2000;62(1):115-21.

89. Mockenhaupt FP, Barbre KA, Jensenius M, Larsen CS, Barnett ED, Stauffer W, et al. Profile of illness in syrian refugees: A geosentinel analysis, 2013 to 2015. Eurosurveillance. 2016;21(10).

90. Mutch RC, Cherian S, Nemba K, Geddes JS, Rutherford DM, Chaney GM, et al. Tertiary paediatric refugee health clinic in Western Australia: Analysis of the first 1026 children. Journal of Paediatrics and Child Health. 2012;48(7):582-7.

91. Nash TE, Hofstetter M, Cheever AW, Ottesen EA. Treatment of Schistosoma mekongi with praziquantel: a double-blind study. Am J Trop Med Hyg. 1982;31(5):977‐82.

92. Neal PM. Schistosomiasis--an unusual cause of ureteral obstruction: a case history and perspective. Clin Med Res. 2004;2(4):216-27.

93. Neumayr A, Chernet A, Sydow V, Kling K, Kuenzli E, Marti H, et al. Performance of the point-of-care circulating cathodic antigen (POC-CCA) urine cassette test for follow-up after treatment of S. mansoni infection in Eritrean refugees. Travel Medicine and Infectious Disease. 2019;28:59-63.

94. Oboth P, Gavamukulya Y, Barugahare BJ. Prevalence and clinical outcomes of Plasmodium falciparum and intestinal parasitic infections among children in Kiryandongo refugee camp, mid-Western Uganda: A cross sectional study. BMC Infect Dis. 2019;19(1).

95. Ofoezie IE, Asaulu SO, Christensen NØ, Madsen H. Patterns of infection with Schistosoma haematobium in lakeside resettlement communities at the Oyan Reservoir in Ogun State, south-western Nigeria. Ann Trop Med Parasitol. 1997;91(2):187-97.

96. Paran Y, Ben-Ami R, Orlev B, Halutz O, Elalouf O, Wasserman A, et al. Chronic schistosomiasis in African immigrants in Israel: Lessons for the non-endemic setting. Medicine (Baltimore). 2019;98(52).

97. Parenti DM, Lucas D, Lee A, Hollenkamp RH. Health status of Ethiopian refugees in the United States. Am J Public Health. 1987;77(12):1542-3.

98. Patamia I, Nicotra P, Amodeo D, Giuliano L, Cicero CE, Nicoletti A. Geo-helminthiasis among migrants in Sicily: a possible focus for re-emerging neurocysticercosis in Europe. Neurological Sciences. 2017;38(6):1105-7.

99. Paxton GA, Sangster KJ, Maxwell EL, McBride CRJ, Drewe RH. Post-arrival health screening in Karen refugees in Australia. PLoS One. 2012;7(5).

100. Poddighe D, Castelli L, Pulcrano G, Grosini A, Balzaretti M, Spadaro S, et al. Urinary schistosomiasis in an adolescent refugee from Africa: An uncommon cause of hematuria and an emerging infectious disease in Europe. J Immigr Minor Health. 2016;18(5):1237-40.

101. Pohl C, Mack I, Schmitz T, Ritz N. The spectrum of care for pediatric refugees and asylum seekers at a tertiary health care facility in Switzerland in 2015. Eur J Pediatr. 2017;176(12):1681-7.

102. Posey DL, Blackburn BG, Weinberg M, Flagg EW, Ortega L, Wilson M, et al. High prevalence and presumptive treatment of schistosomiasis and strongyloidiasis among African refugees. Clinical Infectious Diseases. 2007;45(10):1310-5.

103. Raman S, Wood N, Webber M, Taylor KA, Isaacs D. Matching health needs of refugee children with services: how big is the gap? Aust N Z J Public Health. 2009;33(5):466-70.

104. Redditt VJ, Janakiram P, Graziano D, Rashid M. Health status of newly arrived refugees in Toronto, Ont: Part 1: infectious diseases. Can Fam Physician. 2015;61(7):e303-e9.

105. Seybolt LM, Christiansen D, Barnett ED. Diagnostic evaluation of newly arrived asymptomatic refugees with eosinophilia. Clinical Infectious Diseases. 2006;42(3):363-7.

106. Sheikh M, Pal A, Wang S, MacIntyre CR, Wood NJ, Isaacs D, et al. The epidemiology of health conditions of newly arrived refugee children: A review of patients attending a specialist health clinic in Sydney. Journal of Paediatrics and Child Health. 2009;45(9):509-13.

107. Shorter D, Makone I, Elliott EJ. Fever and urticaria in an African refugee. Journal of Paediatrics and Child Health. 2006;42(11):731-3.

108. Spicher VM, Genin B, Jordan AR, Rubbia-Brandt L, Le Coultre C. Peritoneal schistosomiasis: an unusual laparoscopic finding. J Pediatr Surg. 2004;39(4):631-3.

109. Sulekova LF, Ceccarelli G, Pombi M, Esvan R, Lopalco M, Vita S, et al. Occurrence of intestinal parasites among asylum seekers in Italy: A cross-sectional study. Travel Med Infect Dis. 2018;27:46-52.

110. Swanson SJ, Phares CR, Mamo B, Smith KE, Cetron MS, Stauffer WM. Albendazole therapy and enteric parasites in United States-bound refugees. New England Journal of Medicine. 2012;366(16):1498-507.

111. Temcharoen P, Viboolyavatana J, Tongkoom B. A survey on intestinal parasitic infections in Laotian refugees at Ubon Province, northeastern Thailand, with special reference to schistosomiasis. Southeast Asian J Trop Med Public Health. 1979;10(4):552-5.

112. Theuring S, Friedrich-Janicke B, Portner K, Trebesch I, Durst A, Dieckmann S, et al. Screening for infectious diseases among unaccompanied minor refugees in Berlin, 2014-2015. Eur J Epidemiol. 2016;31(7):707-10.

113. Tiong ACD, Patel MS, Gardiner J, Ryan R, Linton KS, Walker KA, et al. Health issues in newly arrived African refugees attending general practice clinics in Melbourne. Medical Journal of Australia. 2006;185(11):602-6.

114. Varkey P, Jerath AU, Bagniewski S, Lesnick T. Intestinal parasitic infection among new refugees to Minnesota, 1996-2001. Travel Med Infect Dis. 2007;5(4):223-9.

115. Volkman T, Clifford V, Paxton GA. Schistosoma serology after praziquantel treatment of Schistosoma infection in refugee children resettled in Australia: A retrospective analysis. Travel Medicine and Infectious Disease. 2020;37.

116. Williams B, Boullier M, Cricks Z, Ward A, Naidoo R, Williams A, et al. Screening for infection in unaccompanied asylum-seeking children and young people. Archives of Disease in Childhood. 2020;105(6):530-2.

117. Yauba SM, Rabasa AI, Farouk AG, Elechi HA, Ummate I, Ibrahim BA, et al. Urinary schistosomiasis in Boko Haram-related internally displaced Nigerian children. Saudi J Kidney Dis Transpl. 2018;29(6):1395-402.

118. Zambrano LD, Samson O, Phares C, Jentes E, Weinberg M, Goers M, et al. Unresolved splenomegaly in recently resettled Congolese refugees - multiple states, 2015-2018. MMWR. 2018;67(49):1358-62.

119. Newman RD, Schwartz MA. Hematuria in two school-age refugee brothers from Africa. Pediatr Emerg Care. 1999;15(5):335-7.

120. Summer AP, Stauffer W, Maroushek SR, Nevins TE. Hematuria in children due to schistosomiasis in a nonendemic setting. Clin Pediatr (Phila). 2006;45(2):177-81.

121. Armitage AJ, Cohen J, Heys M, Hardelid P, Ward A, Eisen S. Description and evaluation of a pathway for unaccompanied asylum-seeking children. Archives of disease in childhood. 2022;107(5):456-60. doi:10.1136/archdischild-2021-322319.

122. Aro T, Kantele A. Hospital admissions of refugees, asylum seekers and undocumented migrants: Ten-year retrospective study. Travel Medicine and Infectious Disease. 2021;44. doi:10.1016/j.tmaid.2021.102186.

123. Bergevin A, Husain M, Cruz M, Blanc CL, Dieme A, Girardin ML, et al. Medical check-up of newly arrived unaccompanied minors: A dedicated pediatric consultation service in a hospital. Archives de Pediatrie. 2021;28(8):689-95. doi:10.1016/j.arcped.2021.09.012.

124. Binga WE, Houmsou RS, Garba LC, Amuta EU, Suntaya KL. Use of rivers' water, inadequate hygiene, and sanitation as exposure of internally displaced persons (IDPs) to urogenital schistosomiasis and soil-transmitted helminthiasis in Jalingo Local Government Area (LGA), Taraba State, Nigeria. Journal of Water Sanitation and Hygiene for Development. 2022. doi:10.2166/washdev.2022.089.

125. Bustamante J, Sainz T, Ara-Montojo MF, Almiron MD, Subirats M, Vega DM, et al. Screening for parasites in migrant children. Travel Medicine and Infectious Disease. 2022;47. doi:10.1016/j.tmaid.2022.102287.

126. Bustamante J, Sainz T, Perez S, Rodriguez-Molino P, Vega DM, Mellado MJ, et al. Toxocariasis in migrant children: A 6 years' experience in a reference pediatric unit in Spain. Travel Medicine and Infectious Disease. 2022;47. doi:10.1016/j.tmaid.2022.102288.

127. Carreras-Abad C, Oliveira-Souto I, Pou-Ciruelo D, Pujol-Morro JM, Soler-Palacín P, Soriano-Arandes A, et al. Health and vaccination status of unaccompanied minors after arrival in a European border country: A cross-sectional study (2017-2020). Pediatric Infectious Disease Journal. 2022;41(11):872-7. doi:10.1097/INF.0000000000003670.

128. Cortier M, de La Porte C, Papot E, Goudjo A, Guenneau L, Riou F, et al. Health status and healthcare trajectory of vulnerable asylum seekers hosted in a French reception center. Travel Medicine and Infectious Disease. 2022;46. doi:10.1016/j.tmaid.2021.102180.

129. Geleto GE, Kassa T, Erko B. Epidemiology of soil-transmitted helminthiasis and associated malnutrition among under-fives in conflict affected areas in southern Ethiopia. Tropical Medicine and Health. 2022;50(1). doi:10.1186/s41182-022-00436-1.

130. Hoekstra PT, Chernet A, de Dood CJ, Brienen EAT, Corstjens PLAM, Labhardt ND, et al. Sensitive diagnosis and post-treatment follow-up of Schistosoma mansoni infections in asymptomatic Eritrean refugees by circulating anodic antigen detection and polymerase chain reaction. Am J Trop Med Hyg. 2022;106(4):1240-6. doi:10.4269/ajtmh.21-0803.

131. Kumar GS, Pezzi C, Payton C, Mamo B, Urban K, Scott K, et al. Health of asylees compared to refugees in the United States using domestic medical examination data, 2014-2016: A cross-sectional analysis. Clinical Infectious Diseases. 2021;73(8):1492-9. doi:10.1093/cid/ciab502.

132. Müller F, Chandra S, Bogoch II, Rashid M, Redditt V. Intestinal parasites in stool testing among refugees at a primary care clinic in Toronto, Canada. BMC Infect Dis. 2022;22(1). doi:10.1186/s12879-022-07226-4.

133. Tamarozzi F, Ursini T, Ronzoni N, Monteiro GB, Gobbi FG, Angheben A, et al. Prospective cohort study using ultrasonography of Schistosoma haematobium–infected migrants. Journal of Travel Medicine. 2021;28(6). doi:10.1093/jtm/taab122.

134. Zöllkau J, Ankert J, Pletz MW, Mishra S, Seliger G, Lobmaier SM, et al. Hepatitis E, schistosomiasis and echinococcosis–prevalence in a cohort of pregnant migrants in Germany and their influence on fetal growth restriction. Pathogens. 2022;11(1). doi:10.3390/pathogens11010058.

135. Johnston V, Smith L, Roydhouse H. The health of newly arrived refugees to the Top End of Australia: Results of a clinical audit at the Darwin refugee health service. Aust J Prim Health. 2012;18(3):242-7.

136. Southwood T, Davidson GP, Phillips GE, Rice M. Hepatosplenic schistosomiasis in a South-East Asian refugee child in South Australia. Aust N Z J Med. 1983;13(4):384-6.

137. Persson A, Rombo L. Intestinal parasites in refugees and asylum seekers entering the Stockholm area, 1987-88: Evaluation of routine stool screening. 1994;26(2):199-207.

138. Lowther SA, Johnson G, Hendel-Paterson B, Nelson K, Mamo B, Krohn K, et al. HIV/AIDS and associated conditions among HIV-infected refugees in Minnesota, 2000–2007. Int J Environ Res Public Health. 2012;9(11):4197-209. doi: 10.3390/ijerph9114197.

139. Harkensee C, Andrew R. Health needs of accompanied refugee and asylum-seeking children in a UK specialist clinic. Acta Paediatr. 2021;110(8):2396-404. doi:10.1111/apa.15861.

140. Zwi K, Morton N, Woodland L, Mallitt K-A, Palasanthiran P. Screening and primary care access for newly arrived paediatric refugees in regional Australia: A 5 year cross-sectional analysis (2007–12). Journal of Tropical Pediatrics. 2016;63(2):109-17. doi: 10.1093/tropej/fmw059.

141. Chaves NJ, Gibney KB, Leder K, O'Brien DP, Marshall C, Biggs BA. Screening practices for infectious diseases among Burmese refugees in Australia. Emerg Infect Dis. 2009;15(11):1769-72. doi: 10.3201/eid1511.090777.

142. Caruana SR, Kelly HA, Ngeow JYY, Ryan NJ, Bennett CM, Chea L, et al. Undiagnosed and potentially lethal parasite infections among immigrants and refugees in Australia. Journal of Travel Medicine. 2006;13(4):233-9.

143. Beltrame A, Buonfrate D, Gobbi F, Angheben A, Marchese V, Monteiro GB, et al. The hidden epidemic of schistosomiasis in recent African immigrants and asylum seekers to Italy. European Journal of Epidemiology. 2017;32(8):733-5.

144. Quandelacy TM, Riefkohl A, Franco-Paredes C. Prevalence of untreated schistosomiasis among Sudanese refugees: “The Lost Boys of Sudan” in the United States. Boletin medico del Hospital Infantil de Mexico. 2010;67:503-6.

145. Ryan N, Plackett M, Dwyer B. Parasitic infections of refugees. Medical Journal of Australia. 1988;148(10):491-4. doi:10.5694/j.1326-5377.1988.tb99455.x.

146. Watts NS, Mizinduko MM, Barnett ED, White LF, Hochberg NS. Association between parasitic infections and tuberculin skin test results in refugees. Travel Medicine and Infectious Disease. 2017;16:35-40.

147. Richter J, Bode JG, Blondin D, Kircheis G, Kubitz R, Holtfreter MC, et al. Severe liver fibrosis caused by Schistosoma mansoni: Management and treatment with a transjugular intrahepatic portosystemic shunt. The Lancet Infectious Diseases. 2015;15(6):731-7.

148. Haq KUA, Gul NA, Hammad HM, Bibi Y, Bibi A, Mohsan J. Prevalence of Giardia intestinalis and Hymenolepis nana in Afghan refugee population of Mianwali district, Pakistan. Afr Health Sci. 2015;15(2):394-400.

149. Agha Rodina AI, Teoderescu I. Prevalence of intestinal parasites in three localities in Gaza Governorates - Palestine. Archives of Public Health. 2002;60(6):363-70.

150. Ahmed W, Ahmad M, Rafatullah, Shah F, Sajadullah. Pervasiveness of intestinal protozoan and worm incursion in IDP's (North Waziristan agency, KPK-Pakistan) children of 6-16 years. Journal of the Pakistan Medical Association. 2015;65(9):943-5.

151. Al-Hindi AI, Abu Shammala BM. Dientamoeba fragilis in gaza strip: A neglected protozoan parasite. Iran J Parasitol. 2013;8(2):249-55.

152. Arfaa F. Intestinal parasites among Indochinese refugees and Mexican immigrants resettled in Contra Costa County, California. J Fam Pract. 1981;12(2):223-6.

153. Arthur JD, Bodhidatta L, Echeverria P, Phuphaisan S, Paul S. Diarrheal disease in Cambodian children at a camp in Thailand. Am J Epidemiol. 1992;135(5):541-51..

154. Chandrasena TGAN, Hapuarachchi HC, Dayanath MYD, Pathmeswaran A, De Silva NR. Intestinal parasites and the growth status of internally displaced children in Sri Lanka. Trop Doct. 2007;37(3):163-5.

155. D'Alauro F, Lee RV, Pao-In K, Khairallah M. Intestinal parasites and pregnancy. Infect Dis Obstet Gynecol. 1985;66(5):639-43.

156. DeGirolami PC, Kimber J. Intestinal parasites among Southeast Asian refugees in Massachusetts. Am J Clin Pathol. 1983;79(4):502-4.

157. Goswami ND, Shah JJ, Corey GR, Stout JE. Short report: Persistent eosinophilia and Strongyloides infection in Montagnard refugees after presumptive albendazole therapy. Am J Trop Med Hyg. 2009;81(2):302-4.

158. Gyorkos TW, Frappier-Davignon L, MacLean JD, Viens P. Effect of screening and treatment on imported intestinal parasite infections: Results from a randomized, controlled trial. Am J Epidemiol. 1989;129(4):753-61.

159. Gyorkos TW, MacLean JD, Viens P, Chheang C, Kokoskin-Nelson E. Intestinal parasite infection in the Kampuchean refugee population 6 years after resettlement in Canada. Infect Dis. 1992;166(2):413-7.

160. Hassan AO, Mero WMS. Prevalence of intestinal parasites among displaced people living in displacement camps in duhok province/Iraq. Internet Journal of Microbiology. 2020;17(1).

161. Heudorf U, Karathana M, Krackhardt B, Huber M, Raupp P, Zinn C. Surveillance for parasites in unaccompanied minor refugees migrating to Germany in 2015. Gms Hygiene and Infection Control. 2016;11:3.

162. Hoffman SL, Barrett-Connor E, Norcross W, Nguyen D. Intestinal parasites in Indochinese immigrants. Am J Trop Med Hyg. 1981;30(2):340-3.

163. Jones MJ, Thompson Jr JH, Brewer NS. Infectious diseases of Indochinese refugees. Mayo Clin Proc. 1980;55(8):482-8.

164. Kortas AZ, Polenz J, von Hayek J, Rüdiger S, Rottbauer W, Storr U, et al. Screening for infectious diseases among asylum seekers newly arrived in Germany in 2015: a systematic single-centre analysis. Public Health. 2017;153:1-8.

165. Lerman D, Barrett-Connor E, Norcross W. Intestinal parasites in asymptomatic adult Southeast Asian immigrants. J Fam Pract. 1982;15(3):443-6.

166. Meropol SB. Health status of pediatric refugees in Buffalo, NY. Arch Pediatr Adolesc Med. 1995;149(8):887-92.

167. Miladinovic-Tasic NL, Tasic SA, Kranjcic-Zec I, Tasic G, Tasic A, Tasic IS. Asymptomatic giardiasis-more prevalent in refugees than in native inhabitants of the city of Nis, Serbia. Central European Journal of Medicine. 2008;3(2):203-6.

168. Mitchell T, Lee D, Weinberg M, Phares C, James N, Amornpaisarnloet K, et al. Impact of enhanced health interventions for United States-bound refugees: evaluating best practices in migration health. Am J Trop Med Hyg. 2018;98(3):920-8.

169. Molina CD, Molina MM, Molina JM. Intestinal parasites in Southeast Asian refugees two years after immigration. West J Med. 1988;149(4):422-5.

170. Parish RA. Intestinal parasites in Southeast Asian refugee children. West J Med. 1985;143(1):47-9.

171. Relić T, Kačarević H, Ilić N, Jovanović D, Tambur Z, Doder R, et al. Intestinal parasitosis in asylum seekers from the middle east and South Asia. Vojnosanitetski pregled. 2018;75(11):1101-5.

172. Sullivan R, Linneman Jr CC, Clark CS, Walzer PD. Seroepidemiologic study of giardiasis patients and high-risk groups in a midwestern city in the United States. Am J Public Health. 1987;77(8):960-3.

173. Tittle BS, Harris JA, Chase PA. Health screening of Indochinese refugee children. Am J Dis Child. 1982;136(8):697-700.

174. Ul Haq KA, Gul NA, Muhammad Hammad H, Bibi Y, Bibi A, Mohsan J. Prevalence of giardia intestinalis and hymenolepis nana in afghan refugee population of mianwali district, pakistan. African Health Sciences. 2015;15(2):394-400.

175. Wiesenthal AM, Nickels MK, Hashimoto KG. Intestinal parasites in Southeast-Asian refugees. Prevalence in a community of Laotians. JAMA. 1980;244(22):2543-4.

176. Abu Mourad TA. Palestinian refugee conditions associated with intestinal parasites and diarrhoea: Nuseirat refugee camp as a case study. Public Health. 2004;118(2):131-42.

177. Gozalbo M, Guillen M, Taroncher-Ferrer S, Cifre S, Carmena D, Soriano JM, et al. Assessment of the nutritional status, diet and intestinal parasites in hosted Saharawi children. Children (Basel). 2020;7(12):18.

178. Evbuomwan IO, Edosomwan EU, Idubor V, Bazuaye C, Abhulimhen-Iyoha BI, Adeyemi OS, et al. Survey of intestinal parasitism among schoolchildren in internally displaced persons camp, Benin City, Nigeria. Scientific African. 2022;17. doi:10.1016/j.sciaf.2022.e01373.

179. Webster JL, Stauffer WM, Mitchell T, Lee D, O’Connell EM, Weinberg M, et al. Cross-sectional assessment of the association of eosinophilia with intestinal parasitic infection in U.S.-bound refugees in Thailand: Prevalent, age dependent, but of limited clinical utility. Am J Trop Med Hyg. 2022;106(5):1552-9. doi:10.4269/ajtmh.21-0853.

180. Samuda GM, Chan SP, Yeung CY. Vietnamese child health in a Hong Kong closed camp. Aust Paediatr J. 1988;24(2):115-7.

181. Francke E. Medical evaluation of Indochinese refugees conditions to consider. Postgraduate Medicine. 1982;72(5):92-3.

182. Bedard B, Pennise M, Weimer AC, Kennedy BS. Magnitude of Giardia cases among refugees, adoptees and immigrants in Monroe County, New York, 2003-2013. International Journal of Migration, Health and Social Care. 2016;12(3):211-5.

183. Ekdahl K, Andersson Y. Imported giardiasis: Impact of international travel, immigration, and adoption. Am J Trop Med Hyg. 2005;72(6):825-30.

184. Lurio J, Verson H, Karp S. Intestinal parasites in Cambodians: comparison of diagnostic methods used in screening refugees with implications for treatment of populations with high rates of infestation. J Am Board Fam Pract. 1991;4(2):71-8. Epub 1991/03/01.

185. Alawieh A, Musharrafieh U, Jaber A, Berry A, Ghosn N, Bizri AR. Revisiting leishmaniasis in the time of war: the Syrian conflict and the Lebanese outbreak. International Journal of Infectious Diseases. 2014;29:115-9.

186. Alhawarat M, Khader Y, Shadfan B, Kaplan N, Iblan I. Trend of Cutaneous Leishmaniasis in Jordan from 2010 to 2016: Retrospective Study. JMIR Public Health and Surveillance. 2020;6(1).

187. Al-Jawabreh A, Barghuthy F, Schnur LF, Jacobson RL, Schönian G, Abdeen Z. Epidemiology of cutaneous leishmaniasis in the endemic area of Jericho, Palestine. East Mediterr Health J. 2003;9(4):805-15.

188. Amr ZS, Kanani K, Shadfan B, Hani RB. Cutaneous leishmaniasis among Syrian refugees in Jordan: a retrospective study. Bull Soc Pathol Exot. 2018;111(5):295-300.

189. Bizri NA, Alam W, Khoury M, Musharrafieh U, Ghosn N, Berri A, et al. The association between the Syrian crisis and cutaneous leishmaniasis in Lebanon. Acta Parasitologica. 2021:1-6.

190. Bradshaw S, Litvinov IV. Dermal leishmaniasis in a 25-year-old Syrian refugee. Canadian Medical Association Journal. 2017;189(45):E1397-E.

191. Brooker S, Mohammed N, Adil K, Agha S, Reithinger R, Rowl, et al. Leishmaniasis in refugee and local Pakistani populations. Emerg Infect Dis. 2004;10(9):1681-4.

192. Chalupa P, Vanista J, Burget I, Stary J, Sukova M, Nohynkova M. The review of imported visceral leishmaniosis in the Czech Republic. Bratislavské lekárske listy. 2001;102(2):84-91.

193. Çizmeci Z, Karakuş M, Karabela ŞN, Erdoğan B, Güleç N. Leishmaniasis in Istanbul; A new epidemiological data about refugee leishmaniasis. Acta Trop. 2019;195:23-7.

194. Crogan J, Gunasekera H, Wood N, Sheikh M, Isaacs D. Management of old world cutaneous leishmaniasis in refugee children. Pediatr Infect Dis J. 2010;29(4):357-9.

195. Doganay M, Demiraslan H. Refugees of the Syrian Civil War: Impact on reemerging infections, health services, and biosecurity in Turkey. Health Secur. 2016;14(4):220-5.

196. Dunya G, Habib R, Moukarbel RV, Khalifeh I. Head and neck cutaneous leishmania: clinical characteristics, microscopic features and molecular analysis in a cohort of 168 cases. Eur Arch Otorhinolaryngol. 2016;273(11):3819-26.

197. Eksi F, Ozgoztasi O, Karsligil T, Saglam M. Genotyping Leishmania promastigotes isolated from patients with cutaneous leishmaniasis in south-eastern Turkey. Journal of International Medical Research. 2016;45(1):114-22.

198. El Hajj R, El Hajj H, Khalifeh I. Fatal visceral leishmaniasis caused by Leishmania infantum, Lebanon. Emerg Infect Dis. 2018;24(5):906-7.

199. El Safadi D, Merhabi S, Rafei R, Mallat H, Hamze M, Acosta-Serrano A. Cutaneous leishmaniasis in north Lebanon: Re-emergence of an important neglected tropical disease. Trans R Soc Trop Med Hyg. 2019;113(8):471-6.

200. Eroglu F, Ozgoztasi O. The increase in neglected cutaneous leishmaniasis in Gaziantep province of Turkey after mass human migration. Acta Trop. 2019;192:138-43.

201. Gurses G, Ozaslan M, Zeyrek FY, Kilic IH, Doni NY, Karagoz ID, et al. Molecular identification of Leishmania spp. isolates causes cutaneous leishmaniasis (CL) in Sanliurfa Province, Turkey, where CL is highly endemic. Folia Microbiol (Praha). 63(3):353-9.

202. Hijawi KJF, Hijjawi NS, Ibbini JH. Detection, genotyping, and phylogenetic analysis of Leishmania isolates collected from infected Jordanian residents and Syrian refugees who suffered from cutaneous leishmaniasis. Parasitol Res. 2019;118(3):793-805.

203. Hussain M, Munir S, Jamal MA, Ayaz S, Akhoundi M, Mohamed K. Epidemic outbreak of anthroponotic cutaneous leishmaniasis in Kohat District, Khyber Pakhtunkhwa, Pakistan. Acta Tropica. 2017;172:147-55.

204. Inci R, Ozturk P, Mulayim MK, Ozyurt K, Alatas ET, Inci MF. Effect of the Syrian civil war on prevalence of cutaneous leishmaniasis in Southeastern Anatolia, Turkey. Medical Science Monitor. 2015;21:5.

205. Jacoby H, Rawling RA, Granato PA. Cutaneous leishmaniasis in a Central American refugee. Clinical Microbiology Newsletter. 2014;36(3):22-4.

206. Jamal Q, Shah A, Ali N, Ashraf M, Awan MM, Lee CM. Prevalence and comparative analysis of cutaneous leishmaniasis in Dargai Region in Pakistan. Pakistan Journal of Zoology. 2013;45(2):537-41.

207. Kanani K, Amr ZS, Shadfan B, Khorma R, Rø G, Abid M, et al. Cutaneous leishmaniasis among Syrian refugees in Jordan. Acta Tropica. 2019;194:169-71.

208. Khan MI, Muhammad M, Khan W, Khan N, Noor SM. Nasal involvement in cutaneous leishmaniasis. Journal of Postgraduate Medical Institute. 2010;24(3):202-6.

209. Kolaczinski J, Brooker S, Reyburn H, Rowland M. Epidemiology of anthroponotic cutaneous leishmaniasis in Afghan refugee camps in northwest Pakistan. Trans R Soc Trop Med Hyg. 2004;98(6):373-8.

210. Lindner AK, Richter J, Gertler M, Nikolaus M, Martinez GE, Muller K, et al. Cutaneous leishmaniasis in refugees from Syria: complex cases in Berlin 2015-2020. Journal of Travel Medicine. 2020;27(7):8.

211. Marlet MVL, Wuillaume F, Jacquet D, Quispe KW, Dujardin JC, Boelaert M. A neglected disease of humans: A new focus of visceral leishmaniasis in Bakool, Somalia. Trans R Soc Trop Med Hyg. 2003;97(6):667-71.

212. Motamedi MH, Har, i P, Azizi T. Leishmaniasis of the face: Report of a case. Indian Journal of Dermatology. 2009;54(5):S37-S40.

213. Ntais P, Christodoulou V, Tsirigotakis N, Dokianakis E, Dedet J-P, Pratlong F, et al. Will the introduction of Leishmania tropica MON-58, in the island of Crete, lead to the settlement and spread of this rare zymodeme? Acta Tropica. 2014;132:125-30. doi:10.1016/j.actatropica.2014.01.003.

214. Peeters E, Verhulst S, Wojciechowski M, Vlieghe E, Jorens P, Van Marck V, et al. Visceral leishmaniasis in a child infected with the human immunodeficiency virus in a non-endemic region. Trop Pediatr. 2011;57(6):493-5.

215. Perea WA, Ancelle T, Moren A, Nagelkerke M, Sondorp E. Visceral leishmaniasis in southern Sudan. Trans R Soc Trop Med Hyg. 1991;85(1):48-53.

216. Rab MA, al Rustamani L, Bhutta RA, Mahmood MT, Evans DA. Cutaneous leishmaniasis: iso-enzyme characterisation of Leishmania tropica. J Pak Med Assoc. 1997;47(11):270-3.

217. Rowland M, Munir A, Durrani N, Noyes H, Reyburn H. An outbreak of cutaneous leishmaniasis in an Afghan refugee settlement in north-west Pakistan. Trans R Soc Trop Med Hyg. 1999;93(2):133-6.

218. Saab M, El Hage H, Charafeddine K, Habib RH, Khalifeh I. Diagnosis of cutaneous leishmaniasis: Why punch when you can scrape? Am J Trop Med Hyg. 2015;92(3):518-22.

219. Saroufim M, Charafeddine K, Issa G, Khalifeh H, Habib RH, Berry A, et al. Ongoing epidemic of cutaneous leishmaniasis among Syrian refugees, Lebanon. Emerg Infect Dis. 2014;20(10):1712-5.

220. Storer E, Wayte J. Cutaneous leishmaniasis in Afghani refugees. Australasian Journal of Dermatology. 2005;46(2):80-3.

221. Sulaiman AA, Elmadhoun WM, Noor SK, Bushara SO, Almobarak AO, Awadalla H, et al. An outbreak of cutaneous leishmaniasis among a displaced population in North Sudan: Review of cases. J Family Med Prim Care. 2019;8(2):556-63.

222. Van Kesteren L, Maniewski U, Bottieau E, Cnops L, Huits R. Cutaneous leishmaniasis in syrian refugee children: A case series. Pediatr Infect Dis J. 2020:E154-E6.

223. Wollina U, Koch A, Guarneri C, Tchernev G, Lotti T. Cutaneous leishmaniasis – A case series from Dresden. Open Access Maced J Med Sci. 2018;6(1):89-92.

224. Yentur Doni N, Gurses G, Dikme R, Aksoy M, Yildiz Zeyrek F, Simsek Z, et al. Cutaneous leishmaniasis due to three Leishmania species among Syrian refugees in Sanliurfa, Southeastern Turkey. Acta Parasitol. 2020;65(4):936-48.

225. Zijlstra EE, Ali MS, El-Hassan AM, El-Toum IA, Satti M, Ghalib Kager HWPA. Direct agglutination test for diagnosis and sero- epidemiological survey of kala-azar in the Sudan. Trans R Soc Trop Med Hyg. 1991;85(4):474-6.

226. Zijlstra EE, Siddig Ali M, El-Hassan AM, El-Toum IA, Satti M, Ghalib HW, et al. Kala-azar in displaced people from southern Sudan: Epidemiological, clinical and therapeutic findings. Trans R Soc Trop Med Hyg. 1991;85(3):365-9.

227. Khachfe HH, Zayyoun FJ, Sharif-Askari E, Ramadan W, Hallal N, Khachfe HM. Effect of leishmaniasis on the performance of elementary school students: A case study among syrian refugees in some bekaa (lebanon) area schools. J Epidemiol Glob Health. 2019;9(4):266-73.

228. Özbilgin A, Gencoglan G, Tunali V, Çavuş İ, Yıldırım A, Gündüz C, et al. Refugees at the crossroads of continents: A molecular approach for cutaneous leishmaniasis among Refugees in Turkey. Acta Parasitologica. 2020;65(1):136-43.

229. Altinel Y, Tas B. How to predict the diagnosis of cutaneous leishmaniasis in a non-endemic Region. Indian Journal of Dermatology. 2022;67(3):232-8. doi:10.4103/ijd.IJD_452_20.

230. Debus D, Genç S, Kurz P, Holzer M, Bauer K, Heimke-Brinck R, et al. Case report: Local treatment of a Leishmania tropica infection in a Syrian child with a novel filmogenic preparation of pharmaceutical sodium chlorite. Am J Trop Med Hyg. 2022;106(3):857-60. doi:10.4269/ajtmh.21-0962.

231. Hammoud S, Onchonga D, Amer F, Kocsis B. The burden of communicable diseases in Lebanon: Trends in the past decade. Disaster Medicine and Public Health Preparedness. 2022;16(5):1725-7. doi:10.1017/dmp.2021.200.

232. Lemieux A, Lagacé F, Billick K, Ndao M, Yansouni CP, Semret M, et al. Cutaneous leishmaniasis in travellers and migrants: a 10-year case series in a Canadian reference centre for tropical diseases. CMAJ open. 2022;10(2):E546-E53. doi:10.9778/cmajo.20210238.

233. Barbier D, Demenais F, Lefait JF, David B, Blanc M, Hors J, et al. Susceptibility to human cutaneous leishmaniasis and HLA, Gm, Km markers. Tissue Antigens. 1987;30(2):63-7.

234. Saikal SL, Ge L, Mir A, Pace J, Abdulla H, Leong KF, et al. Skin disease profile of Syrian refugees in Jordan: a field-mission assessment. J Eur Acad Dermatol Venereol. 2020;34(2):419-25. doi: 10.1111/jdv.15909.

235. Koçarslan S, Turan E, Ekinci T, Yesilova Y, Apari R. Clinical and histopathological characteristics of cutaneous Leishmaniasis in Sanliurfa City of Turkey including Syrian refugees. Indian J Pathol Microbiol. 2013;56(3):211-5. doi: 10.4103/0377-4929.120367.

236. de Beer P, el Harith A, van Grootheest M, Winkler A. Outbreak of kala-azar in the Sudan. Lancet. 1990;335(8683):224. doi: 10.1016/0140-6736(90)90313-t.

237. Brooks AMV, Essex WB, West RH. Cysticercosis of the superior oblique muscle. Aust J Ophthalmol. 1983;11(2):119-22.

238. Dao AH, Gregory DW, McKee LC. Specific health problems of Southeast Asian refugees in middle Tennessee. Southern Medical Journal. 1984;77(8):995-7.

239. Fritzsche M, Gottstein B, Wigglesworth MC, Eckert J. Serological survey of human cysticercosis in Irianese refugee camps in Papua New Guinea. Acta Tropica. 1990;47(2):69-77.

240. Lucey JM, McCarthy J, Burgner DP. Encysted seizures: status epilepticus in a recently resettled refugee child. Medical Journal of Australia. 2010;192(4):237.

241. McCleery EJ, Patchanee P, Pongsopawijit P, Chailangkarn S, Tiwananthagorn S, Jongchansittoe P, et al. Taeniasis among refugees living on Thailand–Myanmar border, 2012. Emerg Infect Dis. 2015;21(10):1824-6.

242. O'Neal SE, Robbins NM, Townes JM. Neurocysticercosis among resettled refugees from Burma. J Travel Med. 2012;19(2):118-21.

243. O'Neal SE, Townes JM, Wilkins PP, Noh JC, Lee D, Rodriguez S, et al. Seroprevalence of antibodies against Taenia solium cysticerci among refugees resettled in United States. Emerg Infect Dis. 2012;18(3):431-8.

244. Shen C, Li S, Zheng S, Choi MH, Bae YM, Hong ST. Tissue parasitic helminthiases are prevalent at Cheongjin, North Korea. Korean J Parasitol. 2007;45(2):139-44.

245. Yeaney GA, Kolar BS, Silberstein HJ, Wang HZ. Case 163: Solitary neurocysticercosis. Radiology. 2010;257(2):581-5.

246. McDowell D, Harper CG. Neurocysticercosis - Two Australian cases. Med J Aust. 1990;152(4):217-8.

247. Angheben A, Mariconti M, Degani M, Gobbo M, Palvarini L, Gobbi F, et al. Is there echinococcosis in West Africa? A refugee from Niger with a liver cyst. Parasit Vectors. 2017;10(1).

248. Bjazevic J, Golomb D, Silverman MS, Pautler SE, Razvi H. Case report - Primary renal echinococcal infection. Can Urol Assoc J. 2020;14(8):E383-E6.

249. Engström ELS, Salih GN, Wiese L. Seronegative, complicated hydatid cyst of the lung: A case report. Respir Med Case Rep. 2017;21:96-8.

250. Franco-Paredes C, Nicolls D, Kempker R, Dismukes R, Kozarsky P. Pelvic echinococcosis in a Northern Iraqi refugee. J Travel Med. 2006;13(2):119-22.

251. Heendeniya A, Bogoch I. Multiple hepatic hydatid cysts in an Iraqi refugee. Am J Trop Med Hyg. 2018;99(5):1107.

252. Richter J, Esmann L, Lindner AK, Trebesch I, Equihua-Martinez G, Niebank M, et al. Cystic echinococcosis in unaccompanied minor refugees from Afghanistan and the Middle East to Germany, July 2016 through June 2017. European Journal of Epidemiology. 2019;34(6):611-2.

253. Tappe D, Weise D, Ziegler U, Müller A, Müllges W, Stich A. Brain and lung metastasis of alveolar echinococcosis in a refugee from a hyperendemic area. J Med Microbiol. 2008;57(11):1420-3.

254. Yasin AM, Esa HAH, Hameed AA, Wahid W, Ahamed POS. First case of pulmonary hydatid cyst in a pregnant syrian refugee woman in malaysia. Med J Malaysia. 2021;76(1):103-6.

255. Zahedi D, Moori P, Ashraf I, Hafeez I. Pleuritic chest pain in a young asylum seeker. Breathe (Sheff). 2020;16(1):190294.

256. Azlin MY, Esa HAH, Hameed AA, Wahid W, Pakeer O. First case of pulmonary hydatid cyst in a pregnant Syrian refugee woman in Malaysia. Med J Malaysia. 2021;76(1):103-6.

257. Mumtaz K, Kamani L, Chawla T, Hamid S, Jafri W. Hepatic cystic echinococcosis: clinical characteristics and outcomes in Pakistan. 2009;39(4):215-7.

258. Çoşkun B, Gülümser Ç, Çoşkun B, Artuk C, Karaşahin KE. Impact of Syrian refugees on congenital TORCH infections screening in Turkey. J Obs and Gynae Research. 2020;46(7):1017-24.

259. Fan CK, Liao CW, Wu MS, Su KE, Han BC. Seroepidemiology of Toxoplasma gondii infection among Chinese aboriginal and Han people residing in mountainous areas of northern Thailand. J Parasitol. 2003;89(6):1239-42.

260. Halici-Ozturk F, Yakut K, Öcal FD, Erol A, Gökay S, Çağlar AT, et al. Seroprevalence of Toxoplasma gondii infections in Syrian pregnant refugee women in Turkey. European Journal of Obstetrics and Gynecology and Reproductive Biology. 2021;256:91-4.

261. Schroeder Jr HW, Yarrish RL, Perkins TF, Lee C. Sequential disseminated tuberculosis and toxoplasmosis in a Haitian refugee. Southern Medical Journal. 1984;77(4):533-4.

262. Schweickert B, Bollmann R, Loui A, Kaufmann O, Kluttig L, Feiterna-Sperling C, et al. Fatal disseminated toxoplasmosis with congenital transmission in an African migrant. AIDS (London, England). 2008;22(12):1523-5.

263. Van Enter BJD, Lau YL, Ling CL, Watthanaworawit W, Sukthana Y, Lee WC, et al. Seroprevalence of toxoplasma gondii infection in refugee and migrant pregnant women along the Thailand-myanmar border. Am J Trop Med Hyg. 2017;97(1):232-5.

264. Aksin S, Cim N, Andan C, Tunc S, Goklu MR. Comparison of obstetric and infectious results among Syrian pregnant women. Annals of Clinical and Analytical Medicine. 2021;12(5):501-5. doi:10.4328/acam.20411.

265. Hansu K, Özdemir H, Hansu İ, Çıkım G, Tok A. Suriyeli Mülteci ve Türk Yerleşik Gebelerde Toksoplazma Seroprevalansının Karşılaştırılması. Turkiye parazitolojii dergisi. 2021;45(4):247-51. doi:10.4274/tpd.galenos.2021.36855.

266. Dorkenoo MA, Tchankoni MK, Yehadji D, Yakpa K, Tchalim M, Sossou E, et al. Monitoring migrant groups as a post-validation surveillance approach to contain the potential reemergence of lymphatic filariasis in Togo. Parasites & Vectors. 2021;14(1).

267. Montour J, Lee D, Snider C, Jentes ES, Stauffer W. Absence of Loa loa microfilaremia among newly arrived congolese refugees in Texas. Am J Trop Med Hyg. 2017;97(6):1833-5.

268. Yangco BG, Vincent AL, Vickery AC. A survey of filariasis among refugees in South Florida. Am J Trop Med Hyg. 1984;33(2):246-51.

269. Zhao Y, Alex, er B, Bailey JA, Welch L, Greene M, et al. Therapeutic apheresis using a mononuclear cell program to lower the microfilaria burden of a 23-year-old African woman with loiasis. J Clin Apher. 2017;32(3):200-2.

270. Ahmed T, Maheswary NP, Khan NI. Filariasis in Mirpur area of Dhaka city. Bangladesh Med Res Counc Bull. 1986;12(2):83-94.

271. Gumisiriza N, Kugler M, Brusselaers N, Mubiru F, Anguzu R, Ningwa A, et al. Risk factors for nodding syndrome and other forms of epilepsy in northern uganda: A case-control study. Pathogens. 2021;10(11). doi:10.3390/pathogens10111451.

272. (CDC) CfDCaP. Imported dracunculiasis--United States, 1995 and 1997. MMWR. 1998;47(11):209-11.

273. McAuley JB, Michelson MK, Hightower AW, Engeran S, Wintermeyer LA, Schantz PM. A trichinosis outbreak among Southeast Asian refugees. Am J Epidemiol. 1992;135(12):1404-10.

274. Stehr-Green JK, Schantz PM. Trichinosis in Southeast Asian refugees in the United States. Am J Public Health. 1986;76(10):1238-9.

275. Abu-Alrub SM, Abusada GM, Farraj MA, Essawi TA. Prevalence of Cryptosporidium spp. in children with diarrhoea in the West Bank, Palestine. J Infect Dev Ctries. 2008;2(1):59-62.

276. Cherian P, Junckerstorff RK, Rosen D, Kumarasinghe P, Morling A, Tuch P, et al. Late-stage human African trypanosomiasis in a Sudanese refugee. Medical Journal of Australia. 2010;192(7):417-9.

277. Sahlas DJ, Dick MacLean J, Janevski J, Detsky AS. Clinical problem-solving. Out of Africa. N Engl J Med. 2002;347(10):749-53.

278. Steele LS, MacPherson DW, Kim J, Keystone JS, Gushulak BD. The sero-prevalence of antibodies to Trypanosoma cruzi in Latin American refugees and immigrants to Canada. Journal of Immigrant and Minority Health. 2007;9(1):43-7. doi: 10.1007/s10903-006-9014-x.

279. Enterically transmitted non-A, non-B hepatitis--East Africa. MMWR. 1987;36(16):241-4.

280. Hepatitis E, Chad. Weekly epidemiological record / Health Section of the Secretariat of the League of Nations. 2004;79(35):313.

281. Ahmed JA, Moturi E, Spiegel P, Schilperoord M, Burton W, Kassim NH, et al. Hepatitis E outbreak, Dadaab refugee camp, Kenya, 2012. Emerg Infect Dis. 2013;19(6):1010-2.

282. Azman AS, Bouhenia M, Iyer AS, Rumunu J, Laku RL, Wamala JF, et al. High hepatitis E seroprevalence among displaced persons in South Sudan. Am J Trop Med Hyg. 2017;96(6):1296-301.

283. Boccia D, Guthmann JP, Klovstad H, Hamid N, Tatay M, Ciglenecki I, et al. High mortality associated with an outbreak of hepatitis E among displaced persons in Darfur, Sudan. Clinical Infectious Diseases. 2006;42(12):1679-84.

284. Browne LB, Menkir Z, Kahi V, Maina G, Asnakew S, Tubman M, et al. Hepatitis E outbreak among refugees from South Sudan - Gambella, Ethiopia, April 2014-January 2015. MMWR. 2015;64(19):537.

285. Chironna M, Germinario C, Lopalco PL, Carrozzini F, Barbuti S, Quarto M. Prevalence rates of viral hepatitis infections in refugee Kurds from Iraq and Turkey. Infection. 2003;31(2):70-4.

286. Chironna M, Germinario C, Lupalco PL, Carrozzini F, Quarto M. Prevalence of hepatitis virus infections in Kosovar refugees. International Journal of Infectious Diseases. 2001;5(4):209-13.

287. Dalekos GN, Zervou E, Elisaf M, Germanos N, Galanakis E, Bourantas K, et al. Antibodies to hepatitis E virus among several populations in Greece: increased prevalence in an hemodialysis unit. Transfusion. 1998;38(6):589-95.

288. Jablonka A, Solbach P, Wöbse M, Manns MP, Schmidt RE, Wedemeyer H, et al. Seroprevalence of antibodies and antigens against hepatitis A-E viruses in refugees and asylum seekers in Germany in 2015. European Journal of Gastroenterology and Hepatology. 2017;29(8):939-45.

289. Lagare A, Ibrahim A, Ousmane S, Issaka B, Zaneidou M, Kadadé G, et al. Outbreak of Hepatitis E virus infection in displaced persons camps in Diffa region, Niger, 2017. Am J Trop Med Hyg. 2018;99(4):1055-7.

290. Malamitsi-Puchner A, Papacharitonos S, Sotos D, Tzala L, Psichogiou M, Hatzakis A, et al. Prevalence study of different hepatitis markers among pregnant Albanian refugees in Greece. European Journal of Epidemiology. 1996;12(3):297-301.

291. Mérens A, Guérin PJ, Guthmann JP, Nic, E. Outbreak of hepatitis E virus infection in Darfur, Sudan: Effectiveness of real-time reverse transcription-PCR analysis of dried blood spots. J Clin Microbiol. 2009;47(6):1931-3.

292. Moaven L, Van Asten M, Crofts N, Locarnini SA. Seroepidemiology of hepatitis E in selected Australian populations. J Med Virol. 1995;45(3):326-30.

293. Nicand E, Armstrong GL, Enouf V, Guthmann JP, Guerin JP, Caron M, et al. Genetic heterogeneity of hepatitis E virus in Darfur, Sudan, and neighboring Chad. Journal of Medical Virology. 2005;77(4):519-21.

294. Sencan I, Sahin I, Kaya D, Oksuz S, Yildirim M. Assessment of HAV and HEV seroprevalence in children living in post-earthquake camps from Düzce, Turkey. Eur J Epidemiol. 2004;19(5):461-5.

295. Thomson K, Luis Dvorzak J, Lagu J, Laku R, Dineen B, Schilperoord M, et al. Investigation of hepatitis E outbreak among refugees - Upper Nile, South Sudan, 2012-2013. MMWR. 2013;62(29):581-6.

296. Ahmed A, Ali Y, Siddig EE, Hamed J, Mohamed NS, Khairy A, et al. Hepatitis E virus outbreak among Tigray war refugees from Ethiopia, Sudan. Emerg Infect Dis. 2022;28(8):1722-4. doi:10.3201/eid2808.220397.

297. da Costa e Silva GR, Martins TLS, de Almeida Silva C, Caetano KAA, dos Santos Carneiro MA, Silva BVDE, et al. Hepatitis A and E among immigrants and refugees in Central Brazil. Revista de Saude Publica. 2022;56. doi:10.11606/S1518-8787.2022056003839.

298. Desai AN, Mohareb AM, Elkarsany MM, Desalegn H, Madoff LC, Lassmann B. Viral hepatitis E outbreaks in refugees and internally displaced populations, sub-Saharan Africa, 2010–2020. Emerg Infect Dis. 2022;28(5):1074-6. doi:10.3201/eid2805.212546.

299. Teshale EH, Grytdal SP, Howard C, Barry V, Kamili S, Drobeniuc J, et al. Evidence of person-to-person transmission of hepatitis E virus during a large outbreak in Northern Uganda. Clinical Infectious Diseases. 2010;50(7):1006-10. doi:10.1086/651077.

300. Isaäcson M, Frean J, He J, Seriwatana J, Innis BL. An outbreak of hepatitis E in Northern Namibia, 1983. Am J Trop Med Hyg. 2000;62(5):619-25.

301. Guthmann JP, Klovstad H, Boccia D, Hamid N, Pinoges L, Nizou JY, et al. A large outbreak of hepatitis E among a displaced population in Darfur, Sudan, 2004: the role of water treatment methods. Clin Infect Dis. 2006;42(12):1685-91. doi: 10.1086/504321.

302. Botros BAM, Watts DM, Soliman AK, Salib AW, Moussa MI, Mursal H, et al. Serological evidence of dengue fever among refugees, Hargeysa, Somalia. J Med Virol. 1989;29(2):79-81.

303. Yildirim C, Arda B, Uz I, Uyar M, Ersel M, Yamazhan T, et al. Wars do not kill only with guns: A case of rabies in a Syrian refugee. Mediterranean Journal of Infection Microbes and Antimicrobials. 2017;6:2.

304. Baggett TP, Racine MW, Lewis E, De Las Nueces D, O’Connell JJ, Bock B, et al. Addressing COVID-19 among people experiencing homelessness: Description, adaptation, and early findings of a multiagency response in Boston. Public Health Rep. 2020;135(4):435-41.

305. Heudorf U, Steul K, Gottschalk R. Sars-Cov-2 in children - insights and conclusions from the mandatory reporting data in Frankfurt am Main, Germany, March-July 2020. Gms Hygiene and Infection Control. 2020;15:12.

306. Le Bihan C, Faucherre V, Le Moing V, Mehenni A, Nantes D, Da Silva A, et al. COVID-19: The forgotten cases of hidden exiles. Infect Dis Now. 2021.

307. Ly TDA, Nguyen NN, Hoang VT, Goumballa N, Louni M, Canard N, et al. Screening of SARS-CoV-2 among homeless people, asylum-seekers and other people living in precarious conditions in Marseille, France, March–April 2020. International Journal of Infectious Diseases. 2021;105:1-6.

308. Ralli M, Cedola C, Urbano S, Latini O, Shkodina N, Morrone A, et al. Assessment of SARS-CoV-2 infection through rapid serology testing in the homeless population in the City of Rome, Italy. Preliminary results. J Public Health Res. 2020;9(4):556-9.

309. Redditt V, Wright V, Rashid M, Male R, Bogoch I. Outbreak of SARS-CoV-2 infection at a large refugee shelter in Toronto, April 2020: a clinical and epidemiologic descriptive analysis. CMAJ Open. 2020;8(4):E819-e24.

310. Turunen T, Kontunen K, Sugulle K, Hieta P, Snellman O, Hussein I, et al. COVID-19 outbreak at a reception centre for asylum seekers in Espoo, Finland. J Migr Health. 2021;3:100043.

311. Al-Hatamleh MAI, Hatmal MM, Mustafa SHF, Alzu'bi M, AlSou'b AF, Abughanam SNS, et al. Experiences and perceptions of COVID-19 infection and vaccination among Palestinian refugees in Jerash camp and Jordanian citizens: a comparative cross-sectional study by face-to-face interviews. Infectious diseases of poverty. 2022;11(1):123. doi:10.1186/s40249-022-01047-y.

312. Altare C, Kostandova N, Okeeffe J, Hayek H, Fawad M, Musa Khalifa A, et al. COVID-19 epidemiology and changes in health service utilization in Azraq and Zaatari refugee camps in Jordan: A retrospective cohort study. PLoS Medicine. 2022;19(5). doi:10.1371/journal.pmed.1003993.

313. Altare C, Kostandova N, Okeeffe J, Omwony E, Nyakoojo R, Kasozi J, et al. COVID-19 epidemiology and changes in health service utilization in Uganda’s refugee settlements during the first year of the pandemic. BMC Public Health. 2022;22(1):1927. doi: 10.1186/s12889-022-14305-3.

314. Baggio S, Jacquerioz F, Salamun J, Spechbach H, Jackson Y. Equity in access to COVID-19 testing for undocumented migrants and homeless persons during the initial phase of the pandemic. Journal of Migration and Health. 2021;4. doi:10.1016/j.jmh.2021.100051.

315. Balakrishnan VS. Impact of COVID-19 on migrants and refugees. The Lancet Infectious diseases. 2021;21(8):1076-7. doi:10.1016/S1473-3099(21)00410-2.

316. Bojorquez-Chapela I, Strathdee SA, Garfein RS, Benson CA, Chaillon A, Ignacio C, et al. The impact of the COVID-19 pandemic among migrants in shelters in Tijuana, Baja California, Mexico. BMJ Global Health. 2022;7(3). doi:10.1136/bmjgh-2021-007202.

317. da Silva HP, Abreu IN, Lima CNC, de Lima ACR, Barbosa AD, de Oliveira LR, et al. Migration in times of pandemic: SARS-CoV-2 infection among the Warao indigenous refugees in Belem, Para, Amazonia, Brazil. BMC Public Health. 2021;21(1). doi:10.1186/s12889-021-11696-7.

318. Dressler A, Finci I, Wagner-Wiening C, Eichner M, Brockmann SO. Epidemiological analysis of 3,219 COVID-19 outbreaks in the state of Baden-Wuerttemberg, Germany. Epidemiology and Infection. 2021. doi:10.1017/S0950268821000911.

319. Fabris S, d'Ettorre G, Spagnolello O, Russo A, Lopalco M, D'Agostino F, et al. SARS-CoV-2 among migrants recently arrived in Europe from low- and middle-income countries: Containment strategies and special features of management in reception centers. Frontiers in Public Health. 2021;9. doi:10.3389/fpubh.2021.735601.

320. Gignoux E, Athanassiadis F, Yarrow AG, Jimale A, Mubuto N, Déglise C, et al. Seroprevalence of SARS-CoV-2 antibodies and retrospective mortality in a refugee camp, Dagahaley, Kenya. PLoS One. 2021;16(12). doi:10.1371/journal.pone.0260989.

321. Hongsermeier-Graves N, Khazanchi R, Marcelin JR, Fadul N. Structural vulnerability among patients with HIV and SARS-CoV-2 Co-infection: descriptive case series from the U.S. Midwest. AIDS Care - Psychological and Socio-Medical Aspects of AIDS/HIV. 2022;34(11):1372-7. doi:10.1080/09540121.2021.1981224.

322. Johnson-Agbakwu CE, Eakin CM, Bailey CV, Sood S, Ali N, Doehrman P, et al. Severe acute respiratory syndrome coronavirus 2: a canary in the coal mine for public safety net hospitals. AJOG Global Reports. 2021;1(2). doi:10.1016/j.xagr.2021.100009.

323. Kalani N, Hatami N, Haghbeen M, Yaqoob U, Raeyat Doost E. Covid-19 health care for afghan refugees as a minor ethnicity in iran; clinical differences and racial equality in health. Acta Medica Iranica. 2021;59(8):466-71.

324. Kheirallah KA, Ababneh BF, Bendak H, Alsuwaidi AR, Elbarazi I. Exploring the mental, social, and lifestyle effects of a positive COVID-19 infection on Syrian refugees in Jordan: A qualitative study. International Journal of Environmental Research and Public Health. 2022;19(19). doi:10.3390/ijerph191912588.

325. Knust B, Wongjindanon N, Moe AA, Herath L, Kaloy W, Soe TT, et al. Enhancing respiratory disease surveillance to detect COVID-19 in shelters for displaced persons, Thailand-Myanmar Border, 2020-2021. Emerg Infect Dis. 2022;28(13):S17-s25. doi: 10.3201/eid2813.220324.

326. Kondilis E, Papamichail D, McCann S, Carruthers E, Veizis A, Orcutt M, et al. The impact of the COVID-19 pandemic on refugees and asylum seekers in Greece: A retrospective analysis of national surveillance data from 2020. EClinicalMedicine. 2021;37. doi:10.1016/j.eclinm.2021.100958.

327. Mellou K, Gkolfinopoulou K, Andreopoulou A, Tsekou A, Papadima K, Stamoulis K, et al. A COVID-19 outbreak among migrants in a hosting facility in Greece, April 2020. Journal of Infection Prevention. 2022;23(5):235-8. doi:10.1177/17571774221092568.

328. Palacios CF, Tucker EW, Travassos MA. Coronavirus disease 2019 burden among unaccompanied minors in US custody. Clinical Infectious Diseases. 2022. doi:10.1093/cid/ciac636.

329. Pham PN, Keegan K, Johnston LG, Rodas J, Restrepo MA, Wei C, et al. Assessing the impact of the COVID-19 pandemic among Venezuelan refugees and migrants in Colombia using respondent-driven sampling (RDS). BMJ Open. 2022;12(10). doi:10.1136/bmjopen-2021-054820.

330. Prodanuk M, Wagner S, Orkin J, Noone D. Social vulnerability and COVID-19: A call to action for paediatric clinicians. Paediatrics and Child Health (Canada). 2021;26(1):1-3. doi:10.1093/pch/pxaa121.

331. Sharov KS. SARS-CoV-2 spread in different biosocial strata in Russia in 2020: Groups of risk and victimised groups. Journal of Global Health. 2021;11. doi: 10.7189/jogh.11.03066.

332. Sisti LG, Di Napoli A, Petrelli A, Rossi A, Diodati A, Menghini M, et al. Covid-19 impact in the italian reception system for migrants during the nationwide lockdown: A national observational study. International Journal of Environmental Research and Public Health. 2021;18(23). doi:10.3390/ijerph182312380.

333. Tambuzzi S, Cummaudo M, Maggioni L, Tritella S, Lucchesi B, Montedoro P, et al. A pilot COVID-19 surveillance program at the Zendrini center in Milan (Italy) for unaccompanied foreign minors. Children (Basel). 2022;9(10). doi:10.3390/children9101485.

334. Tolunay O, Çelik Ü, Arslan I, Tutun B, Özkaya M. Evaluation of clinical findings and treatment results of Coronavirus disease 2019 (COVID-19) in pediatric cancer patients: A single center experience. Frontiers in Pediatrics. 2022;10. doi:10.3389/fped.2022.848379.

335. Vallejo-Janeta AP, Morales-Jadan D, Freire-Paspuel B, Lozada T, Cherrez-Bohorquez C, Garcia-Bereguiain MA, et al. COVID-19 outbreaks at shelters for women who are victims of gender-based violence from Ecuador. International Journal of Infectious Diseases. 2021;108:531-6. doi:10.1016/j.ijid.2021.06.012.

336. Wamala JF, Loro F, Deng SJ, Berta KK, Guyo AG, Mpairwe A, et al. Epidemiological characterization of COVID-19 in displaced populations of South Sudan. Pan African Medical Journal. 2022;41(2). doi:10.11604/pamj.supp.2022.42.1.33767.

337. Yan XY, Xiao W, Zhou SP, Wang XC, Wang ZK, Zhao MC, et al. A four-generation family transmission chain of COVID-19 along the China-Myanmar border in October to November 2021. Frontiers in Public Health. 2022;10. doi:10.3389/fpubh.2022.1004817.

338. Zhang M, Gurung A, Anglewicz P, Baniya K, Yun K. Discrimination and stress among Asian refugee populations during the COVID-19 pandemic: Evidence from Bhutanese and Burmese refugees in the USA. Journal of Racial and Ethnic Health Disparities. 2022;9(2):589-97. doi:10.1007/s40615-021-00992-y.

339. Khan S, Akbar SMF, Kimitsuki K, Saito N, Yahiro T, Al Mahtab M, et al. Recent downhill course of COVID-19 at Rohingya refugee camps in Bangladesh: Urgent action solicited. J Glob Health. 2021;11:03097. doi: 10.7189/jogh.11.03097.

340. Rodríguez-Morales AJ, Bonilla-Aldana DK, Bonilla-Aldana JC, Mondragón-Cardona Á. Arboviral diseases among internally displaced people of Neiva, Colombia, 2015-2017. Travel Med Infect Dis. 2019;26(2).

341. Ahmed A, Elduma A, Magboul B, Higazi T, Ali Y. The first outbreak of dengue fever in Greater Darfur, Western Sudan. Trop Med Infect Dis. 2019;4(1).

342. Nyakarahuka L, Whitmer S, Kyondo J, Mulei S, Cossaboom CM, Telford CT, et al. Crimean-Congo hemorrhagic fever outbreak in refugee settlement during COVID-19 pandemic, Uganda, April 2021. Emerg Infect Dis. 2022;28(11):2326-9. doi:10.3201/eid2811.220365.

343. Rahim M, Kazi BM, Bile KM, Munir M, Khan AR. The impact of the disease early warning system in responding to natural disasters and conflict crises in Pakistan. Eastern Mediterranean Health Journal. 2010;16:S114-21. doi:10.26719/2010.16.supp.114.

344. Ahmed A, Eldigail M, Elduma A, Breima T, Dietrich I, Ali Y, et al. First report of epidemic dengue fever and malaria co-infections among internally displaced persons in humanitarian camps of North Darfur, Sudan. International Journal of Infectious Diseases. 2021;108:513-6. doi:10.1016/j.ijid.2021.05.052.

345. Alawa J, Al-Ali S, Walz L, Wiles E, Harle N, Awale MA, et al. Knowledge and perceptions of COVID-19, prevalence of pre-existing conditions and access to essential resources in Somali IDP camps: a cross-sectional study. BMJ Open. 2021;11(6). doi:10.1136/bmjopen-2020-044411.

346. Espinel CH. A piece of my mind. On the trail of color. JAMA. 1996;275(3):168.

347. Chen L, Peek M, Stokich D, Todd R, Anderson M, Murphy FK, et al. Japanese encephalitis in two children-United States, 2010. MMWR. 2011;60(9):276-8.
