# Supplementary material for: Status of zoonotic disease research in refugees, asylum seekers and internally displaced people, globally: A scoping review of forty clinically important zoonotic pathogens
Source: PLoS Negl Trop Dis. 2024 May 20;18(5):e0012164. doi: 10.1371/journal.pntd.0012164 (PMC11142688; doi:10.1371/journal.pntd.0012164)
Supplement: S2 Table — (DOCX) [file pntd.0012164.s004.docx]

**S2 Table: Publications included in the scoping review reporting negative results**

| Pathogen | References |
| --- | --- |
| *Schistosoma* spp. | [1-8] |
| *Filarioidea* | [9-11] |
| *G. lamblia* | [12] |
| *Leptospira* spp. | [13] |
| *Salmonella* *enterica* subsp. *enterica* serovar typhi | [14] |
| *Echinococcus* spp. | [3] |
| SARS-CoV-2 | [15] |
| Hepatitis E virus | [16] |
| Middle East respiratory syndrome coronavirus | [17] |
| Zika virus | [18] |
| Chikungunya virus | [18] |
| Crimean-Congo haemorrhagic fever virus | [18] |
| Rift Valley fever virus | [18] |
| Yellow Fever virus | [18] |

**References**

1. Buonfrate D, Gobbi F, Marchese V, Postiglione C, Monteiro GB, Giorli G, et al. Extended screening for infectious diseases among newly-arrived asylum seekers from Africa and Asia, Verona province, Italy, April 2014 to June 2015. Eurosurveillance. 2019;23(16):7-14.

2. Varkey P, Jerath AU, Bagniewski S, Lesnick T. Intestinal parasitic infection among new refugees to Minnesota, 1996-2001. Travel Med Infect Dis. 2007;5(4):223-9.

3. Zöllkau J, Ankert J, Pletz MW, Mishra S, Seliger G, Lobmaier SM, et al. Hepatitis E, schistosomiasis and echinococcosis–Prevalence in a cohort of pregnant migrants in Germany and their influence on fetal growth restriction. Pathogens. 2022;11(1). doi:10.3390/pathogens11010058.

4. Harkensee C, Andrew R. Health needs of accompanied refugee and asylum-seeking children in a UK specialist clinic. Acta Paediatr. 2021;110(8):2396-404. doi: 10.1111/apa.15861.

5. Bergevin A, Husain M, Cruz M, Blanc CL, Dieme A, Girardin ML, et al. Medical check-up of newly arrived unaccompanied minors: A dedicated pediatric consultation service in a hospital. Archives de Pediatrie. 2021;28(8):689-95. doi:10.1016/j.arcped.2021.09.012.

6. Williams B, Boullier M, Cricks Z, Ward A, Naidoo R, Williams A, et al. Screening for infection in unaccompanied asylum-seeking children and young people. Archives of Disease in Childhood. 2020;105(6):530-2.

7. Ehlkes L, George M, Knautz D, Burckhardt F, Jahn K, Vogt M, et al. Negligible import of enteric pathogens by newly-arrived asylum seekers and no impact on incidence of notified Salmonella and Shigella infections and outbreaks in Rhineland-Palatinate, Germany, January 2015 to May 2016. Euro Surveill. 2018;23(20):7-14.

8. Chang AH, Perry S, Du JNT, Agunbiade A, Polesky A, Parsonnet J. Decreasing intestinal parasites in recent northern California refugees. Am J Trop Med Hyg. 2013;88(1):191-7.

9. Brodine SK, Thomas A, Huang R, Harbertson J, Mehta S, Leake J, et al. Community based parasitic screening and treatment of sudanese refugees: Application and assessment of centers for disease control guidelines. Am J Trop Med Hyg. 2009;80(3):425-30.

10. Montour J, Lee D, Snider C, Jentes ES, Stauffer W. Absence of Loa loa microfilaremia among newly arrived congolese refugees in Texas. Am J Trop Med Hyg. 2017;97(6):1833-5.

11. Yangco BG, Vincent AL, Vickery AC. A survey of filariasis among refugees in South Florida. Am J Trop Med Hyg. 1984;33(2):246-51.

12. Caruana SR, Kelly HA, Ngeow JYY, Ryan NJ, Bennett CM, Chea L, et al. Undiagnosed and potentially lethal parasite infections among immigrants and refugees in Australia. Journal of Travel Medicine. 2006;13(4):233-9.

13. Lin CY, Chen TC, Dai CY, Yu ML, Lu PL, Yen JH, et al. Serological investigation to identify risk factors for post-flood infectious diseases: a longitudinal survey among people displaced by Typhoon Morakot in Taiwan. BMJ Open. 2015;5(5):e007008.

14. Van Boetzelaer E, Fotso A, Angelova I, Huisman G, Thorson T, Hadj-Sahraoui H, et al. Health conditions of migrants, refugees and asylum seekers on search and rescue vessels on the central Mediterranean Sea, 2016-2019: A retrospective analysis. BMJ Open. 2022;12(1). doi:10.1136/bmjopen-2021-053661.

15. Tambuzzi S, Cummaudo M, Maggioni L, Tritella S, Lucchesi B, Montedoro P, et al. A pilot COVID-19 surveillance program at the Zendrini center in Milan (Italy) for unaccompanied foreign minors. Children (Basel). 2022;9(10). doi:10.3390/children9101485.

16. Bliss J, Bouhenia M, Hale P, Couturier BA, Iyer AS, Rumunu J, et al. High prevalence of shigella or enteroinvasive Escherichia coli carriage among residents of an internally displaced persons camp in South Sudan. Am J Trop Med Hyg. 2018;98(2):595-7.

17. Aliskin O, Savas N. Notifiable communicable diseases in Turkey and their notification status: Antakya sample. Flora the Journal of Infectious Diseases and Clinical Microbiology. 2019;24(1):11-21.

18. Botros BAM, Watts DM, Soliman AK, Salib AW, Moussa MI, Mursal H, et al. Serological evidence of dengue fever among refugees, Hargeysa, Somalia. J Med Virol. 1989;29(2):79-81.
