# Supplementary material for: Status of zoonotic disease research in refugees, asylum seekers and internally displaced people, globally: A scoping review of forty clinically important zoonotic pathogens
Source: PLoS Negl Trop Dis. 2024 May 20;18(5):e0012164. doi: 10.1371/journal.pntd.0012164 (PMC11142688; doi:10.1371/journal.pntd.0012164)
Supplement: S5 Table — (DOCX) [file pntd.0012164.s007.docx]

**S5 Table: Publications included in the scoping review reporting on displaced people in camp/reception centre, community or mixed settings**

| Setting | References |
| --- | --- |
| Camp/ Reception centre | [1-148] |
| Community | [149-219] |
| Mixed | [220-240] |

**References**

1. Hepatitis E, Chad. Weekly epidemiological record / Health Section of the Secretariat of the League of Nations. 2004;79(35):313.

2. Haq KUA, Gul NA, Hammad HM, Bibi Y, Bibi A, Mohsan J. Prevalence of Giardia intestinalis and Hymenolepis nana in Afghan refugee population of Mianwali district, Pakistan. Afr Health Sci. 2015;15(2):394-400.

3. Abu Mourad TA. Palestinian refugee conditions associated with intestinal parasites and diarrhoea: Nuseirat refugee camp as a case study. Public Health. 2004;118(2):131-42.

4. Abu-Alrub SM, Abusada GM, Farraj MA, Essawi TA. Prevalence of Cryptosporidium spp. in children with diarrhoea in the West Bank, Palestine. J Infect Dev Ctries. 2008;2(1):59-62.

5. Ackermann N, Marosevic D, Hörmansdorfer S, Eberle U, Rieder G, Treis B, et al. Screening for infectious diseases among newly arrived asylum seekers, Bavaria, Germany, 2015. Eurosurveillance. 2018;23(10).

6. Addams J, Lainhart W. The Brief Case: Salmonella enterica Serovar Typhi in a Central American Refugee. Clin Microbiol Infect. 2021;59(5).

7. Agha Rodina AI, Teoderescu I. Prevalence of intestinal parasites in three localities in Gaza Governorates - Palestine. Archives of Public Health. 2002;60(6):363-70.

8. Ahmed A, Ali Y, Siddig EE, Hamed J, Mohamed NS, Khairy A, et al. Hepatitis E virus outbreak among Tigray war refugees from Ethiopia, Sudan. Emerg Infect Dis. 2022;28(8):1722-4. doi:10.3201/eid2808.220397.

9. Ahmed A, Eldigail M, Elduma A, Breima T, Dietrich I, Ali Y, et al. First report of epidemic dengue fever and malaria co-infections among internally displaced persons in humanitarian camps of North Darfur, Sudan. International Journal of Infectious Diseases. 2021;108:513-6. doi:10.1016/j.ijid.2021.05.052.

10. Ahmed A, Elduma A, Magboul B, Higazi T, Ali Y. The first outbreak of dengue fever in Greater Darfur, Western Sudan. International Journal of Infectious Diseases. 2019;4(1).

11. Ahmed JA, Moturi E, Spiegel P, Schilperoord M, Burton W, Kassim NH, et al. Hepatitis E outbreak, Dadaab refugee camp, Kenya, 2012. Emerg Infect Dis. 2013;19(6):1010-2.

12. Ahmed T, Maheswary NP, Khan NI. Filariasis in Mirpur area of Dhaka city. Bangladesh Med Res Counc Bull. 1986;12(2):83-94.

13. Al-Halaweh AA, Almdal T, O'Rourke N, Davidovitch N. Mobile care teams improve metabolic control for adults with Type II diabetes in the Southern West Bank, Palestine. Diabetes Metab Syndr. 2019;13(1):782-5. doi: 10.1016/j.dsx.2018.11.066.

14. Al-Hindi AI, Abu Shammala BM. Dientamoeba fragilis in gaza strip: A neglected protozoan parasite. Iran J Parasitol. 2013;8(2):249-55.

15. Al-Jawabreh A, Barghuthy F, Schnur LF, Jacobson RL, Schönian G, Abdeen Z. Epidemiology of cutaneous leishmaniasis in the endemic area of Jericho, Palestine. East Mediterr Health J. 2003;9(4):805-15.

16. Alawa J, Al-Ali S, Walz L, Wiles E, Harle N, Awale MA, et al. Knowledge and perceptions of COVID-19, prevalence of pre-existing conditions and access to essential resources in Somali IDP camps: a cross-sectional study. BMJ Open. 2021;11(6). doi:10.1136/bmjopen-2020-044411.

17. Altare C, Kostandova N, Okeeffe J, Hayek H, Fawad M, Musa Khalifa A, et al. COVID-19 epidemiology and changes in health service utilization in Azraq and Zaatari refugee camps in Jordan: A retrospective cohort study. PLoS Medicine. 2022;19(5). doi:10.1371/journal.pmed.1003993.

18. Altare C, Kostandova N, Okeeffe J, Omwony E, Nyakoojo R, Kasozi J, et al. COVID-19 epidemiology and changes in health service utilization in Uganda’s refugee settlements during the first year of the pandemic. BMC Public Health. 2022;22(1):1927. doi: 10.1186/s12889-022-14305-3.

19. Antinori S, Tonello C, Edouard S, Parravicini C, Gastaldi D, Gr, et al. Diagnosis of louse-borne relapsing fever despite negative microscopy in two asylum seekers from Eastern Africa. Am J Trop Med Hyg. 2017;97(6):1669-72.

20. Arthur JD, Bodhidatta L, Echeverria P, Phuphaisan S, Paul S. Diarrheal disease in Cambodian children at a camp in Thailand. Am J Epidemiol. 1992;135(5):541-51.

21. Azman AS, Bouhenia M, Iyer AS, Rumunu J, Laku RL, Wamala JF, et al. High hepatitis E seroprevalence among displaced persons in South Sudan. Am J Trop Med Hyg. 2017;96(6):1296-301.

22. Baggett TP, Racine MW, Lewis E, De Las Nueces D, O’Connell JJ, Bock B, et al. Addressing COVID-19 among people experiencing homelessness: Description, adaptation, and early findings of a multiagency response in Boston. Public Health Rep. 2020;135(4):435-41.

23. Balakrishnan VS. Impact of COVID-19 on migrants and refugees. The Lancet Infectious diseases. 2021;21(8):1076-7. doi:10.1016/S1473-3099(21)00410-2.

24. Benzeguir AK, Capraru T, Aust-Kettis A, Björkman A. High frequency of gastrointestinal parasites in refugees and asylum seekers upon arrival in Sweden. Scand J Infect Dis. 1999;31(1):79-82.

25. Binga WE, Houmsou RS, Garba LC, Amuta EU, Suntaya KL. Use of rivers' water, inadequate hygiene, and sanitation as exposure of internally displaced persons (IDPs) to urogenital schistosomiasis and soil-transmitted helminthiasis in Jalingo Local Government Area (LGA), Taraba State, Nigeria. Journal of Water Sanitation and Hygiene for Development. 2022. doi:10.2166/washdev.2022.089.

26. Bliss J, Bouhenia M, Hale P, Couturier BA, Iyer AS, Rumunu J, et al. High prevalence of shigella or enteroinvasive Escherichia coli carriage among residents of an internally displaced persons camp in South Sudan. Am J Trop Med Hyg. 2018;98(2):595-7.

27. Bloch-Infanger C, Bättig V, Kremo J, Widmer AF, Egli A, Bingisser R, et al. Increasing prevalence of infectious diseases in asylum seekers at a tertiary care hospital in Switzerland. PLoS One. 2017;12(6):e0179537.

28. Boccia D, Guthmann JP, Klovstad H, Hamid N, Tatay M, Ciglenecki I, et al. High mortality associated with an outbreak of hepatitis E among displaced persons in Darfur, Sudan. Clinical Infectious Diseases. 2006;42(12):1679-84.

29. Bojorquez-Chapela I, Strathdee SA, Garfein RS, Benson CA, Chaillon A, Ignacio C, et al. The impact of the COVID-19 pandemic among migrants in shelters in Tijuana, Baja California, Mexico. BMJ Global Health. 2022;7(3). doi:10.1136/bmjgh-2021-007202.

30. Botros BAM, Watts DM, Soliman AK, Salib AW, Moussa MI, Mursal H, et al. Serological evidence of dengue fever among refugees, Hargeysa, Somalia. J Med Virol. 1989;29(2):79-81.

31. Brooker S, Mohammed N, Adil K, Agha S, Reithinger R, Rowl, et al. Leishmaniasis in refugee and local Pakistani populations. Emerg Infect Dis. 2004;10(9):1681-4.

32. Brown AE, Meek SR, Maneechai N, Lewis GE. Murine typhus among Khmers living at an evacuation site on the Thai-Kampuchean border. Am J Trop Med Hyg. 1988;38(1):168-71.

33. Brown V, Larouze B, Desve G, Rousset JJ, Thibon M, Fourrier A, et al. Clinical presentation of louse-born relapsing fever among Ethiopian refugees in northern Somalia. Ann Trop Med Parasitol 1988;82(5):499-502.

34. Browne LB, Menkir Z, Kahi V, Maina G, Asnakew S, Tubman M, et al. Hepatitis E outbreak among refugees from South Sudan - Gambella, Ethiopia, April 2014-January 2015. MMWR. 2015;64(19):537.

35. Buonfrate D, Gobbi F, Marchese V, Postiglione C, Monteiro GB, Giorli G, et al. Extended screening for infectious diseases among newly-arrived asylum seekers from Africa and Asia, Verona province, Italy, April 2014 to June 2015. Eurosurveillance. 2019;23(16):7-14.

36. Carreras-Abad C, Oliveira-Souto I, Pou-Ciruelo D, Pujol-Morro JM, Soler-Palacín P, Soriano-Arandes A, et al. Health and vaccination status of unaccompanied minors after arrival in a European border country: A cross-sectional study (2017-2020). Pediatric Infectious Disease Journal. 2022;41(11):872-7. doi:10.1097/INF.0000000000003670.

37. Chalupa P, Vanista J, Burget I, Stary J, Sukova M, Nohynkova M. The review of imported visceral leishmaniosis in the Czech Republic. Bratislavské lekárske listy. 2001;102(2):84-91.

38. Chandrasena TGAN, Hapuarachchi HC, Dayanath MYD, Pathmeswaran A, De Silva NR. Intestinal parasites and the growth status of internally displaced children in Sri Lanka. Trop Doct. 2007;37(3):163-5.

39. Chen L, Peek M, Stokich D, Todd R, Anderson M, Murphy FK, et al. Japanese encephalitis in two children-United States, 2010. MMWR. 2011;60(9):276-8.

40. Cherian P, Junckerstorff RK, Rosen D, Kumarasinghe P, Morling A, Tuch P, et al. Late-stage human African trypanosomiasis in a Sudanese refugee. Medical Journal of Australia. 2010;192(7):417-9.

41. Chernet A, Kling K, Sydow V, Kuenzli E, Hatz C, Utzinger J, et al. Accuracy of diagnostic tests for Schistosoma mansoni infection in asymptomatic Eritrean refugees: Serology and point-of-care circulating cathodic antigen against stool microscopy. Clinical Infectious Diseases. 2017;65(4):568-74.

42. Chironna M, Germinario C, Lopalco PL, Carrozzini F, Barbuti S, Quarto M. Prevalence rates of viral hepatitis infections in refugee Kurds from Iraq and Turkey. Infection. 2003;31(2):70-4.

43. Chironna M, Germinario C, Lupalco PL, Carrozzini F, Quarto M. Prevalence of hepatitis virus infections in Kosovar refugees. International Journal of Infectious Diseases. 2001;5(4):209-13.

44. Cortier M, de La Porte C, Papot E, Goudjo A, Guenneau L, Riou F, et al. Health status and healthcare trajectory of vulnerable asylum seekers hosted in a French Reception Center. Travel Medicine and Infectious Disease. 2022;46. doi:10.1016/j.tmaid.2021.102180.

45. Costescu Strachinaru DI, Cambier J, et-Yattara H, Konopnicki D. Relapsing fever in asylum seekers from Somalia arriving in Belgium in August 2015. Acta Clin Belg. 2016;71(5):353-5.

46. D'Alauro F, Lee RV, Pao-In K, Khairallah M. Intestinal parasites and pregnancy. Infect Dis Obstet Gynecol. 1985;66(5):639-43.

47. Dalekos GN, Zervou E, Elisaf M, Germanos N, Galanakis E, Bourantas K, et al. Antibodies to hepatitis E virus among several populations in Greece: increased prevalence in an hemodialysis unit. Transfusion. 1998;38(6):589-95.

48. de Beer P, el Harith A, van Grootheest M, Winkler A. Outbreak of kala-azar in the Sudan. Lancet. 1990;335(8683):224. doi: 10.1016/0140-6736(90)90313-t.

49. Desai AN, Mohareb AM, Elkarsany MM, Desalegn H, Madoff LC, Lassmann B. Viral Hepatitis E outbreaks in refugees and internally displaced populations, sub-Saharan Africa, 2010–2020. Emerg Infect Dis. 2022;28(5):1074-6. doi:10.3201/eid2805.212546.

50. Duffy PE, Le Guillouzic H, Gass RF, Innis BL. Murine typhus identified as a major cause of febrile illness in a camp for displaced Khmers in Thailand. Am J Trop Med Hyg. 1990;43(5):520-6.

51. Edosomwan EU, Evbuomwan IO, Agbalalah C, Dahunsi SO, Abhulimhen-Iyoha BI. Malaria coinfection with Neglected Tropical Diseases (NTDs) in children at Internally Displaced Persons (IDP) camp in Benin City, Nigeria. Heliyon. 2020;6(8).

52. Ehlkes L, George M, Knautz D, Burckhardt F, Jahn K, Vogt M, et al. Negligible import of enteric pathogens by newly-arrived asylum seekers and no impact on incidence of notified Salmonella and Shigella infections and outbreaks in Rhineland-Palatinate, Germany, January 2015 to May 2016. Euro Surveill. 2018;23(20):7-14.

53. Eksi F, Ozgoztasi O, Karsligil T, Saglam M. Genotyping Leishmania promastigotes isolated from patients with cutaneous leishmaniasis in south-eastern Turkey. Journal of International Medical Research. 2016;45(1):114-22.

54. Enabulele EE, Platt RN, Adeyemi E, Agbosua E, Aisien MSO, Ajakaye OG, et al. Urogenital schistosomiasis in Nigeria post receipt of the largest single praziquantel donation in Africa. Acta Tropica. 2021:105916.

55. Evbuomwan IO, Edosomwan EU, Idubor V, Bazuaye C, Abhulimhen-Iyoha BI, Adeyemi OS, et al. Survey of intestinal parasitism among schoolchildren in internally displaced persons camp, Benin City, Nigeria. Scientific African. 2022;17. doi:10.1016/j.sciaf.2022.e01373.

56. Fabris S, d'Ettorre G, Spagnolello O, Russo A, Lopalco M, D'Agostino F, et al. SARS-CoV-2 among migrants recently arrived in Europe from low- and middle-income countries: Containment strategies and special features of management in reception centers. Frontiers in Public Health. 2021;9. doi:10.3389/fpubh.2021.735601.

57. Fritzsche M, Gottstein B, Wigglesworth MC, Eckert J. Serological survey of human cysticercosis in Irianese refugee camps in Papua New Guinea. Acta Tropica. 1990;47(2):69-77.

58. Gignoux E, Athanassiadis F, Yarrow AG, Jimale A, Mubuto N, Déglise C, et al. Seroprevalence of SARS-CoV-2 antibodies and retrospective mortality in a refugee camp, Dagahaley, Kenya. PLoS One. 2021;16(12). doi:10.1371/journal.pone.0260989.

59. Goldenberger D, Claas GJ, Bloch-Infanger C, Breidthardt T, Suter B, Martinez M, et al. Louse-borne relapsing fever (Borrelia recurrentis) in an Eritrean refugee arriving in Switzerland, August 2015. Euro Surveill. 2015;20(32):2-5.

60. Gozalbo M, Guillen M, Taroncher-Ferrer S, Cifre S, Carmena D, Soriano JM, et al. Assessment of the nutritional status, diet and intestinal parasites in hosted Saharawi children. Children (Basel). 2020;7(12):18.

61. Gray GC, Rodier GR, Matras-Maslin VC, Honein MA, Ismail EA, Botros BA, et al. Serologic evidence of respiratory and rickettsial infections among Somali refugees. Am J Trop Med Hyg. 1995;52(4):349-53. doi: 10.4269/ajtmh.1995.52.349.

62. Grecchi C, Zanotti P, Pontarelli A, Chiari E, Tomasoni LR, Gulletta M, et al. Louse-borne relapsing fever in a refugee from Mali. Infection. 2017;45(3):373-6.

63. Gumisiriza N, Kugler M, Brusselaers N, Mubiru F, Anguzu R, Ningwa A, et al. Risk factors for nodding syndrome and other forms of epilepsy in northern uganda: A case-control study. Pathogens. 2021;10(11). doi:10.3390/pathogens10111451.

64. Gurses G, Ozaslan M, Zeyrek FY, Kilic IH, Doni NY, Karagoz ID, et al. Molecular identification of Leishmania spp. isolates causes cutaneous leishmaniasis (CL) in Sanliurfa Province, Turkey, where CL is highly endemic. Folia Microbiol (Praha). 63(3):353-9.

65. Guthmann JP, Klovstad H, Boccia D, Hamid N, Pinoges L, Nizou JY, et al. A large outbreak of hepatitis E among a displaced population in Darfur, Sudan, 2004: the role of water treatment methods. Clin Infect Dis. 2006;42(12):1685-91. doi: 10.1086/504321.

66. Enterically transmitted non-A, non-B hepatitis--East Africa. MMWR. 1987;36(16):241-4.

67. Harris AR, Russell RJ, Charters AD. A review of schistosomiasis in immigrants in Western Australia, demonstrating the unusual longevity of Schistosoma mansoni. Trans R Soc Trop Med Hyg. 1984;78(3):385-8.

68. Hassan AO, Mero WMS. Prevalence of intestinal parasites among displaced people living in displacement camps in duhok province/Iraq. Internet Journal of Microbiology. 2020;17(1).

69. Heudorf U, Steul K, Gottschalk R. Sars-Cov-2 in children - insights and conclusions from the mandatory reporting data in Frankfurt am Main, Germany, March-July 2020. Gms Hygiene and Infection Control. 2020;15:12.

70. Hijawi KJF, Hijjawi NS, Ibbini JH. Detection, genotyping, and phylogenetic analysis of Leishmania isolates collected from infected Jordanian residents and Syrian refugees who suffered from cutaneous leishmaniasis. Parasitol Res. 2019;118(3):793-805.

71. Hoch M, Wieser A, Löscher T, Margos G, Pürner F, Zühl J, et al. Louse-borne relapsing fever (Borrelia recurrentis) diagnosed in 15 refugees from northeast Africa: Epidemiology and preventive control measures, Bavaria, Germany, July to October 2015. Euro Surveill. 2015;20(42).

72. Inci R, Ozturk P, Mulayim MK, Ozyurt K, Alatas ET, Inci MF. Effect of the Syrian civil war on prevalence of cutaneous leishmaniasis in Southeastern Anatolia, Turkey. Medical Science Monitor. 2015;21:5.

73. Jamal Q, Shah A, Ali N, Ashraf M, Awan MM, Lee CM. Prevalence and comparative analysis of cutaneous leishmaniasis in Dargai region in Pakistan. Pakistan Journal of Zoology. 2013;45(2):537-41.

74. Keittivuti B, D'Agnes T, Keittivuti A, Viravaidya M. Prevalence of schistosomiasis and other parasitic diseases among Cambodian refugees residing in Bang-Kaeng holding center, Prachinburi Province, Thailand. Am J Trop Med Hyg. 1982;31(5):988-90.

75. Keittivuti B, Keittivuti A, D'Agnes T. Schistosomiasis in Cambodian refugees at Ban-Kaeng holding centre, Prachinburi province, Thailand. Southeast Asian J Trop Med Public Health. 1982;13(2):216-9.

76. Keittivuti B, Keittivuti A, O'Rourke T, D'Agnes T. Treatment of Schistosoma mekongi with praziquantel in Cambodian refugees in holding centres in Prachinburi Province, Thailand. Trans R Soc Trop Med Hyg. 1984;78(4):477-9.

77. Keittivuti B, Keittivuti A, O'Rourke TF. Parasitic diseases with emphasis on schistosomiasis in Cambodian refugees, in Prachinburi Province Thailand. Southeast Asian J Trop Med Public Health. 1983;14(4):491-4.

78. Khachfe HH, Zayyoun FJ, Sharif-Askari E, Ramadan W, Hallal N, Khachfe HM. Effect of leishmaniasis on the performance of elementary school students: A case study among syrian refugees in some bekaa (lebanon) area schools. J Epidemiol Glob Health. 2019;9(4):266-73.

79. Khan MI, Muhammad M, Khan W, Khan N, Noor SM. Nasal involvement in cutaneous leishmaniasis. Bangladesh Med Res Counc Bull. 2010;24(3):202-6.

80. Khan S, Akbar SMF, Kimitsuki K, Saito N, Yahiro T, Al Mahtab M, et al. Recent downhill course of COVID-19 at Rohingya refugee camps in Bangladesh: Urgent action solicited. J Glob Health. 2021;11:03097. doi: 10.7189/jogh.11.03097.

81. Kjersem H, Jepsen S, Larsen L, Black F. Salmonella and Shigella carriers among refugees from the Middle East and Sri Lanka in Denmark. Scandinavian Journal of Social Medicine. 1990;18(3):175-8.

82. Knust B, Wongjindanon N, Moe AA, Herath L, Kaloy W, Soe TT, et al. Enhancing respiratory disease surveillance to detect COVID-19 in shelters for displaced persons, Thailand-Myanmar Border, 2020-2021. Emerg Infect Dis. 2022;28(13):S17-s25. doi:10.3201/eid2813.220324.

83. Kolaczinski J, Brooker S, Reyburn H, Rowland M. Epidemiology of anthroponotic cutaneous leishmaniasis in Afghan refugee camps in northwest Pakistan. Transactions of the Royal Society of Tropical Medicine and Hygiene. 2004;98(6):373-8.

84. Kortas AZ, Polenz J, von Hayek J, Rüdiger S, Rottbauer W, Storr U, et al. Screening for infectious diseases among asylum seekers newly arrived in Germany in 2015: a systematic single-centre analysis. Public Health. 2017;153:1-8.

85. Lagare A, Ibrahim A, Ousmane S, Issaka B, Zaneidou M, Kadadé G, et al. Outbreak of hepatitis E virus infection in displaced persons camps in Diffa region, Niger, 2017. Am J Trop Med Hyg. 2018;99(4):1055-7.

86. Le Bihan C, Faucherre V, Le Moing V, Mehenni A, Nantes D, Da Silva A, et al. COVID-19: The forgotten cases of hidden exiles. Infect Dis Now. 2021.

87. Lin CY, Chen TC, Dai CY, Yu ML, Lu PL, Yen JH, et al. Serological investigation to identify risk factors for post-flood infectious diseases: a longitudinal survey among people displaced by Typhoon Morakot in Taiwan. BMJ Open. 2015;5(5):e007008.

88. Ly TDA, Nguyen NN, Hoang VT, Goumballa N, Louni M, Canard N, et al. Screening of SARS-CoV-2 among homeless people, asylum-seekers and other people living in precarious conditions in Marseille, France, March–April 2020. International Journal of Infectious Diseases. 2021;105:1-6.

89. Maaßen W, Wiemer D, Frey C, Kreuzberg C, Tannich E, Hinz R, et al. Microbiological screenings for infection control in unaccompanied minor refugees: The German Armed Forces Medical Service's experience. Military Medical Research volume. 2017;4(1).

90. Mazhar MKA, Finger F, Evers ES, Kuehne A, Ivey M, Yesurajan F, et al. An outbreak of acute jaundice syndrome (AJS) among the Rohingya refugees in Cox’s Bazar, Bangladesh: Findings from enhanced epidemiological surveillance. PLoS One. 2021;16(4). doi:10.1371/journal.pone.0250505.

91. McCleery EJ, Patchanee P, Pongsopawijit P, Chailangkarn S, Tiwananthagorn S, Jongchansittoe P, et al. Taeniasis among refugees living on Thailand–Myanmar border, 2012. Emerg Infect Dis. 2015;21(10):1824-6.

92. McGready R, Ashley EA, Wuthiekanun V, Tan SO, Pimanpanarak M, Viladpai-Nguen SJ, et al. Arthropod borne disease: The leading cause of fever in pregnancy on the thai-burmese border. PLoS Negl Trop Dis. 2010;4(11).

93. Mekonnen GK, Mengistie B, Sahilu G, Kloos H, Mulat W. Etiologies of diarrhea and drug susceptibility patterns of bacterial isolates among under-five year children in refugee camps in Gambella Region, Ethiopia: a case control study. BMC Infect Dis. 2019;19(1).

94. Mellou K, Gkolfinopoulou K, Andreopoulou A, Tsekou A, Papadima K, Stamoulis K, et al. A COVID-19 outbreak among migrants in a hosting facility in Greece, April 2020. Journal of Infection Prevention. 2022;23(5):235-8. doi:10.1177/17571774221092568.

95. Mérens A, Guérin PJ, Guthmann JP, Nic, E. Outbreak of hepatitis E virus infection in Darfur, Sudan: Effectiveness of real-time reverse transcription-PCR analysis of dried blood spots. J Clin Microbiol. 2009;47(6):1931-3.

96. Miladinovic-Tasic NL, Tasic SA, Kranjcic-Zec I, Tasic G, Tasic A, Tasic IS. Asymptomatic giardiasis-more prevalent in refugees than in native inhabitants of the city of Nis, Serbia. Central European Journal of Medicine. 2008;3(2):203-6.

97. Miller JM, Boyd HA, Ostrowski SR, Cookson ST, Parise ME, Gonzaga PS, et al. Malaria, intestinal parasites, and schistosomiasis among Barawan Somali refugees resettling to the United States: A strategy to reduce morbidity and decrease the risk of imported infections. Am J Trop Med Hyg. 2000;62(1):115-21.

98. Mitchell T, Lee D, Weinberg M, Phares C, James N, Amornpaisarnloet K, et al. Impact of Enhanced Health Interventions for United States-Bound Refugees: Evaluating best practices in migration health. Am J Trop Med Hyg. 2018;98(3):920-8.

99. Nicand E, Armstrong GL, Enouf V, Guthmann JP, Guerin JP, Caron M, et al. Genetic heterogeneity of hepatitis E virus in Darfur, Sudan, and neighboring Chad. Journal of Medical Virology. 2005;77(4):519-21.

100. Ntais P, Christodoulou V, Tsirigotakis N, Dokianakis E, Dedet J-P, Pratlong F, et al. Will the introduction of Leishmania tropica MON-58, in the island of Crete, lead to the settlement and spread of this rare zymodeme? Acta Tropica. 2014;132:125-30. doi:10.1016/j.actatropica.2014.01.003.

101. Nyakarahuka L, Whitmer S, Kyondo J, Mulei S, Cossaboom CM, Telford CT, et al. Crimean-Congo hemorrhagic fever outbreak in refugee settlement during COVID-19 pandemic, Uganda, April 2021. Emerg Infect Dis. 2022;28(11):2326-9. doi:10.3201/eid2811.220365.

102. Nyamusore J, Nahimana MR, Ngoc CT, Olu O, Isiaka A, Ndahindwa V, et al. Risk factors for transmission of Salmonella Typhi in Mahama refugee camp, Rwanda: a matched case-control study. Pan African Medical Journal. 2018;29:13.

103. Oboth P, Gavamukulya Y, Barugahare BJ. Prevalence and clinical outcomes of Plasmodium falciparum and intestinal parasitic infections among children in Kiryandongo refugee camp, mid-Western Uganda: A cross sectional study. BMC Infect Dis. 2019;19(1).

104. Palacios CF, Tucker EW, Travassos MA. Coronavirus Disease 2019 Burden among unaccompanied minors in US custody. Clinical Infectious Diseases. 2022. doi:10.1093/cid/ciac636.

105. Patamia I, Nicotra P, Amodeo D, Giuliano L, Cicero CE, Nicoletti A. Geo-helminthiasis among migrants in Sicily: a possible focus for re-emerging neurocysticercosis in Europe. Neurological Sciences. 2017;38(6):1105-7.

106. Pohl C, Mack I, Schmitz T, Ritz N. The spectrum of care for pediatric refugees and asylum seekers at a tertiary health care facility in Switzerland in 2015. Eur J Pediatr. 2017;176(12):1681-7.

107. Raoult D, Ndihokubwayo JB, Tissot-Dupont H, Roux V, Faugere B, Abegbinni R, et al. Outbreak of epidemic typhus associated with trench fever in Burundi. The Lancet. 1998;352(9125):353-8.

108. Redditt V, Wright V, Rashid M, Male R, Bogoch I. Outbreak of SARS-CoV-2 infection at a large refugee shelter in Toronto, April 2020: a clinical and epidemiologic descriptive analysis. CMAJ Open. 2020;8(4):E819-e24.

109. Relić T, Kačarević H, Ilić N, Jovanović D, Tambur Z, Doder R, et al. Intestinal parasitosis in asylum seekers from the middle east and South Asia. Vojnosanitetski pregled. 2018;75(11):1101-5.

110. Richter J, Bode JG, Blondin D, Kircheis G, Kubitz R, Holtfreter MC, et al. Severe liver fibrosis caused by Schistosoma mansoni: Management and treatment with a transjugular intrahepatic portosystemic shunt. The Lancet Infectious Diseases. 2015;15(6):731-7.

111. Rowland M, Munir A, Durrani N, Noyes H, Reyburn H. An outbreak of cutaneous leishmaniasis in an Afghan refugee settlement in north-west Pakistan. J Transactions of the Royal Society of Tropical Medicine and Hygiene. 1999;93(2):133-6.

112. Ryan N, Plackett M, Dwyer B. Parasitic infections of refugees. Medical Journal of Australia. 1988;148(10):491-4. doi: https://doi.org/10.5694/j.1326-5377.1988.tb99455.x.

113. Saab M, El Hage H, Charafeddine K, Habib RH, Khalifeh I. Diagnosis of cutaneous leishmaniasis: Why punch when you can scrape? Am J Trop Med Hyg. 2015;92(3):518-22.

114. Saikal SL, Ge L, Mir A, Pace J, Abdulla H, Leong KF, et al. Skin disease profile of Syrian refugees in Jordan: a field-mission assessment. J Eur Acad Dermatol Venereol. 2020;34(2):419-25. doi: 10.1111/jdv.15909.

115. Samuda GM, Chan SP, Yeung CY. Vietnamese child health in a Hong Kong closed camp. Aust Paediatr J. 1988;24(2):115-7.

116. Saroufim M, Charafeddine K, Issa G, Khalifeh H, Habib RH, Berry A, et al. Ongoing epidemic of cutaneous leishmaniasis among Syrian refugees, Lebanon. Emerg Infect Dis. 2014;20(10):1712-5.

117. Schroeder Jr HW, Yarrish RL, Perkins TF, Lee C. Sequential disseminated tuberculosis and toxoplasmosis in a Haitian refugee. Southern Medical Journal. 1984;77(4):533-4.

118. Schweickert B, Bollmann R, Loui A, Kaufmann O, Kluttig L, Feiterna-Sperling C, et al. Fatal disseminated toxoplasmosis with congenital transmission in an African migrant. AIDS (London, England). 2008;22(12):1523-5.

119. Sencan I, Sahin I, Kaya D, Oksuz S, Yildirim M. Assessment of HAV and HEV seroprevalence in children living in post-earthquake camps from Düzce, Turkey. Eur J Epidemiol. 2004;19(5):461-5.

120. Seybolt LM, Christiansen D, Barnett ED. Diagnostic evaluation of newly arrived asymptomatic refugees with eosinophilia. Clinical Infectious Diseases. 2006;42(3):363-7.

121. Sharov KS. SARS-CoV-2 spread in different biosocial strata in Russia in 2020: Groups of risk and victimised groups. Journal of Global Health. 2021;11. doi:10.7189/jogh.11.03066.

122. Shorter D, Makone I, Elliott EJ. Fever and urticaria in an African refugee. Journal of Paediatrics and Child Health. 2006;42(11):731-3.

123. Sisti LG, Di Napoli A, Petrelli A, Rossi A, Diodati A, Menghini M, et al. Covid-19 impact in the italian reception system for migrants during the nationwide lockdown: A national observational study. International Journal of Environmental Research and Public Health. 2021;18(23). doi:10.3390/ijerph182312380.

124. Spicher VM, Genin B, Jordan AR, Rubbia-Brandt L, Le Coultre C. Peritoneal schistosomiasis: an unusual laparoscopic finding. J Pediatr Surg. 2004;39(4):631-3.

125. Stehr-Green JK, Schantz PM. Trichinosis in Southeast Asian refugees in the United States. Am J Public Health. 1986;76(10):1238-9.

126. Storer E, Wayte J. Cutaneous leishmaniasis in Afghani refugees. Australasian Journal of Dermatology. 2005;46(2):80-3.

127. Sulaiman AA, Elmadhoun WM, Noor SK, Bushara SO, Almobarak AO, Awadalla H, et al. An outbreak of cutaneous leishmaniasis among a displaced population in North Sudan: Review of cases. J Family Med Prim Care. 2019;8(2):556-63.

128. Sulekova LF, Ceccarelli G, Pombi M, Esvan R, Lopalco M, Vita S, et al. Occurrence of intestinal parasites among asylum seekers in Italy: A cross-sectional study. Travel Med Infect Dis. 2018;27:46-52.

129. Sullivan R, Linneman Jr CC, Clark CS, Walzer PD. Seroepidemiologic study of giardiasis patients and high-risk groups in a midwestern city in the United States. Am J Public Health. 1987;77(8):960-3.

130. Swanson SJ, Phares CR, Mamo B, Smith KE, Cetron MS, Stauffer WM. Albendazole therapy and enteric parasites in United States-bound refugees. New England Journal of Medicine. 2012;366(16):1498-507.

131. Tambuzzi S, Cummaudo M, Maggioni L, Tritella S, Lucchesi B, Montedoro P, et al. A pilot COVID-19 surveillance program at the Zendrini center in Milan (Italy) for unaccompanied foreign minors. Children (Basel). 2022;9(10). doi:10.3390/children9101485.

132. Tappe D, Weise D, Ziegler U, Müller A, Müllges W, Stich A. Brain and lung metastasis of alveolar echinococcosis in a refugee from a hyperendemic area. J Med Microbiol. 2008;57(11):1420-3.

133. Taylor DN, Echeverria P, Pitarangsi C, Seriwatana J, Sethabutr O, Bodhidatta L, et al. Application of DNA hybridization techniques in the assessment of diarrheal disease among refugees in Thailand. Am J Epidemiol. 1988;127(1):179-87.

134. Taylor R. Typhoid fever in the Basque Refugee Camp. British Medical Journal. 1937;1937:760-1.

135. Temcharoen P, Viboolyavatana J, Tongkoom B. A survey on intestinal parasitic infections in Laotian refugees at Ubon Province, northeastern Thailand, with special reference to schistosomiasis. Southeast Asian J Trop Med Public Health. 1979;10(4):552-5.

136. Theuring S, Friedrich-Janicke B, Portner K, Trebesch I, Durst A, Dieckmann S, et al. Screening for infectious diseases among unaccompanied minor refugees in Berlin, 2014-2015. Eur J Epidemiol. 2016;31(7):707-10.

137. Thomson K, Luis Dvorzak J, Lagu J, Laku R, Dineen B, Schilperoord M, et al. Investigation of hepatitis E outbreak among refugees - Upper Nile, South Sudan, 2012-2013. MMWR. 2013;62(29):581-6.

138. Turunen T, Kontunen K, Sugulle K, Hieta P, Snellman O, Hussein I, et al. COVID-19 outbreak at a reception centre for asylum seekers in Espoo, Finland. J Migr Health. 2021;3:100043.

139. Ul Haq KA, Gul NA, Muhammad Hammad H, Bibi Y, Bibi A, Mohsan J. Prevalence of giardia intestinalis and hymenolepis nana in afghan refugee population of mianwali district, pakistan. African Health Sciences. 2015;15(2):394-400.

140. Vallejo-Janeta AP, Morales-Jadan D, Freire-Paspuel B, Lozada T, Cherrez-Bohorquez C, Garcia-Bereguiain MA, et al. COVID-19 outbreaks at shelters for women who are victims of gender-based violence from Ecuador. International Journal of Infectious Diseases. 2021;108:531-6. doi:10.1016/j.ijid.2021.06.012.

141. Van Boetzelaer E, Fotso A, Angelova I, Huisman G, Thorson T, Hadj-Sahraoui H, et al. Health conditions of migrants, refugees and asylum seekers on search and rescue vessels on the central Mediterranean Sea, 2016-2019: A retrospective analysis. BMJ Open. 2022;12(1). doi:10.1136/bmjopen-2021-053661.

142. Van Enter BJD, Lau YL, Ling CL, Watthanaworawit W, Sukthana Y, Lee WC, et al. Seroprevalence of toxoplasma gondii infection in refugee and migrant pregnant women along the Thailand-myanmar border. Am J Trop Med Hyg. 2017;97(1):232-5.

143. Wamala JF, Loro F, Deng SJ, Berta KK, Guyo AG, Mpairwe A, et al. Epidemiological characterization of COVID-19 in displaced populations of South Sudan. Pan African Medical Journal. 2022;41(2). doi:10.11604/pamj.supp.2022.42.1.33767.

144. Webster JL, Stauffer WM, Mitchell T, Lee D, O’Connell EM, Weinberg M, et al. Cross-sectional assessment of the association of eosinophilia with intestinal parasitic infection in U.S.-bound refugees in Thailand: Prevalent, age dependent, but of limited clinical utility. Am J Trop Med Hyg. 2022;106(5):1552-9. doi:10.4269/ajtmh.21-0853.

145. Wilting KR, Stienstra Y, Sinha B, Braks M, Cornish D, Grundmann H. Louse-borne relapsing fever (Borrelia recurrentis) in asylum seekers from Eritrea, the Netherlands, July 2015. Euro Surveill. 2015;20(30):2-4.

146. Yauba SM, Rabasa AI, Farouk AG, Elechi HA, Ummate I, Ibrahim BA, et al. Urinary schistosomiasis in Boko Haram-related internally displaced Nigerian children. Saudi J Kidney Dis Transpl. 2018;29(6):1395-402.

147. Zijlstra EE, Ali MS, El-Hassan AM, El-Toum IA, Satti M, Ghalib Kager HWPA. Direct agglutination test for diagnosis and sero- epidemiological survey of kala-azar in the Sudan. Trans R Soc Trop Med Hyg. 1991;85(4):474-6.

148. Zijlstra EE, Siddig Ali M, El-Hassan AM, El-Toum IA, Satti M, Ghalib HW, et al. Kala-azar in displaced people from southern Sudan: Epidemiological, clinical and therapeutic findings. Trans R Soc Trop Med Hyg. 1991;85(3):365-9.

149. Abd Rahman MM, Bryant P, Guppy D, Buttery J, Burgner D. Intermittent fever, splenomegaly and eosinophilia in a recently resettled African refugee. J Paediatr Child Health. 2012;48(10):939-41.

150. Ahmed W, Ahmad M, Rafatullah, Shah F, Sajadullah. Pervasiveness of intestinal protozoan and worm incursion in IDP's (North Waziristan agency, KPK-Pakistan) children of 6-16 years. Journal of the Pakistan Medical Association. 2015;65(9):943-5.

151. Aksin S, Cim N, Andan C, Tunc S, Goklu MR. Comparison of obstetric and infectious results among Syrian pregnant women. Annals of Clinical and Analytical Medicine. 2021;12(5):501-5. doi:10.4328/acam.20411.

152. Alberer M, Malinowski S, Sanftenberg L, Schelling J. Notifiable infectious diseases in refugees and asylum seekers: experience from a major reception center in Munich, Germany. Infection. 2018;46(3):375-83.

153. Aldulaimi S, Mendez A. Chronic abdominal pain and hepatosplenomegaly in a refugee patient. Travel Med Infect Dis. 2021;41:102009.

154. Aliskin O, Savas N. Notifiable communicable diseases in Turkey and their notification status: Antakya Sample. 2019;24(1):11-21.

155. Armitage AJ, Cohen J, Heys M, Hardelid P, Ward A, Eisen S. Description and evaluation of a pathway for unaccompanied asylum-seeking children. Archives of disease in childhood. 2022;107(5):456-60. doi:10.1136/archdischild-2021-322319.

156. Baggio S, Jacquerioz F, Salamun J, Spechbach H, Jackson Y. Equity in access to COVID-19 testing for undocumented migrants and homeless persons during the initial phase of the pandemic. Journal of Migration and Health. 2021;4. doi:10.1016/j.jmh.2021.100051.

157. Barbier D, Demenais F, Lefait JF, David B, Blanc M, Hors J, et al. Susceptibility to human cutaneous leishmaniasis and HLA, Gm, Km markers. Tissue Antigens. 1987;30(2):63-7.

158. Beltrame A, Buonfrate D, Gobbi F, Angheben A, Marchese V, Monteiro GB, et al. The hidden epidemic of schistosomiasis in recent African immigrants and asylum seekers to Italy. European Journal of Epidemiology. 2017;32(8):733-5.

159. Borch M, Kiernan M, Rust K, Baron B, Simmons B, Hattala P, et al. Schistosomiasis: a case study. Urol Nurs. 2009;29(1):26-9.

160. Brodine SK, Thomas A, Huang R, Harbertson J, Mehta S, Leake J, et al. Community based parasitic screening and treatment of sudanese refugees: Application and assessment of centers for disease control guidelines. Am J Trop Med Hyg. 2009;80(3):425-30.

161. Caruana SR, Kelly HA, Ngeow JYY, Ryan NJ, Bennett CM, Chea L, et al. Undiagnosed and potentially lethal parasite infections among immigrants and refugees in Australia. Journal of Travel Medicine. 2006;13(4):233-9.

162. da Costa e Silva GR, Martins TLS, de Almeida Silva C, Caetano KAA, dos Santos Carneiro MA, Silva BVDE, et al. Hepatitis A and E among immigrants and refugees in Central Brazil. Revista de Saude Publica. 2022;56. doi:10.11606/S1518-8787.2022056003839.

163. da Silva HP, Abreu IN, Lima CNC, de Lima ACR, Barbosa AD, de Oliveira LR, et al. Migration in times of pandemic: SARS-CoV-2 infection among the Warao indigenous refugees in Belem, Para, Amazonia, Brazil. BMC Public Health. 2021;21(1). doi:10.1186/s12889-021-11696-7.

164. Francke E. Medical evaluation of Indochinese refugees conditions to consider. Postgraduate Medicine. 1982;72(5):92-3.

165. Franco-Paredes C, Dismukes R, Nicolls D, Hidron A, Workowski K, Rodriguez-Morales A, et al. Persistent and untreated tropical infectious diseases among Sudanese refugees in the United States. Am J Trop Med Hyg. 2007;77(4):633-5.

166. Franco-Paredes C, Nicolls D, Kempker R, Dismukes R, Kozarsky P. Pelvic echinococcosis in a Northern Iraqi refugee. J Travel Med. 2006;13(2):119-22.

167. Hanapi IRM, Sahimin N, Maackara MJB, Annisa AS, Mutalib R, Lewis JW, et al. Prevalence of anti-Leptospira antibodies and associated risk factors in the Malaysian refugee communities. BMC Infect Dis. 2021;21(1). doi:10.1186/s12879-021-06830-0.

168. Harkensee C, Andrew R. Health needs of accompanied refugee and asylum-seeking children in a UK specialist clinic. Acta Paediatr. 2021;110(8):2396-404. doi: 10.1111/apa.15861.

169. Hershko C, Nesher G, Yinnon AM. Medical problems in Ethiopian refugees airlifted to Israel: Experience in 131 patients admitted to a general hospital. J Trop Med Hyg. 1986;89(3):107-12.

170. Hertting O, Luth, er J, Giske CG, Bennet R, Eriksson M. Acute infection as cause of hospitalization of asylum-seeking children and adolescents in Stockholm, Sweden 2015-2016. European Journal of Pediatrics. 2021;180(3):893-8.

171. IsaÃ¤cson M, Frean J, He J, Seriwatana J, Innis BL. An outbreak of hepatitis E in Northern Namibia, 1983. Am J Trop Med Hyg. 2000;62(5):619-25. doi: 10.4269/ajtmh.2000.62.619.

172. Jacoby H, Rawling RA, Granato PA. Cutaneous Leishmaniasis in a Central American Refugee. Clinical Microbiology Newsletter. 2014;36(3):22-4.

173. Lerman D, Barrett-Connor E, Norcross W. Intestinal parasites in asymptomatic adult Southeast Asian immigrants. J Fam Pract. 1982;15(3):443-6.

174. Lifson AR, Thai D, O'Fallon A, Mills WA, Hang K. Prevalence of tuberculosis, hepatitis B virus, and intestinal parasitic infections among refugees to Minnesota. Public Health Rep. 2002;117(1):69-77.

175. Lindner AK, Richter J, Gertler M, Nikolaus M, Martinez GE, Muller K, et al. Cutaneous leishmaniasis in refugees from Syria: complex cases in Berlin 2015-2020. Journal of Travel Medicine. 2020;27(7):8.

176. Lowther SA, Johnson G, Hendel-Paterson B, Nelson K, Mamo B, Krohn K, et al. HIV/AIDS and associated conditions among HIV-infected refugees in Minnesota, 2000–2007. Int J Environ Res Public Health. 2012;9(11):4197-209. doi: 10.3390/ijerph9114197.

177. Lurio J, Verson H, Karp S. Intestinal parasites in Cambodians: comparison of diagnostic methods used in screening refugees with implications for treatment of populations with high rates of infestation. J Am Board Fam Pract. 1991;4(2):71-8. Epub 1991/03/01.

178. Malamitsi-Puchner A, Papacharitonos S, Sotos D, Tzala L, Psichogiou M, Hatzakis A, et al. Prevalence study of different hepatitis markers among pregnant Albanian refugees in Greece. European Journal of Epidemiology. 1996;12(3):297-301.

179. Marlet MVL, Wuillaume F, Jacquet D, Quispe KW, Dujardin JC, Boelaert M. A neglected disease of humans: A new focus of visceral leishmaniasis in Bakool, Somalia. Trans R Soc Trop Med Hyg. 2003;97(6):667-71.

180. Marnell F, Guillet A, Holl, C. A survey of the intestinal helminths of refugees in Juba, Sudan. Annals of Tropical Medicine and Parasitology. 1992;86(4):387-93.

181. Martin JA, Mak DB. Changing faces: a review of infectious disease screening of refugees by the Migrant Health Unit, Western Australia in 2003 and 2004. Medical Journal of Australia. 2006;185(11):607-10.

182. Masters PJ, Lanfranco PJ, Sneath E, Wade AJ, Huffam S, Pollard J, et al. Health issues of refugees attending an infectious disease refugee health clinic in a regional Australian hospital. Australian Journal of General Practice. 2018;47(5):305-10.

183. McAuley JB, Michelson MK, Hightower AW, Engeran S, Wintermeyer LA, Schantz PM. A trichinosis outbreak among Southeast Asian refugees. Am J Epidemiol. 1992;135(12):1404-10.

184. McDowell D, Harper CG. Neurocysticercosis - Two Australian cases. Med J Aust. 1990;152(4):217-8.

185. Meropol SB. Health status of pediatric refugees in Buffalo, NY. Arch Pediatr Adolesc Med. 1995;149(8):887-92.

186. Moaven L, Van Asten M, Crofts N, Locarnini SA. Seroepidemiology of hepatitis E in selected Australian populations. J Med Virol. 1995;45(3):326-30.

187. Mockenhaupt FP, Barbre KA, Jensenius M, Larsen CS, Barnett ED, Stauffer W, et al. Profile of illness in syrian refugees: A geosentinel analysis, 2013 to 2015. Eurosurveillance. 2016;21(10).

188. Molina CD, Molina MM, Molina JM. Intestinal parasites in Southeast Asian refugees two years after immigration. West J Med. 1988;149(4):422-5.

189. Montour J, Lee D, Snider C, Jentes ES, Stauffer W. Absence of Loa loa microfilaremia among newly arrived congolese refugees in Texas. Am J Trop Med Hyg. 2017;97(6):1833-5.

190. Müller F, Chandra S, Bogoch II, Rashid M, Redditt V. Intestinal parasites in stool testing among refugees at a primary care clinic in Toronto, Canada. BMC Infect Dis. 2022;22(1). doi:10.1186/s12879-022-07226-4.

191. Mutch RC, Cherian S, Nemba K, Geddes JS, Rutherford DM, Chaney GM, et al. Tertiary paediatric refugee health clinic in Western Australia: Analysis of the first 1026 children. Journal of Paediatrics and Child Health. 2012;48(7):582-7.

192. Newman RD, Schwartz MA. Hematuria in two school-age refugee brothers from Africa. Pediatr Emerg Care. 1999;15(5):335-7.

193. Ofoezie IE, Asaulu SO, Christensen NØ, Madsen H. Patterns of infection with Schistosoma haematobium in lakeside resettlement communities at the Oyan Reservoir in Ogun State, south-western Nigeria. Ann Trop Med Parasitol. 1997;91(2):187-97.

194. Özbilgin A, Gencoglan G, Tunali V, Çavuş İ, Yıldırım A, Gündüz C, et al. Refugees at the crossroads of continents: A molecular approach for cutaneous leishmaniasis among refugees in Turkey. Acta Parasitologica. 2020;65(1):136-43.

195. Paran Y, Ben-Ami R, Orlev B, Halutz O, Elalouf O, Wasserman A, et al. Chronic schistosomiasis in African immigrants in Israel: Lessons for the non-endemic setting. Medicine (Baltimore). 2019;98(52).

196. Paxton GA, Sangster KJ, Maxwell EL, McBride CRJ, Drewe RH. Post-arrival health screening in Karen refugees in Australia. PLoS One. 2012;7(5).

197. Perea WA, Ancelle T, Moren A, Nagelkerke M, Sondorp E. Visceral leishmaniasis in southern Sudan. Trans R Soc Trop Med Hyg. 1991;85(1):48-53.

198. Pham PN, Keegan K, Johnston LG, Rodas J, Restrepo MA, Wei C, et al. Assessing the impact of the COVID-19 pandemic among Venezuelan refugees and migrants in Colombia using respondent-driven sampling (RDS). BMJ Open. 2022;12(10). doi:10.1136/bmjopen-2021-054820.

199. Poddighe D, Castelli L, Pulcrano G, Grosini A, Balzaretti M, Spadaro S, et al. Urinary schistosomiasis in an adolescent refugee from Africa: An uncommon cause of hematuria and an emerging infectious disease in Europe. J Immigr Minor Health. 2016;18(5):1237-40.

200. Posey DL, Blackburn BG, Weinberg M, Flagg EW, Ortega L, Wilson M, et al. High prevalence and presumptive treatment of schistosomiasis and strongyloidiasis among African refugees. Clinical Infectious Diseases. 2007;45(10):1310-5.

201. Prodanuk M, Wagner S, Orkin J, Noone D. Social vulnerability and COVID-19: A call to action for paediatric clinicians. Paediatrics and Child Health (Canada). 2021;26(1):1-3. doi:10.1093/pch/pxaa121.

202. Qazi M, Weimer AC, Bedard BA, Kennedy BS. Q-fever in a refugee after exposure to a central New York State livestock farm. Annals of Tropical Medicine and Public Health. 2016;9(4):266-70.

203. Quandelacy TM, Riefkohl A, Franco-Paredes C. Prevalence of untreated schistosomiasis among Sudanese refugees: “The Lost Boys of Sudan” in the United States. Boletin medico del Hospital Infantil de Mexico. 2010;67:503-6.

204. Rab MA, al Rustamani L, Bhutta RA, Mahmood MT, Evans DA. Cutaneous leishmaniasis: iso-enzyme characterisation of Leishmania tropica. J Pak Med Assoc. 1997;47(11):270-3.

205. Ralli M, Cedola C, Urbano S, Latini O, Shkodina N, Morrone A, et al. Assessment of SARS-CoV-2 infection through rapid serology testing in the homeless population in the City of Rome, Italy. Preliminary results. J Public Health Res. 2020;9(4):556-9.

206. Raman S, Wood N, Webber M, Taylor KA, Isaacs D. Matching health needs of refugee children with services: how big is the gap? Aust N Z J Public Health. 2009;33(5):466-70.

207. Ravensbergen SJ, Lokate M, Cornish D, Kloeze E, Ott A, Friedrich AW, et al. High prevalence of infectious diseases and drug-resistant microorganisms in asylum seekers admitted to hospital; no carbapenemase producing Enterobacteriaceae until September 2015. PLoS One. 2016;11(5):e0154791.

208. Schmid M, Dodt C. Multiple Organ Failure in a Young Asylum-Seeker. Dtsch Arztebl Int. 2017;114(37):625.

209. Sheikh M, Pal A, Wang S, MacIntyre CR, Wood NJ, Isaacs D, et al. The epidemiology of health conditions of newly arrived refugee children: A review of patients attending a specialist health clinic in Sydney. Journal of Paediatrics and Child Health. 2009;45(9):509-13.

210. Shen C, Li S, Zheng S, Choi MH, Bae YM, Hong ST. Tissue parasitic helminthiases are prevalent at Cheongjin, North Korea. Korean J Parasitol. 2007;45(2):139-44.

211. Southwood T, Davidson GP, Phillips GE, Rice M. Hepatosplenic schistosomiasis in a South-East Asian refugee child in South Australia. Aust N Z J Med. 1983;13(4):384-6.

212. Steele LS, MacPherson DW, Kim J, Keystone JS, Gushulak BD. The sero-prevalence of antibodies to Trypanosoma cruzi in Latin American refugees and immigrants to Canada. Journal of Immigrant and Minority Health. 2007;9(1):43-7. doi: 10.1007/s10903-006-9014-x.

213. Summer AP, Stauffer W, Maroushek SR, Nevins TE. Hematuria in children due to schistosomiasis in a nonendemic setting. Clin Pediatr (Phila). 2006;45(2):177-81.

214. Tiong ACD, Patel MS, Gardiner J, Ryan R, Linton KS, Walker KA, et al. Health issues in newly arrived African refugees attending general practice clinics in Melbourne. Medical Journal of Australia. 2006;185(11):602-6.

215. Tittle BS, Harris JA, Chase PA. Health screening of Indochinese refugee children. Am J Dis Child. 1982;136(8):697-700.

216. Tolunay O, Çelik Ü, Arslan I, Tutun B, Özkaya M. Evaluation of clinical findings and treatment results of Coronavirus disease 2019 (COVID-19) in pediatric cancer patients: A single center experience. Frontiers in Pediatrics. 2022;10. doi: doi:10.3389/fped.2022.848379.

217. Yangco BG, Vincent AL, Vickery AC. A survey of filariasis among refugees in South Florida. Am J Trop Med Hyg. 1984;33(2):246-51.

218. Zhang M, Gurung A, Anglewicz P, Baniya K, Yun K. Discrimination and stress among Asian refugee populations during the COVID-19 pandemic: Evidence from Bhutanese and Burmese refugees in the USA. Journal of Racial and Ethnic Health Disparities. 2022;9(2):589-97. doi:10.1007/s40615-021-00992-y.

219. Zöllkau J, Ankert J, Pletz MW, Mishra S, Seliger G, Lobmaier SM, et al. Hepatitis E, schistosomiasis and echinococcosis–Prevalence in a cohort of pregnant migrants in Germany and their influence on fetal growth restriction. Pathogens. 2022;11(1). doi:10.3390/pathogens11010058.

220. Alawieh A, Musharrafieh U, Jaber A, Berry A, Ghosn N, Bizri AR. Revisiting leishmaniasis in the time of war: the Syrian conflict and the Lebanese outbreak. International Journal of Infectious Diseases. 2014;29:115-9.

221. Amr ZS, Kanani K, Shadfan B, Hani RB. Cutaneous leishmaniasis among Syrian refugees in Jordan: a Retrospective Study. Bull Soc Pathol Exot. 2018;111(5):295-300.

222. Badiaga S, Brouqui P, Raoult D. Autochthonous epidemic typhus associated with Bartonella quintana bacteremia in a homeless person. m J Trop Med Hyg. 2005;72(5):638-9.

223. Bizri NA, Alam W, Khoury M, Musharrafieh U, Ghosn N, Berri A, et al. The association Between the Syrian crisis and cutaneous leishmaniasis in Lebanon. Acta Parasitologica. 2021:1-6.

224. Bustamante J, Sainz T, Ara-Montojo MF, Almiron MD, Subirats M, Vega DM, et al. Screening for parasites in migrant children. Travel Medicine and Infectious Disease. 2022;47. doi:10.1016/j.tmaid.2022.102287.

225. Bustamante J, Sainz T, Perez S, Rodriguez-Molino P, Vega DM, Mellado MJ, et al. Toxocariasis in migrant children: A 6 years' experience in a reference pediatric unit in Spain. Travel Medicine and Infectious Disease. 2022;47. doi:10.1016/j.tmaid.2022.102288.

226. Chaves NJ, Gibney KB, Leder K, O'Brien DP, Marshall C, Biggs BA. Screening practices for infectious diseases among Burmese refugees in Australia. Emerg Infect Dis. 2009;15(11):1769-72. doi: 10.3201/eid1511.090777.

227. De Vetten G, Dirksen M, Weaver R, Turin T, Aucoin MW. Parasitic stool testing in newly arrived refugees in Calgary, Alta. Canadian Family Physician. 2017;63(12):e518-e25.

228. Desai AN, Ramatowski JW, Marano N, Madoff LC, Lassmann B. Infectious disease outbreaks among forcibly displaced persons: An analysis of ProMED reports 1996-2016. Emerg Infect Dis. 2020;14(1).

229. Doganay M, Demiraslan H. Refugees of the Syrian Civil War: Impact on reemerging infections, health services, and biosecurity in Turkey. Health Secur. 2016;14(4):220-5.

230. Dorkenoo MA, Tchankoni MK, Yehadji D, Yakpa K, Tchalim M, Sossou E, et al. Monitoring migrant groups as a post-validation surveillance approach to contain the potential reemergence of lymphatic filariasis in Togo. Parasites & Vectors. 2021;14(1).

231. El Safadi D, Merhabi S, Rafei R, Mallat H, Hamze M, Acosta-Serrano A. Cutaneous leishmaniasis in north Lebanon: Re-emergence of an important neglected tropical disease. Trans R Soc Trop Med Hyg. 2019;113(8):471-6.

232. Hussain M, Munir S, Jamal MA, Ayaz S, Akhoundi M, Mohamed K. Epidemic outbreak of anthroponotic cutaneous leishmaniasis in Kohat District, Khyber Pakhtunkhwa, Pakistan. Acta Tropica. 2017;172:147-55.

233. Kanani K, Amr ZS, Shadfan B, Khorma R, Rø G, Abid M, et al. Cutaneous leishmaniasis among Syrian refugees in Jordan. Acta Tropica. 2019;194:169-71.

234. Kheirallah KA, Ababneh BF, Bendak H, Alsuwaidi AR, Elbarazi I. Exploring the mental, social, and lifestyle effects of a positive COVID-19 infection on Syrian refugees in Jordan: A qualitative study. International Journal of Environmental Research and Public Health. 2022;19(19). doi:10.3390/ijerph191912588.

235. Kondilis E, Papamichail D, McCann S, Carruthers E, Veizis A, Orcutt M, et al. The impact of the COVID-19 pandemic on refugees and asylum seekers in Greece: A retrospective analysis of national surveillance data from 2020. EClinicalMedicine. 2021;37. doi:10.1016/j.eclinm.2021.100958.

236. Lucchini A, Lipani F, Costa C, Scarvaglieri M, Balbiano R, Carosella S, et al. Louseborne relapsing fever among East African refugees, Italy, 2015. Emerg Infect Dis. 2016;22(2):298-301.

237. Ly TDA, Dao TL, Hoang VT, Braunstein D, Brouqui P, Lagier JC, et al. Pattern of infections in French and migrant homeless hospitalised at Marseille infectious disease units, France: A retrospective study, 2017–2018. Travel Med Infect Dis. 2020;36.

238. O'Neal SE, Townes JM, Wilkins PP, Noh JC, Lee D, Rodriguez S, et al. Seroprevalence of antibodies against Taenia solium cysticerci among refugees resettled in United States. Emerg Infect Dis. 2012;18(3):431-8.

239. Yentur Doni N, Gurses G, Dikme R, Aksoy M, Yildiz Zeyrek F, Simsek Z, et al. Cutaneous leishmaniasis due to three Leishmania species among Syrian refugees in Sanliurfa, Southeastern Turkey. Acta Parasitol. 2020;65(4):936-48.

240. Zambrano LD, Samson O, Phares C, Jentes E, Weinberg M, Goers M, et al. Unresolved splenomegaly in recently resettled Congolese refugees - multiple states, 2015-2018. MMWR. 2018;67(49):1358-62.
