# Supplementary material for: Status of zoonotic disease research in refugees, asylum seekers and internally displaced people, globally: A scoping review of forty clinically important zoonotic pathogens
Source: PLoS Negl Trop Dis. 2024 May 20;18(5):e0012164. doi: 10.1371/journal.pntd.0012164 (PMC11142688; doi:10.1371/journal.pntd.0012164)
Supplement: S7 Table — (DOCX) [file pntd.0012164.s009.docx]

**S7 Table: Publications included in the scoping review reporting migration routes of displaced people, by first year of sample collection**

| Initial sample collection year | References |
| --- | --- |
| 1937 – 1979 | [1-12] |
| 1980 – 1989 | [13-43] |
| 1990 – 1999 | [44-68] |
| 2000 – 2009 | [69-119] |
| 2010 – 2019 | [120-222] |
| 2020 – 2022 | [223-252] |

**References**

1. Arfaa F. Intestinal parasites among Indochinese refugees and Mexican immigrants resettled in Contra Costa County, California. J Fam Pract. 1981;12(2):223-6.

2. Barbier D, Demenais F, Lefait JF, David B, Blanc M, Hors J, et al. Susceptibility to human cutaneous leishmaniasis and HLA, Gm, Km markers. Tissue Antigens. 1987;30(2):63-7.

3. Espinel CH. A piece of my mind. On the trail of color. JAMA. 1996;275(3):168.

4. Francke E. Medical evaluation of Indochinese refugees conditions to consider. Postgraduate Medicine. 1982;72(5):92-3.

5. Harris AR, Russell RJ, Charters AD. A review of schistosomiasis in immigrants in Western Australia, demonstrating the unusual longevity of Schistosoma mansoni. Trans R Soc Trop Med Hyg. 1984;78(3):385-8.

6. Hoffman SL, Barrett-Connor E, Norcross W, Nguyen D. Intestinal parasites in Indochinese immigrants. Am J Trop Med Hyg. 1981;30(2):340-3.

7. Jones MJ, Thompson Jr JH, Brewer NS. Infectious diseases of Indochinese refugees. Mayo Clin Proc. 1980;55(8):482-8.

8. Stehr-Green JK, Schantz PM. Trichinosis in Southeast Asian refugees in the United States. Am J Public Health. 1986;76(10):1238-9.

9. Sullivan R, Linneman Jr CC, Clark CS, Walzer PD. Seroepidemiologic study of giardiasis patients and high-risk groups in a midwestern city in the United States. Am J Public Health. 1987;77(8):960-3.

10. Taylor R. Typhoid fever in the Basque Refugee Camp. British Medical Journal. 1937;1937:760-1.

11. Temcharoen P, Viboolyavatana J, Tongkoom B. A survey on intestinal parasitic infections in Laotian refugees at Ubon Province, northeastern Thailand, with special reference to schistosomiasis. Southeast Asian J Trop Med Public Health. 1979;10(4):552-5.

12. Wiesenthal AM, Nickels MK, Hashimoto KG. Intestinal parasites in Southeast-Asian refugees. Prevalence in a community of Laotians. JAMA. 1980;244(22):2543-4.

13. Enterically transmitted non-A, non-B hepatitis--East Africa. MMWR. 1987;36(16):241-4.

14. Arthur JD, Bodhidatta L, Echeverria P, Phuphaisan S, Paul S. Diarrheal disease in Cambodian children at a camp in Thailand. Am J Epidemiol. 1992;135(5):541-51.

15. Berger SA, Schwartz T, Michaeli D. Infectious disease among Ethiopian immigrants in Israel. Arch Intern Med. 1989;149(1):117-9.

16. Brown AE, Meek SR, Maneechai N, Lewis GE. Murine typhus among Khmers living at an evacuation site on the Thai-Kampuchean border. Am J Trop Med Hyg. 1988;38(1):168-71.

17. Brown V, Larouze B, Desve G, Rousset JJ, Thibon M, Fourrier A, et al. Clinical presentation of louse-born relapsing fever among Ethiopian refugees in northern Somalia. Ann Trop Med Parasitol. 1988;82(5):499-502.

18. D'Alauro F, Lee RV, Pao-In K, Khairallah M. Intestinal parasites and pregnancy. Infect Dis Obstet Gynecol. 1985;66(5):639-43.

19. Dao AH, Gregory DW, McKee LC. Specific health problems of Southeast Asian refugees in middle Tennessee. Southern Medical Journal. 1984;77(8):995-7.

20. DeGirolami PC, Kimber J. Intestinal parasites among Southeast Asian refugees in Massachusetts. Am J Clin Pathol. 1983;79(4):502-4.

21. Duffy PE, Le Guillouzic H, Gass RF, Innis BL. Murine typhus identified as a major cause of febrile illness in a camp for displaced Khmers in Thailand. Am J Trop Med Hyg. 1990;43(5):520-6.

22. Fritzsche M, Gottstein B, Wigglesworth MC, Eckert J. Serological survey of human cysticercosis in Irianese refugee camps in Papua New Guinea. Acta Tropica. 1990;47(2):69-77.

23. Godue CB, Gyorkos TW. Intestinal parasites in refugee claimants: a case study for selective screening? Can J Public Health. 1990;81(3):191-5.

24. Gyorkos TW, Frappier-Davignon L, MacLean JD, Viens P. Effect of screening and treatment on imported intestinal parasite infections: Results from a randomized, controlled trial. Am J Epidemiol. 1989;129(4):753-61.

25. Gyorkos TW, MacLean JD, Viens P, Chheang C, Kokoskin-Nelson E. Intestinal parasite infection in the Kampuchean refugee population 6 years after resettlement in Canada. Infect Dis. 1992;166(2):413-7.

26. Kjersem H, Jepsen S, Larsen L, Black F. Salmonella and Shigella carriers among refugees from the Middle East and Sri Lanka in Denmark. Scandinavian Journal of Social Medicine. 1990;18(3):175-8.

27. Lerman D, Barrett-Connor E, Norcross W. Intestinal parasites in asymptomatic adult Southeast Asian immigrants. J Fam Pract. 1982;15(3):443-6.

28. Parenti DM, Lucas D, Lee A, Hollenkamp RH. Health status of Ethiopian refugees in the United States. Am J Public Health. 1987;77(12):1542-3.

29. Parish RA. Intestinal parasites in Southeast Asian refugee children. West J Med. 1985;143(1):47-9.

30. Perea WA, Ancelle T, Moren A, Nagelkerke M, Sondorp E. Visceral leishmaniasis in southern Sudan. Trans R Soc Trop Med Hyg. 1991;85(1):48-53.

31. Schroeder Jr HW, Yarrish RL, Perkins TF, Lee C. Sequential disseminated tuberculosis and toxoplasmosis in a Haitian refugee. Southern Medical Journal. 1984;77(4):533-4.

32. Taylor DN, Echeverria P, Pitarangsi C, Seriwatana J, Sethabutr O, Bodhidatta L, et al. Application of DNA hybridization techniques in the assessment of diarrheal disease among refugees in Thailand. Am J Epidemiol. 1988;127(1):179-87.

33. Yangco BG, Vincent AL, Vickery AC. A survey of filariasis among refugees in South Florida. Am J Trop Med Hyg. 1984;33(2):246-51.

34. Hershko C, Nesher G, Yinnon AM. Medical problems in Ethiopian refugees airlifted to Israel: Experience in 131 patients admitted to a general hospital. J Trop Med Hyg. 1986;89(3):107-12.

35. de Beer P, el Harith A, van Grootheest M, Winkler A. Outbreak of kala-azar in the Sudan. Lancet. 1990;335(8683):224. doi: 10.1016/0140-6736(90)90313-t.

36. IsaÃ¤cson M, Frean J, He J, Seriwatana J, Innis BL. An outbreak of hepatitis E in Northern Namibia, 1983. Am J Trop Med Hyg. 2000;62(5):619-25. doi: 10.4269/ajtmh.2000.62.619.

37. Lurio J, Verson H, Karp S. Intestinal parasites in Cambodians: comparison of diagnostic methods used in screening refugees with implications for treatment of populations with high rates of infestation. J Am Board Fam Pract. 1991;4(2):71-8.

38. McDowell D, Harper CG. Neurocysticercosis - Two Australian cases. Med J Aust. 1990;152(4):217-8.

39. Persson A, Rombo L. Intestinal parasites in refugees and asylum seekers entering the Stockholm area, 1987-88: Evaluation of routine stool screening. Scand J Infect Dis. 1994;26(2):199-207.

40. Ryan N, Plackett M, Dwyer B. Parasitic infections of refugees. Medical Journal of Australia. 1988;148(10):491-4. doi:10.5694/j.1326-5377.1988.tb99455.x.

41. Samuda GM, Chan SP, Yeung CY. Vietnamese child health in a Hong Kong closed camp. Aust Paediatr J. 1988;24(2):115-7.

42. Southwood T, Davidson GP, Phillips GE, Rice M. Hepatosplenic schistosomiasis in a South-East Asian refugee child in South Australia. Aust N Z J Med. 1983;13(4):384-6.

43. Zijlstra EE, Siddig Ali M, El-Hassan AM, El-Toum IA, Satti M, Ghalib HW, et al. Kala-azar in displaced people from southern Sudan: Epidemiological, clinical and therapeutic findings. Trans R Soc Trop Med Hyg. 1991;85(3):365-9.

44. Benzeguir AK, Capraru T, Aust-Kettis A, Björkman A. High frequency of gastrointestinal parasites in refugees and asylum seekers upon arrival in Sweden. Scand J Infect Dis. 1999;31(1):79-82.

45. Bradarić N, Punda-Polić V, Milas I, Ivić I, Grgić D, Radosević N, et al. Two outbreaks of typhoid fever related to the war in Bosnia and Herzegovina. European Journal of Epidemiology. 1996;12(4):409-12.

46. Chalupa P, Vanista J, Burget I, Stary J, Sukova M, Nohynkova M. The review of imported visceral leishmaniosis in the Czech Republic. Bratislavské lekárske listy. 2001;102(2):84-91.

47. Chironna M, Germinario C, Lupalco PL, Carrozzini F, Quarto M. Prevalence of hepatitis virus infections in Kosovar refugees. International Journal of Infectious Diseases. 2001;5(4):209-13.

48. Geltman PL, Cochran J, Hedgecock C. Intestinal parasites among African refugees resettled in Massachusetts and the impact of an overseas pre-departure treatment program. Am J Trop Med Hyg. 2003;69(6):657-62.

49. Jensenius M, Hoiby EA, Berild D, Stiris M, Ringertz SH. Difficulties in Diagnosing Brucella spondylitis. Scand J Infect Dis. 2000;32(4):425-6.

50. Kolaczinski J, Brooker S, Reyburn H, Rowland M. Epidemiology of anthroponotic cutaneous leishmaniasis in Afghan refugee camps in northwest Pakistan. Transactions of the Royal Society of Tropical Medicine and Hygiene. 2004;98(6):373-8.

51. Lifson AR, Thai D, O'Fallon A, Mills WA, Hang K. Prevalence of tuberculosis, hepatitis B virus, and intestinal parasitic infections among refugees to Minnesota. Public Health Rep. 2002;117(1):69-77.

52. Malamitsi-Puchner A, Papacharitonos S, Sotos D, Tzala L, Psichogiou M, Hatzakis A, et al. Prevalence study of different hepatitis markers among pregnant Albanian refugees in Greece. European Journal of Epidemiology. 1996;12(3):297-301.

53. McAuley JB, Michelson MK, Hightower AW, Engeran S, Wintermeyer LA, Schantz PM. A trichinosis outbreak among Southeast Asian refugees. Am J Epidemiol. 1992;135(12):1404-10.

54. Meropol SB. Health status of pediatric refugees in Buffalo, NY. Arch Pediatr Adolesc Med. 1995;149(8):887-92.

55. Miller JM, Boyd HA, Ostrowski SR, Cookson ST, Parise ME, Gonzaga PS, et al. Malaria, intestinal parasites, and schistosomiasis among Barawan Somali refugees resettling to the United States: A strategy to reduce morbidity and decrease the risk of imported infections. Am J Trop Med Hyg. 2000;62(1):115-21.

56. Moaven L, Van Asten M, Crofts N, Locarnini SA. Seroepidemiology of hepatitis E in selected Australian populations. J Med Virol. 1995;45(3):326-30.

57. Mumtaz K, Kamani L, Chawla T, Hamid S, Jafri W. Hepatic cystic echinococcosis: clinical characteristics and outcomes in Pakistan. Trop Doct. 2009;39(4):215-7.

58. Ofoezie IE, Asaulu SO, Christensen NØ, Madsen H. Patterns of infection with Schistosoma haematobium in lakeside resettlement communities at the Oyan Reservoir in Ogun State, south-western Nigeria. Ann Trop Med Parasitol. 1997;91(2):187-97.

59. Raoult D, Ndihokubwayo JB, Tissot-Dupont H, Roux V, Faugere B, Abegbinni R, et al. Outbreak of epidemic typhus associated with trench fever in Burundi. The Lancet. 1998;352(9125):353-8.

60. Rowland M, Munir A, Durrani N, Noyes H, Reyburn H. An outbreak of cutaneous leishmaniasis in an Afghan refugee settlement in north-west Pakistan. J Transactions of the Royal Society of Tropical Medicine and Hygiene. 1999;93(2):133-6.

61. Sencan I, Sahin I, Kaya D, Oksuz S, Yildirim M. Assessment of HAV and HEV seroprevalence in children living in post-earthquake camps from Düzce, Turkey. 2004;19(5):461-5.

62. Seybolt LM, Christiansen D, Barnett ED. Diagnostic evaluation of newly arrived asymptomatic refugees with eosinophilia. Clinical Infectious Diseases. 2006;42(3):363-7.

63. Swanson SJ, Phares CR, Mamo B, Smith KE, Cetron MS, Stauffer WM. Albendazole therapy and enteric parasites in United States-bound refugees. 2012;366(16):1498-507.

64. Watts NS, Mizinduko MM, Barnett ED, White LF, Hochberg NS. Association between parasitic infections and tuberculin skin test results in refugees. Travel Medicine and Infectious Disease. 2017;16:35-40.

65. Zijlstra EE, Ali MS, El-Hassan AM, El-Toum IA, Satti M, Ghalib Kager HWPA. Direct agglutination test for diagnosis and sero- epidemiological survey of kala-azar in the Sudan. Trans R Soc Trop Med Hyg. 1991;85(4):474-6.

66. (CDC) CfDCaP. Imported dracunculiasis--United States, 1995 and 1997. MMWR. 1998;47(11):209-11.

67. Ekdahl K, Andersson Y. Imported giardiasis: Impact of international travel, immigration, and adoption. Am J Trop Med Hyg. 2005;72(6):825-30.

68. Gray GC, Rodier GR, Matras-Maslin VC, Honein MA, Ismail EA, Botros BA, et al. Serologic evidence of respiratory and rickettsial infections among Somali refugees. Am J Trop Med Hyg. 1995;52(4):349-53. doi: 10.4269/ajtmh.1995.52.349.

69. Hepatitis E, Chad. MMWR. 2004;79(35):313.

70. Haq KUA, Gul NA, Hammad HM, Bibi Y, Bibi A, Mohsan J. Prevalence of Giardia intestinalis and Hymenolepis nana in Afghan refugee population of Mianwali district, Pakistan. Afr Health Sci. 2015;15(2):394-400.

71. Alawieh A, Musharrafieh U, Jaber A, Berry A, Ghosn N, Bizri AR. Revisiting leishmaniasis in the time of war: the Syrian conflict and the Lebanese outbreak. International Journal of Infectious Diseases. 2014;29:115-9.

72. Benson J. Asymptomatic schistosomiasis in a young Sudanese refugee. Australian Family Physician. 2007;36(4):249-51.

73. Bizri NA, Alam W, Khoury M, Musharrafieh U, Ghosn N, Berri A, et al. The association between the Syrian crisis and cutaneous leishmaniasis in Lebanon. Acta Parasitologica. 2021:1-6.

74. Bloch-Infanger C, Bättig V, Kremo J, Widmer AF, Egli A, Bingisser R, et al. Increasing prevalence of infectious diseases in asylum seekers at a tertiary care hospital in Switzerland. PLoS One. 2017;12(6):e0179537.

75. Boccia D, Guthmann JP, Klovstad H, Hamid N, Tatay M, Ciglenecki I, et al. High mortality associated with an outbreak of hepatitis E among displaced persons in Darfur, Sudan. Clinical Infectious Diseases. 2006;42(12):1679-84.

76. Brodine SK, Thomas A, Huang R, Harbertson J, Mehta S, Leake J, et al. Community based parasitic screening and treatment of sudanese refugees: Application and assessment of centers for disease control guidelines. Am J Trop Med Hyg. 2009;80(3):425-30.

77. Brooker S, Mohammed N, Adil K, Agha S, Reithinger R, Rowl, et al. Leishmaniasis in refugee and local Pakistani populations. Emerg Infect Dis. 2004;10(9):1681-4.

78. Ceccarelli G, d'Ettorre G, Riccardo F, Ceccarelli C, Chiaretti M, Picciarella A, et al. Urinary schistosomiasis in asylum seekers in Italy: an emergency currently undervalued. J Immigr Minor Health. 2013;15(4):846-50.

79. Chandrasena TGAN, Hapuarachchi HC, Dayanath MYD, Pathmeswaran A, De Silva NR. Intestinal parasites and the growth status of internally displaced children in Sri Lanka. Trop Doct. 2007;37(3):163-5.

80. Chang AH, Perry S, Du JNT, Agunbiade A, Polesky A, Parsonnet J. Decreasing intestinal parasites in recent northern California refugees. Am J Trop Med Hyg. 2013;88(1):191-7.

81. Cherian P, Junckerstorff RK, Rosen D, Kumarasinghe P, Morling A, Tuch P, et al. Late-stage human African trypanosomiasis in a Sudanese refugee. Medical Journal of Australia. 2010;192(7):417-9.

82. Chironna M, Germinario C, Lopalco PL, Carrozzini F, Barbuti S, Quarto M. Prevalence rates of viral hepatitis infections in refugee Kurds from Iraq and Turkey. Infection. 2003;31(2):70-4.

83. Crogan J, Gunasekera H, Wood N, Sheikh M, Isaacs D. Management of old world cutaneous leishmaniasis in refugee children. Pediatr Infect Dis J. 2010;29(4):357-9.

84. Dawson-Hahn EE, Greenberg SLM, Domachowske JB, Olson BG. Eosinophilia and the seroprevalence of Schistosomiasis and Strongyloidiasis in newly arrived pediatric refugees: An examination of centers for disease control and prevention screening guidelines. Journal of Pediatrics. 2010;156(6):1016-U194.

85. Fan CK, Liao CW, Wu MS, Su KE, Han BC. Seroepidemiology of Toxoplasma gondii infection among Chinese aboriginal and Han people residing in mountainous areas of northern Thailand. J Parasitol. 2003;89(6):1239-42.

86. Franco-Paredes C, Dismukes R, Nicolls D, Hidron A, Workowski K, Rodriguez-Morales A, et al. Persistent and untreated tropical infectious diseases among Sudanese refugees in the United States. Am J Trop Med Hyg. 2007;77(4):633-5.

87. Goswami ND, Shah JJ, Corey GR, Stout JE. Short report: Persistent eosinophilia and Strongyloides infection in Montagnard refugees after presumptive albendazole therapy. Am J Trop Med Hyg. 2009;81(2):302-4.

88. Khan MI, Muhammad M, Khan W, Khan N, Noor SM. Nasal involvement in cutaneous leishmaniasis. Journal of Postgraduate Medical Institute. 2010;24(3):202-6.

89. Consultative Group for RVF Decision Support. Decision-support tool for prevention and control of Rift Valley fever epizootics in the Greater Horn of Africa. Am J Trop Med Hyg. 2010;83(2 Suppl):75-85. doi: 10.4269/ajtmh.2010.83s2a03.

90. Lin CY, Chen TC, Dai CY, Yu ML, Lu PL, Yen JH, et al. Serological investigation to identify risk factors for post-flood infectious diseases: a longitudinal survey among people displaced by Typhoon Morakot in Taiwan. BMJ Open. 2015;5(5):e007008.

91. Marlet MVL, Wuillaume F, Jacquet D, Quispe KW, Dujardin JC, Boelaert M. A neglected disease of humans: A new focus of visceral leishmaniasis in Bakool, Somalia. Trans R Soc Trop Med Hyg. 2003;97(6):667-71.

92. Martin JA, Mak DB. Changing faces: a review of infectious disease screening of refugees by the Migrant Health Unit, Western Australia in 2003 and 2004. Medical Journal of Australia. 2006;185(11):607-10.

93. Masters PJ, Lanfranco PJ, Sneath E, Wade AJ, Huffam S, Pollard J, et al. Health issues of refugees attending an infectious disease refugee health clinic in a regional Australian hospital. Australian Journal of General Practice. 2018;47(5):305-10.

94. Mérens A, Guérin PJ, Guthmann JP, Nic, E. Outbreak of hepatitis E virus infection in Darfur, Sudan: Effectiveness of real-time reverse transcription-PCR analysis of dried blood spots. J Clin Microbiol. 2009;47(6):1931-3.

95. Miladinovic-Tasic NL, Tasic SA, Kranjcic-Zec I, Tasic G, Tasic A, Tasic IS. Asymptomatic giardiasis-more prevalent in refugees than in native inhabitants of the city of Nis, Serbia. Central European Journal of Medicine. 2008;3(2):203-6.

96. Mutch RC, Cherian S, Nemba K, Geddes JS, Rutherford DM, Chaney GM, et al. Tertiary paediatric refugee health clinic in Western Australia: Analysis of the first 1026 children. Journal of Paediatrics and Child Health. 2012;48(7):582-7.

97. Nicand E, Armstrong GL, Enouf V, Guthmann JP, Guerin JP, Caron M, et al. Genetic heterogeneity of hepatitis E virus in Darfur, Sudan, and neighboring Chad. Journal of Medical Virology. 2005;77(4):519-21.

98. Ntais P, Christodoulou V, Tsirigotakis N, Dokianakis E, Dedet J-P, Pratlong F, et al. Will the introduction of Leishmania tropica MON-58, in the island of Crete, lead to the settlement and spread of this rare zymodeme? Acta Tropica. 2014;132:125-30. doi:10.1016/j.actatropica.2014.01.003.

99. O'Neal SE, Townes JM, Wilkins PP, Noh JC, Lee D, Rodriguez S, et al. Seroprevalence of antibodies against Taenia solium cysticerci among refugees resettled in United States. Emerg Infect Dis. 2012;18(3):431-8.

100. Paxton GA, Sangster KJ, Maxwell EL, McBride CRJ, Drewe RH. Post-arrival health screening in Karen refugees in Australia. PLoS One. 2012;7(5).

101. Posey DL, Blackburn BG, Weinberg M, Flagg EW, Ortega L, Wilson M, et al. High prevalence and presumptive treatment of schistosomiasis and strongyloidiasis among African refugees. Clinical Infectious Diseases. 2007;45(10):1310-5.

102. Raman S, Wood N, Webber M, Taylor KA, Isaacs D. Matching health needs of refugee children with services: how big is the gap? Aust N Z J Public Health. 2009;33(5):466-70.

103. Sheikh M, Pal A, Wang S, MacIntyre CR, Wood NJ, Isaacs D, et al. The epidemiology of health conditions of newly arrived refugee children: A review of patients attending a specialist health clinic in Sydney. Journal of Paediatrics and Child Health. 2009;45(9):509-13.

104. Shen C, Li S, Zheng S, Choi MH, Bae YM, Hong ST. Tissue parasitic helminthiases are prevalent at Cheongjin, North Korea. Korean J Parasitol. 2007;45(2):139-44.

105. Tappe D, Weise D, Ziegler U, Müller A, Müllges W, Stich A. Brain and lung metastasis of alveolar echinococcosis in a refugee from a hyperendemic area. J Med Microbiol. 2008;57(11):1420-3.

106. Tiong ACD, Patel MS, Gardiner J, Ryan R, Linton KS, Walker KA, et al. Health issues in newly arrived African refugees attending general practice clinics in Melbourne. Medical Journal of Australia. 2006;185(11):602-6.

107. Ul Haq KA, Gul NA, Muhammad Hammad H, Bibi Y, Bibi A, Mohsan J. Prevalence of giardia intestinalis and hymenolepis nana in afghan refugee population of mianwali district, pakistan. African Health Sciences. 2015;15(2):394-400.

108. Wollina U, Koch A, Guarneri C, Tchernev G, Lotti T. Cutaneous leishmaniasis – A case series from Dresden. Open Access Maced J Med Sci. 2018;6(1):89-92.

109. Abu Mourad TA. Palestinian refugee conditions associated with intestinal parasites and diarrhoea: Nuseirat refugee camp as a case study. Public Health. 2004;118(2):131-42.

110. Johnston V, Smith L, Roydhouse H. The health of newly arrived refugees to the top end of Australia: Results of a clinical audit at the Darwin refugee health service. BMJ Open. 2012;18(3):242-7.

111. Lemieux A, Lagacé F, Billick K, Ndao M, Yansouni CP, Semret M, et al. Cutaneous leishmaniasis in travellers and migrants: a 10-year case series in a Canadian reference centre for tropical diseases. CMAJ open. 2022;10(2):E546-E53. doi:10.9778/cmajo.20210238.

112. Rahim M, Kazi BM, Bile KM, Munir M, Khan AR. The impact of the disease early warning system in responding to natural disasters and conflict crises in Pakistan. Eastern Mediterranean Health Journal. 2010;16:S114-21. doi:10.26719/2010.16.supp.114.

113. Bedard B, Pennise M, Weimer AC, Kennedy BS. Magnitude of Giardia cases among refugees, adoptees and immigrants in Monroe County, New York, 2003-2013. International Journal of Migration, Health and Social Care. 2016;12(3):211-5.

114. Caruana SR, Kelly HA, Ngeow JYY, Ryan NJ, Bennett CM, Chea L, et al. Undiagnosed and potentially lethal parasite infections among immigrants and refugees in Australia. Journal of Travel Medicine. 2006;13(4):233-9.

115. Chaves NJ, Gibney KB, Leder K, O'Brien DP, Marshall C, Biggs BA. Screening practices for infectious diseases among Burmese refugees in Australia. Emerg Infect Dis. 2009;15(11):1769-72. doi: 10.3201/eid1511.090777.

116. Guthmann JP, Klovstad H, Boccia D, Hamid N, Pinoges L, Nizou JY, et al. A large outbreak of hepatitis E among a displaced population in Darfur, Sudan, 2004: the role of water treatment methods. Clin Infect Dis. 2006;42(12):1685-91. doi: 10.1086/504321.

117. Teshale EH, Grytdal SP, Howard C, Barry V, Kamili S, Drobeniuc J, et al. Evidence of person-to-person transmission of hepatitis E virus during a large outbreak in Northern Uganda. Clinical Infectious Diseases. 2010;50(7):1006-10. doi: 10.1086/651077.

118. Zwi K, Morton N, Woodland L, Mallitt K-A, Palasanthiran P. Screening and primary care access for newly arrived paediatric refugees in regional Australia: A 5 year cross-sectional analysis (2007–12). Journal of Tropical Pediatrics. 2016;63(2):109-17. doi: 10.1093/tropej/fmw059.

119. Quandelacy TM, Riefkohl A, Franco-Paredes C. Prevalence of untreated schistosomiasis among Sudanese refugees: “The Lost Boys of Sudan” in the United States. Boletin medico del Hospital Infantil de Mexico. 2010;67:503-6.

120. Ahmed W, Ahmad M, Rafatullah, Shah F, Sajadullah. Pervasiveness of intestinal protozoan and worm incursion in IDP's (North Waziristan agency, KPK-Pakistan) children of 6-16 years. Journal of the Pakistan Medical Association. 2015;65(9):943-5.

121. Alberer M, Malinowski S, Sanftenberg L, Schelling J. Notifiable infectious diseases in refugees and asylum seekers: experience from a major reception center in Munich, Germany. Infection. 2018;46(3):375-83.

122. Alhawarat M, Khader Y, Shadfan B, Kaplan N, Iblan I. Trend of cutaneous Leishmaniasis in Jordan from 2010 to 2016: Retrospective study. JMIR Public Health and Surveillance. 2020;6(1).

123. Aliskin O, Savas N. Notifiable communicable diseases in Turkey and their notification status: Antakya sample. Flora the Journal of Infectious Diseases and Clinical Microbiology. 2019;24(1):11-21.

124. Amr ZS, Kanani K, Shadfan B, Hani RB. Cutaneous leishmaniasis among Syrian refugees in Jordan: a retrospective study. Bull Soc Pathol Exot. 2018;111(5):295-300.

125. Angheben A, Mariconti M, Degani M, Gobbo M, Palvarini L, Gobbi F, et al. Is there echinococcosis in West Africa? A refugee from Niger with a liver cyst. Parasit Vectors. 2017;10(1).

126. Antinori S, Mediannikov O, Corbellino M, Gr, e R, Parravicini C, et al. Louse-borne relapsing fever (Borrelia recurrentis) in a Somali Refugee arriving in Italy: A re-emerging infection in Europe? PLoS Negl Trop Dis. 2016;10(5).

127. Antinori S, Mediannikov O, Corbellino M, Raoult D. Louse-borne relapsing fever among East African refugees in Europe. Travel Med Infect Dis. 2016;14(2):110-4.

128. Antinori S, Tonello C, Edouard S, Parravicini C, Gastaldi D, Gr, et al. Diagnosis of louse-borne relapsing fever despite negative microscopy in two asylum seekers from Eastern Africa. Am J Trop Med Hyg. 2017;97(6):1669-72.

129. Azman AS, Bouhenia M, Iyer AS, Rumunu J, Laku RL, Wamala JF, et al. High hepatitis e seroprevalence among displaced persons in South Sudan. Am J Trop Med Hyg. 2017;96(6):1296-301.

130. Browne LB, Menkir Z, Kahi V, Maina G, Asnakew S, Tubman M, et al. Hepatitis E outbreak among refugees from South Sudan - Gambella, Ethiopia, April 2014-January 2015. MMWR. 2015;64(19):537.

131. Buonfrate D, Gobbi F, Marchese V, Postiglione C, Monteiro GB, Giorli G, et al. Extended screening for infectious diseases among newly-arrived asylum seekers from Africa and Asia, Verona province, Italy, April 2014 to June 2015. Eurosurveillance. 2019;23(16):7-14.

132. Chen L, Peek M, Stokich D, Todd R, Anderson M, Murphy FK, et al. Japanese encephalitis in two children-United States, 2010. MMWR. 2011;60(9):276-8.

133. Chernet A, Kling K, Sydow V, Kuenzli E, Hatz C, Utzinger J, et al. Accuracy of diagnostic tests for Schistosoma mansoni infection in asymptomatic Eritrean refugees: Serology and point-of-care circulating cathodic antigen against stool microscopy. Clinical Infectious Diseases. 2017;65(4):568-74.

134. Çizmeci Z, Karakuş M, Karabela ŞN, Erdoğan B, Güleç N. Leishmaniasis in Istanbul; A new epidemiological data about refugee leishmaniasis. Acta Trop. 2019;195:23-7.

135. Çoşkun B, Gülümser Ç, Çoşkun B, Artuk C, Karaşahin KE. Impact of Syrian refugees on congenital TORCH infections screening in Turkey. J Obs and Gynae Research. 2020;46(7):1017-24.

136. Costescu Strachinaru DI, Cambier J, et-Yattara H, Konopnicki D. Relapsing fever in asylum seekers from Somalia arriving in Belgium in August 2015. Acta Clin Belg. 2016;71(5):353-5.

137. Darcis G, Hayette MP, Bontems S, Sauvage AS, Meuris C, Van Esbroeck M, et al. Louse-borne relapsing fever in a refugee from Somalia arriving in Belgium. J Travel Med. 2016;23(3):3.

138. De Vetten G, Dirksen M, Weaver R, Turin T, Aucoin MW. Parasitic stool testing in newly arrived refugees in Calgary, Alta. Canadian Family Physician. 2017;63(12):e518-e25.

139. Doganay M, Demiraslan H. Refugees of the Syrian civil war: Impact on reemerging infections, health services, and biosecurity in Turkey. Health Secur. 2016;14(4):220-5.

140. Dorkenoo MA, Tchankoni MK, Yehadji D, Yakpa K, Tchalim M, Sossou E, et al. Monitoring migrant groups as a post-validation surveillance approach to contain the potential reemergence of lymphatic filariasis in Togo. Parasites & Vectors. 2021;14(1).

141. Ehlkes L, George M, Knautz D, Burckhardt F, Jahn K, Vogt M, et al. Negligible import of enteric pathogens by newly-arrived asylum seekers and no impact on incidence of notified Salmonella and Shigella infections and outbreaks in Rhineland-Palatinate, Germany, January 2015 to May 2016. Euro Surveill. 2018;23(20):7-14.

142. Eksi F, Ozgoztasi O, Karsligil T, Saglam M. Genotyping Leishmania promastigotes isolated from patients with cutaneous leishmaniasis in south-eastern Turkey. Journal of International Medical Research. 2016;45(1):114-22.

143. El Hajj R, El Hajj H, Khalifeh I. Fatal visceral leishmaniasis caused by Leishmania infantum, Lebanon. Emerg Infect Dis. 2018;24(5):906-7.

144. El Safadi D, Merhabi S, Rafei R, Mallat H, Hamze M, Acosta-Serrano A. Cutaneous leishmaniasis in north Lebanon: Re-emergence of an important neglected tropical disease. Trans R Soc Trop Med Hyg. 2019;113(8):471-6.

145. Enabulele EE, Platt RN, Adeyemi E, Agbosua E, Aisien MSO, Ajakaye OG, et al. Urogenital schistosomiasis in Nigeria post receipt of the largest single praziquantel donation in Africa. Acta Tropica. 2021:105916.

146. Goldenberger D, Claas GJ, Bloch-Infanger C, Breidthardt T, Suter B, Martinez M, et al. Louse-borne relapsing fever (Borrelia recurrentis) in an Eritrean refugee arriving in Switzerland, August 2015. Euro Surveill. 2015;20(32):2-5.

147. Grecchi C, Zanotti P, Pontarelli A, Chiari E, Tomasoni LR, Gulletta M, et al. Louse-borne relapsing fever in a refugee from Mali. Infection. 2017;45(3):373-6.

148. Grunow R, Jacob D, Klee S, Schlembach D, Jackowski-Dohrmann S, Loenning-Baucke V, et al. Brucellosis in a refugee who migrated from Syria to Germany and lessons learnt, 2016. Eurosurveillance. 2016;21(31):5-8.

149. Gurses G, Ozaslan M, Zeyrek FY, Kilic IH, Doni NY, Karagoz ID, et al. Molecular identification of Leishmania spp. isolates causes cutaneous leishmaniasis (CL) in Sanliurfa Province, Turkey, where CL is highly endemic. Folia Microbiol (Praha). 63(3):353-9.

150. Halici-Ozturk F, Yakut K, Öcal FD, Erol A, Gökay S, Çağlar AT, et al. Seroprevalence of Toxoplasma gondii infections in Syrian pregnant refugee women in Turkey. European Journal of Obstetrics and Gynecology and Reproductive Biology. 2021;256:91-4.

151. Hassan AO, Mero WMS. Prevalence of intestinal parasites among displaced people living in displacement camps in duhok province/Iraq. Internet Journal of Microbiology. 2020;17(1).

152. Heudorf U, Karathana M, Krackhardt B, Huber M, Raupp P, Zinn C. Surveillance for parasites in unaccompanied minor refugees migrating to Germany in 2015. Gms Hygiene and Infection Control. 2016;11:3.

153. Hoch M, Wieser A, Löscher T, Margos G, Pürner F, Zühl J, et al. Louse-borne relapsing fever (Borrelia recurrentis) diagnosed in 15 refugees from northeast Africa: Epidemiology and preventive control measures, Bavaria, Germany, July to October 2015. Euro Surveill. 2015;20(42).

154. Hussain M, Munir S, Jamal MA, Ayaz S, Akhoundi M, Mohamed K. Epidemic outbreak of anthroponotic cutaneous leishmaniasis in Kohat District, Khyber Pakhtunkhwa, Pakistan. Acta Tropica. 2017;172:147-55.

155. Hytönen J, Khawaja T, Grönroos JO, Jalava A, Meri S, Oksi J. Louse-borne relapsing fever in Finland in two asylum seekers from Somalia. APMIS. 2017;125(1):59-62.

156. Inci R, Ozturk P, Mulayim MK, Ozyurt K, Alatas ET, Inci MF. Effect of the Syrian civil war on prevalence of cutaneous leishmaniasis in Southeastern Anatolia, Turkey. Medical Science Monitor. 2015;21:5.

157. Jablonka A, Solbach P, Wöbse M, Manns MP, Schmidt RE, Wedemeyer H, et al. Seroprevalence of antibodies and antigens against hepatitis A-E viruses in refugees and asylum seekers in Germany in 2015. European Journal of Gastroenterology and Hepatology. 2017;29(8):939-45.

158. Janda A, Eder K, Fressle R, Geweniger A, Diffloth N, Heeg M, et al. Comprehensive infectious disease screening in a cohort of unaccompanied refugee minors in Germany from 2016 to 2017: A cross-sectional study. PLoS Medicine. 2020;17(3).

159. Kanani K, Amr ZS, Shadfan B, Khorma R, Rø G, Abid M, et al. Cutaneous leishmaniasis among Syrian refugees in Jordan. Bull Soc Pathol Exot. 2019;194:169-71.

160. Keller C, Zumblick M, Streubel K, Eickmann M, Müller D, Kerwat M, et al. Hemorrhagic diathesis in Borrelia recurrentis infection imported to Germany. Emerg Infect Dis. 2016;22(5):917-9.

161. Kortas AZ, Polenz J, von Hayek J, Rüdiger S, Rottbauer W, Storr U, et al. Screening for infectious diseases among asylum seekers newly arrived in Germany in 2015: a systematic single-centre analysis. Public Health. 2017;153:1-8.

162. Lagare A, Ibrahim A, Ousmane S, Issaka B, Zaneidou M, Kadadé G, et al. Outbreak of hepatitis E virus infection in displaced persons camps in Diffa region, Niger, 2017. Am J Trop Med Hyg. 2018;99(4):1055-7.

163. Lindner AK, Richter J, Gertler M, Nikolaus M, Martinez GE, Muller K, et al. Cutaneous leishmaniasis in refugees from Syria: complex cases in Berlin 2015-2020. Journal of Travel Medicine. 2020;27(7):8.

164. Lucchini A, Lipani F, Costa C, Scarvaglieri M, Balbiano R, Carosella S, et al. Louseborne relapsing fever among East African refugees, Italy, 2015. Emerg Infect Dis. 2016;22(2):298-301.

165. Ly TDA, Dao TL, Hoang VT, Braunstein D, Brouqui P, Lagier JC, et al. Pattern of infections in French and migrant homeless hospitalised at Marseille infectious disease units, France: A retrospective study, 2017–2018. Travel Med Infect Dis. 2020;36.

166. Maaßen W, Wiemer D, Frey C, Kreuzberg C, Tannich E, Hinz R, et al. Microbiological screenings for infection control in unaccompanied minor refugees: The German Armed Forces Medical Service's experience. Military Medical Research volume. 2017;4(1).

167. McCleery EJ, Patchanee P, Pongsopawijit P, Chailangkarn S, Tiwananthagorn S, Jongchansittoe P, et al. Taeniasis among refugees living on Thailand–Myanmar border, 2012. Emerg Infect Dis. 2015;21(10):1824-6.

168. Mekonnen GK, Mengistie B, Sahilu G, Kloos H, Mulat W. Etiologies of diarrhea and drug susceptibility patterns of bacterial isolates among under-five year children in refugee camps in Gambella Region, Ethiopia: a case control study. BMC Infect Dis. 2019;19(1).

169. Mitchell T, Lee D, Weinberg M, Phares C, James N, Amornpaisarnloet K, et al. Impact of enhanced health interventions for United States-bound refugees: Evaluating best practices in migration health. Am J Trop Med Hyg. 2018;98(3):920-8.

170. Mockenhaupt FP, Barbre KA, Jensenius M, Larsen CS, Barnett ED, Stauffer W, et al. Profile of illness in syrian refugees: A geosentinel analysis, 2013 to 2015. Eurosurveillance. 2016;21(10).

171. Montour J, Lee D, Snider C, Jentes ES, Stauffer W. Absence of Loa loa microfilaremia among newly arrived congolese refugees in Texas. Am J Trop Med Hyg. 2017;97(6):1833-5.

172. Nyamusore J, Nahimana MR, Ngoc CT, Olu O, Isiaka A, Ndahindwa V, et al. Risk factors for transmission of Salmonella Typhi in Mahama refugee camp, Rwanda: a matched case-control study. Pan African Medical Journal. 2018;29:13.

173. Oboth P, Gavamukulya Y, Barugahare BJ. Prevalence and clinical outcomes of Plasmodium falciparum and intestinal parasitic infections among children in Kiryandongo refugee camp, mid-Western Uganda: A cross sectional study. BMC Infect Dis. 2019;19(1).

174. Osthoff M, Schibli A, Fadini D, Lardelli P, Goldenberger D. Louse-borne relapsing fever - report of four cases in Switzerland, June-December 2015. BMC Infect Dis. 2016;16(1).

175. Paran Y, Ben-Ami R, Orlev B, Halutz O, Elalouf O, Wasserman A, et al. Chronic schistosomiasis in African immigrants in Israel: Lessons for the non-endemic setting. Medicine (Baltimore). 2019;98(52).

176. Pohl C, Mack I, Schmitz T, Ritz N. The spectrum of care for pediatric refugees and asylum seekers at a tertiary health care facility in Switzerland in 2015. Eur J Pediatr. 2017;176(12):1681-7.

177. Qazi M, Weimer AC, Bedard BA, Kennedy BS. Q-fever in a refugee after exposure to a central New York State livestock farm. Annals of Tropical Medicine and Public Health. 2016;9(4):266-70.

178. Ravensbergen SJ, Lokate M, Cornish D, Kloeze E, Ott A, Friedrich AW, et al. High prevalence of infectious diseases and drug-resistant microorganisms in asylum seekers admitted to hospital; no carbapenemase producing Enterobacteriaceae until September 2015. PLoS One. 2016;11(5):e0154791.

179. Redditt VJ, Janakiram P, Graziano D, Rashid M. Health status of newly arrived refugees in Toronto, Ont: Part 1: infectious diseases. Can Fam Physician. 2015;61(7):e303-e9.

180. Relić T, Kačarević H, Ilić N, Jovanović D, Tambur Z, Doder R, et al. Intestinal parasitosis in asylum seekers from the middle east and South Asia. Vojnosanitetski pregled. 2018;75(11):1101-5.

181. Richter J, Esmann L, Lindner AK, Trebesch I, Equihua-Martinez G, Niebank M, et al. Cystic echinococcosis in unaccompanied minor refugees from Afghanistan and the Middle East to Germany, July 2016 through June 2017. European Journal of Epidemiology. 2019;34(6):611-2.

182. Rodríguez-Morales AJ, Bonilla-Aldana DK, Bonilla-Aldana JC, Mondragón-Cardona Á. Arboviral diseases among internally displaced people of Neiva, Colombia, 2015-2017. Travel Med Infect Dis. 2019;26(2).

183. Saroufim M, Charafeddine K, Issa G, Khalifeh H, Habib RH, Berry A, et al. Ongoing Epidemic of Cutaneous Leishmaniasis among Syrian Refugees, Lebanon. Emerg Infect Dis. 2014;20(10):1712-5.

184. Sulaiman AA, Elmadhoun WM, Noor SK, Bushara SO, Almobarak AO, Awadalla H, et al. An outbreak of cutaneous leishmaniasis among a displaced population in North Sudan: Review of cases. J Family Med Prim Care. 2019;8(2):556-63.

185. Sulekova LF, Ceccarelli G, Pombi M, Esvan R, Lopalco M, Vita S, et al. Occurrence of intestinal parasites among asylum seekers in Italy: A cross-sectional study. Travel Med Infect Dis. 2018;27:46-52.

186. Theuring S, Friedrich-Janicke B, Portner K, Trebesch I, Durst A, Dieckmann S, et al. Screening for infectious diseases among unaccompanied minor refugees in Berlin, 2014-2015. Eur J Epidemiol. 2016;31(7):707-10.

187. Thomson K, Luis Dvorzak J, Lagu J, Laku R, Dineen B, Schilperoord M, et al. Investigation of hepatitis E outbreak among refugees - Upper Nile, South Sudan, 2012-2013. MMWR. 2013;62(29):581-6.

188. Um J, Nam Y, Lim JN, Kim M, An Y, Hwang SH, et al. Seroprevalence of scrub typhus, murine typhus and spotted fever groups in North Korean refugees. International Journal of Infectious Diseases. 2021;106:23-8.

189. Van Enter BJD, Lau YL, Ling CL, Watthanaworawit W, Sukthana Y, Lee WC, et al. Seroprevalence of toxoplasma gondii infection in refugee and migrant pregnant women along the Thailand-myanmar border. Am J Trop Med Hyg. 2017;97(1):232-5.

190. Van Kesteren L, Maniewski U, Bottieau E, Cnops L, Huits R. Cutaneous leishmaniasis in syrian refugee children: A case series. Pediatr Infect Dis J. 2020:E154-E6.

191. Williams B, Boullier M, Cricks Z, Ward A, Naidoo R, Williams A, et al. Screening for infection in unaccompanied asylum-seeking children and young people. Archives of Disease in Childhood. 2020;105(6):530-2.

192. Wilting KR, Stienstra Y, Sinha B, Braks M, Cornish D, Grundmann H. Louse-borne relapsing fever (Borrelia recurrentis) in asylum seekers from Eritrea, the Netherlands, July 2015. Euro Surveill. 2015;20(30):2-4.

193. Gozalbo M, Guillen M, Taroncher-Ferrer S, Cifre S, Carmena D, Soriano JM, et al. Assessment of the nutritional status, diet and intestinal parasites in hosted Saharawi children. Children (Basel). 2020;7(12):18.

194. Khachfe HH, Zayyoun FJ, Sharif-Askari E, Ramadan W, Hallal N, Khachfe HM. Effect of leishmaniasis on the performance of elementary school students: A case study among syrian refugees in some bekaa (lebanon) area schools. J Epidemiol Glob Health. 2019;9(4):266-73.

195. Özbilgin A, Gencoglan G, Tunali V, Çavuş İ, Yıldırım A, Gündüz C, et al. Refugees at the crossroads of continents: A molecular approach for cutaneous leishmaniasis among refugees in Turkey. Acta Parasitologica. 2020;65(1):136-43.

196. Yauba SM, Rabasa AI, Farouk AG, Elechi HA, Ummate I, Ibrahim BA, et al. Urinary schistosomiasis in Boko Haram-related internally displaced Nigerian children. Saudi J Kidney Dis Transpl. 2018;29(6):1395-402.

197. Yentur Doni N, Gurses G, Dikme R, Aksoy M, Yildiz Zeyrek F, Simsek Z, et al. Cutaneous leishmaniasis due to three Leishmania species among Syrian refugees in Sanliurfa, Southeastern Turkey. Acta Parasitol. 2020;65(4):936-48.

198. Zambrano LD, Samson O, Phares C, Jentes E, Weinberg M, Goers M, et al. Unresolved Splenomegaly in recently resettled Congolese refugees - Multiple States, 2015-2018. MMWR. 2018;67(49):1358-62.

199. Ahmed A, Eldigail M, Elduma A, Breima T, Dietrich I, Ali Y, et al. First report of epidemic dengue fever and malaria co-infections among internally displaced persons in humanitarian camps of North Darfur, Sudan. International Journal of Infectious Diseases. 2021;108:513-6. doi:10.1016/j.ijid.2021.05.052.

200. Altinel Y, Tas B. How to predict the diagnosis of cutaneous leishmaniasis in a non-endemic region. Indian Journal of Dermatology. 2022;67(3):232-8. doi:10.4103/ijd.IJD_452_20.

201. Armitage AJ, Cohen J, Heys M, Hardelid P, Ward A, Eisen S. Description and evaluation of a pathway for unaccompanied asylum-seeking children. Archives of disease in childhood. 2022;107(5):456-60. doi:10.1136/archdischild-2021-322319.

202. Aro T, Kantele A. Hospital admissions of refugees, asylum seekers and undocumented migrants: Ten-year retrospective study. Travel Medicine and Infectious Disease. 2021;44. doi:10.1016/j.tmaid.2021.102186.

203. Balakrishnan VS. Impact of COVID-19 on migrants and refugees. The Lancet Infectious diseases. 2021;21(8):1076-7. doi:10.1016/S1473-3099(21)00410-2.

204. Bergevin A, Husain M, Cruz M, Blanc CL, Dieme A, Girardin ML, et al. Medical check-up of newly arrived unaccompanied minors: A dedicated pediatric consultation service in a hospital. Archives de Pediatrie. 2021;28(8):689-95. doi:10.1016/j.arcped.2021.09.012.

205. Carreras-Abad C, Oliveira-Souto I, Pou-Ciruelo D, Pujol-Morro JM, Soler-Palacín P, Soriano-Arandes A, et al. Health and vaccination status of unaccompanied minors after arrival in a European border country: A cross-sectional study (2017-2020). Pediatric Infectious Disease Journal. 2022;41(11):872-7. doi:10.1097/INF.0000000000003670.

206. Cortier M, de La Porte C, Papot E, Goudjo A, Guenneau L, Riou F, et al. Health status and healthcare trajectory of vulnerable asylum seekers hosted in a French Reception Center. Travel Medicine and Infectious Disease. 2022;46. doi:10.1016/j.tmaid.2021.102180.

207. da Costa e Silva GR, Martins TLS, de Almeida Silva C, Caetano KAA, dos Santos Carneiro MA, Silva BVDE, et al. Hepatitis A and E among immigrants and refugees in Central Brazil. Revista de Saude Publica. 2022;56. doi:10.11606/S1518-8787.2022056003839.

208. Debus D, Genç S, Kurz P, Holzer M, Bauer K, Heimke-Brinck R, et al. Case Report: Local treatment of a Leishmania tropica infection in a Syrian Child with a novel filmogenic preparation of pharmaceutical sodium chlorite. Am J Trop Med Hyg. 2022;106(3):857-60. doi:10.4269/ajtmh.21-0962.

209. Evbuomwan IO, Edosomwan EU, Idubor V, Bazuaye C, Abhulimhen-Iyoha BI, Adeyemi OS, et al. Survey of intestinal parasitism among schoolchildren in internally displaced persons camp, Benin City, Nigeria. Scientific African. 2022;17. doi:10.1016/j.sciaf.2022.e01373.

210. Gumisiriza N, Kugler M, Brusselaers N, Mubiru F, Anguzu R, Ningwa A, et al. Risk factors for nodding syndrome and other forms of epilepsy in northern uganda: A case-control study. Pathogens. 2021;10(11). doi:10.3390/pathogens10111451.

211. Hammoud S, Onchonga D, Amer F, Kocsis B. The burden of communicable diseases in Lebanon: Trends in the past decade. Disaster Medicine and Public Health Preparedness. 2022;16(5):1725-7. doi:10.1017/dmp.2021.200.

212. Hanapi IRM, Sahimin N, Maackara MJB, Annisa AS, Mutalib R, Lewis JW, et al. Prevalence of anti-Leptospira antibodies and associated risk factors in the Malaysian refugee communities. BMC Infect Dis. 2021;21(1). doi:10.1186/s12879-021-06830-0.

213. Kumar GS, Pezzi C, Payton C, Mamo B, Urban K, Scott K, et al. Health of asylees compared to refugees in the United States using domestic medical examination data, 2014-2016: A cross-sectional analysis. Clinical Infectious Diseases. 2021;73(8):1492-9. doi:10.1093/cid/ciab502.

214. Mazhar MKA, Finger F, Evers ES, Kuehne A, Ivey M, Yesurajan F, et al. An outbreak of acute jaundice syndrome (AJS) among the Rohingya refugees in Cox’s Bazar, Bangladesh: Findings from enhanced epidemiological surveillance. PLoS One. 2021;16(4). doi:10.1371/journal.pone.0250505.

215. Müller F, Chandra S, Bogoch II, Rashid M, Redditt V. Intestinal parasites in stool testing among refugees at a primary care clinic in Toronto, Canada. BMC Infect Dis. 2022;22(1). doi:10.1186/s12879-022-07226-4.

216. Tamarozzi F, Ursini T, Ronzoni N, Monteiro GB, Gobbi FG, Angheben A, et al. Prospective cohort study using ultrasonography of Schistosoma haematobium–infected migrants. Journal of Travel Medicine. 2021;28(6). doi:10.1093/jtm/taab122.

217. Webster JL, Stauffer WM, Mitchell T, Lee D, O’Connell EM, Weinberg M, et al. Cross-sectional assessment of the association of eosinophilia with intestinal parasitic infection in U.S.-bound refugees in Thailand: Prevalent, age dependent, but of limited clinical utility. Am J Trop Med Hyg. 2022;106(5):1552-9. doi:10.4269/ajtmh.21-0853.

218. Zöllkau J, Ankert J, Pletz MW, Mishra S, Seliger G, Lobmaier SM, et al. Hepatitis E, schistosomiasis and echinococcosis–prevalence in a cohort of pregnant migrants in Germany and their influence on fetal growth restriction. Pathogens. 2022;11(1). doi:10.3390/pathogens11010058.

219. Beltrame A, Buonfrate D, Gobbi F, Angheben A, Marchese V, Monteiro GB, et al. The hidden epidemic of schistosomiasis in recent African immigrants and asylum seekers to Italy. European Journal of Epidemiology. 2017;32(8):733-5.

220. Enkelmann J, Stark K, Faber M. Epidemiological trends of notified human brucellosis in Germany, 2006–2018. Int J Infect Dis. 2020;93:353-8.

221. Saikal SL, Ge L, Mir A, Pace J, Abdulla H, Leong KF, et al. Skin disease profile of Syrian refugees in Jordan: a field-mission assessment. J Eur Acad Dermatol Venereol. 2020;34(2):419-25. doi: 10.1111/jdv.15909.

222. Sharara SL, Kanj SS. War and infectious diseases: challenges of the Syrian civil war. PLoS Pathog. 2014;10(10):e1004438. doi: 10.1371/journal.ppat.1004438.

223. Ahmed JA, Moturi E, Spiegel P, Schilperoord M, Burton W, Kassim NH, et al. Hepatitis E outbreak, Dadaab refugee camp, Kenya, 2012. Emerg Infect Dis. 2013;19(6):1010-2.

224. Ralli M, Cedola C, Urbano S, Latini O, Shkodina N, Morrone A, et al. Assessment of SARS-CoV-2 infection through rapid serology testing in the homeless population in the City of Rome, Italy. Preliminary results. J Public Health Res. 2020;9(4):556-9.

225. Ahmed A, Ali Y, Siddig EE, Hamed J, Mohamed NS, Khairy A, et al. Hepatitis E virus outbreak among Tigray war refugees from Ethiopia, Sudan. Emerg Infect Dis. 2022;28(8):1722-4. doi:10.3201/eid2808.220397.

226. Aksin S, Cim N, Andan C, Tunc S, Goklu MR. Comparison of obstetric and infectious results among Syrian pregnant women. Annals of Clinical and Analytical Medicine. 2021;12(5):501-5. doi:10.4328/acam.20411.

227. Al-Hatamleh MAI, Hatmal MM, Mustafa SHF, Alzu'bi M, AlSou'b AF, Abughanam SNS, et al. Experiences and perceptions of COVID-19 infection and vaccination among Palestinian refugees in Jerash camp and Jordanian citizens: a comparative cross-sectional study by face-to-face interviews. Infectious diseases of poverty. 2022;11(1):123. doi:10.1186/s40249-022-01047-y.

228. Alawa J, Al-Ali S, Walz L, Wiles E, Harle N, Awale MA, et al. Knowledge and perceptions of COVID-19, prevalence of pre-existing conditions and access to essential resources in Somali IDP camps: a cross-sectional study. BMJ Open. 2021;11(6). doi:10.1136/bmjopen-2020-044411.

229. Altare C, Kostandova N, Okeeffe J, Hayek H, Fawad M, Musa Khalifa A, et al. COVID-19 epidemiology and changes in health service utilization in Azraq and Zaatari refugee camps in Jordan: A retrospective cohort study. PLoS Medicine. 2022;19(5). doi:10.1371/journal.pmed.1003993.

230. Altare C, Kostandova N, Okeeffe J, Omwony E, Nyakoojo R, Kasozi J, et al. COVID-19 epidemiology and changes in health service utilization in Uganda’s refugee settlements during the first year of the pandemic. BMC Public Health. 2022;22(1):1927. doi: 10.1186/s12889-022-14305-3.

231. Binga WE, Houmsou RS, Garba LC, Amuta EU, Suntaya KL. Use of rivers' water, inadequate hygiene, and sanitation as exposure of internally displaced persons (IDPs) to urogenital schistosomiasis and soil-transmitted helminthiasis in Jalingo Local Government Area (LGA), Taraba State, Nigeria. Journal of Water Sanitation and Hygiene for Development. 2022. doi:10.2166/washdev.2022.089.

232. Bojorquez-Chapela I, Strathdee SA, Garfein RS, Benson CA, Chaillon A, Ignacio C, et al. The impact of the COVID-19 pandemic among migrants in shelters in Tijuana, Baja California, Mexico. BMJ Global Health. 2022;7(3). doi: doi:10.1136/bmjgh-2021-007202.

233. da Silva HP, Abreu IN, Lima CNC, de Lima ACR, Barbosa AD, de Oliveira LR, et al. Migration in times of pandemic: SARS-CoV-2 infection among the Warao indigenous refugees in Belem, Para, Amazonia, Brazil. BMC Public Health. 2021;21(1). doi:10.1186/s12889-021-11696-7.

234. Fabris S, d'Ettorre G, Spagnolello O, Russo A, Lopalco M, D'Agostino F, et al. SARS-CoV-2 among migrants recently arrived in Europe from low- and middle-income countries: Containment strategies and special features of management in reception centers. Frontiers in Public Health. 2021;9. doi:10.3389/fpubh.2021.735601.

235. Geleto GE, Kassa T, Erko B. Epidemiology of soil-transmitted helminthiasis and associated malnutrition among under-fives in conflict affected areas in southern Ethiopia. Tropical Medicine and Health. 2022;50(1). doi:10.1186/s41182-022-00436-1.

236. Gignoux E, Athanassiadis F, Yarrow AG, Jimale A, Mubuto N, Déglise C, et al. Seroprevalence of SARS-CoV-2 antibodies and retrospective mortality in a refugee camp, Dagahaley, Kenya. PLoS One. 2021;16(12). doi:10.1371/journal.pone.0260989.

237. Johnson-Agbakwu CE, Eakin CM, Bailey CV, Sood S, Ali N, Doehrman P, et al. Severe acute respiratory syndrome coronavirus 2: a canary in the coal mine for public safety net hospitals. AJOG Global Reports. 2021;1(2). doi:10.1016/j.xagr.2021.100009.

238. Kalani N, Hatami N, Haghbeen M, Yaqoob U, Raeyat Doost E. Covid-19 health care for afghan refugees as a minor ethnicity in iran; clinical differences and racial equality in health. Acta Medica Iranica. 2021;59(8):466-71.

239. Kheirallah KA, Ababneh BF, Bendak H, Alsuwaidi AR, Elbarazi I. Exploring the mental, social, and lifestyle effects of a positive COVID-19 infection on syrian refugees in Jordan: A qualitative study. International Journal of Environmental Research and Public Health. 2022;19(19). doi:10.3390/ijerph191912588.

240. Mellou K, Gkolfinopoulou K, Andreopoulou A, Tsekou A, Papadima K, Stamoulis K, et al. A COVID-19 outbreak among migrants in a hosting facility in Greece, April 2020. Journal of Infection Prevention. 2022;23(5):235-8. doi:10.1177/17571774221092568.

241. Nyakarahuka L, Whitmer S, Kyondo J, Mulei S, Cossaboom CM, Telford CT, et al. Crimean-Congo hemorrhagic fever outbreak in refugee settlement during COVID-19 pandemic, Uganda, April 2021. Emerg Infect Dis. 2022;28(11):2326-9. doi:10.3201/eid2811.220365.

242. Palacios CF, Tucker EW, Travassos MA. Coronavirus disease 2019 burden among unaccompanied minors in US custody. Clinical Infectious Diseases. 2022. doi:10.1093/cid/ciac636.

243. Pham PN, Keegan K, Johnston LG, Rodas J, Restrepo MA, Wei C, et al. Assessing the impact of the COVID-19 pandemic among Venezuelan refugees and migrants in Colombia using respondent-driven sampling (RDS). BMJ Open. 2022;12(10). doi:10.1136/bmjopen-2021-054820.

244. Sharov KS. SARS-CoV-2 spread in different biosocial strata in Russia in 2020: Groups of risk and victimised groups. Journal of Global Health. 2021;11. doi:10.7189/jogh.11.03066.

245. Sisti LG, Di Napoli A, Petrelli A, Rossi A, Diodati A, Menghini M, et al. Covid-19 impact in the italian reception system for migrants during the nationwide lockdown: A national observational study. International Journal of Environmental Research and Public Health. 2021;18(23). doi:10.3390/ijerph182312380.

246. Tambuzzi S, Cummaudo M, Maggioni L, Tritella S, Lucchesi B, Montedoro P, et al. A pilot COVID-19 surveillance program at the Zendrini center in Milan (Italy) for unaccompanied foreign minors. Children (Basel). 2022;9(10). doi:10.3390/children9101485.

247. Tolunay O, Çelik Ü, Arslan I, Tutun B, Özkaya M. Evaluation of clinical findings and treatment results of Coronavirus disease 2019 (COVID-19) in pediatric cancer patients: A single center experience. Frontiers in Pediatrics. 2022;10. doi:10.3389/fped.2022.848379.

248. Vallejo-Janeta AP, Morales-Jadan D, Freire-Paspuel B, Lozada T, Cherrez-Bohorquez C, Garcia-Bereguiain MA, et al. COVID-19 outbreaks at shelters for women who are victims of gender-based violence from Ecuador. International Journal of Infectious Diseases. 2021;108:531-6. doi:10.1016/j.ijid.2021.06.012.

249. Wamala JF, Loro F, Deng SJ, Berta KK, Guyo AG, Mpairwe A, et al. Epidemiological characterization of COVID-19 in displaced populations of South Sudan. Pan African Medical Journal. 2022;41(2). doi:10.11604/pamj.supp.2022.42.1.33767.

250. Yan XY, Xiao W, Zhou SP, Wang XC, Wang ZK, Zhao MC, et al. A four-generation family transmission chain of COVID-19 along the China-Myanmar border in October to November 2021. Frontiers in Public Health. 2022;10. doi:10.3389/fpubh.2022.1004817.

251. Zhang M, Gurung A, Anglewicz P, Baniya K, Yun K. Discrimination and stress among Asian refugee populations during the COVID-19 pandemic: Evidence from Bhutanese and Burmese refugees in the USA. Journal of Racial and Ethnic Health Disparities. 2022;9(2):589-97. doi:10.1007/s40615-021-00992-y.

252. Khan S, Akbar SMF, Kimitsuki K, Saito N, Yahiro T, Al Mahtab M, et al. Recent downhill course of COVID-19 at Rohingya refugee camps in Bangladesh: Urgent action solicited. J Glob Health. 2021;11:03097. doi: 10.7189/jogh.11.03097.
