# Supplementary material for: Status of zoonotic disease research in refugees, asylum seekers and internally displaced people, globally: A scoping review of forty clinically important zoonotic pathogens
Source: PLoS Negl Trop Dis. 2024 May 20;18(5):e0012164. doi: 10.1371/journal.pntd.0012164 (PMC11142688; doi:10.1371/journal.pntd.0012164)
Supplement: S8 Table — (DOCX) [file pntd.0012164.s010.docx]

**S8 Table:** **Publications included in the scoping review reporting on zoonotic pathogens in displaced people from Africa, Asia and Latin America**

| Region | References |
| --- | --- |
| Africa | [1-109] |
| Asia | [22, 25, 92, 109-241] |
| Latin America | [109, 242-250] |

**References**

1. Enterically transmitted non-A, non-B hepatitis--East Africa. MMWR. 1987;36(16):241-4.

2. Hepatitis E, Chad. Weekly epidemiological record / Health Section of the Secretariat of the League of Nations. 2004;79(35):313.

3. Abd Rahman MM, Bryant P, Guppy D, Buttery J, Burgner D. Intermittent fever, splenomegaly and eosinophilia in a recently resettled African refugee. J Paediatr Child Health. 2012;48(10):939-41.

4. Ackermann N, Marosevic D, Hörmansdorfer S, Eberle U, Rieder G, Treis B, et al. Screening for infectious diseases among newly arrived asylum seekers, Bavaria, Germany, 2015. Eurosurveillance. 2018;23(10).

5. Ahmed A, Ali Y, Siddig EE, Hamed J, Mohamed NS, Khairy A, et al. Hepatitis E Virus outbreak among Tigray war refugees from Ethiopia, Sudan. Emerg Infect Dis. 2022;28(8):1722-4. doi:10.3201/eid2808.220397.

6. Aldulaimi S, Mendez A. Chronic abdominal pain and hepatosplenomegaly in a refugee patient. Travel Med Infect Dis. 2021;41:102009.

7. Angheben A, Mariconti M, Degani M, Gobbo M, Palvarini L, Gobbi F, et al. Is there echinococcosis in West Africa? A refugee from Niger with a liver cyst. Parasit Vectors. 2017;10(1).

8. Antinori S, Mediannikov O, Corbellino M, Gr, e R, Parravicini C, et al. Louse-Borne Relapsing Fever (Borrelia recurrentis) in a Somali Refugee Arriving in Italy: A Re-emerging Infection in Europe? PLoS Negl Trop Dis. 2016;10(5).

9. Antinori S, Mediannikov O, Corbellino M, Raoult D. Louse-borne relapsing fever among East African refugees in Europe. Travel Med Infect Dis. 2016;14(2):110-4.

10. Antinori S, Tonello C, Edouard S, Parravicini C, Gastaldi D, Gr, et al. Diagnosis of louse-borne relapsing fever despite negative microscopy in two asylum seekers from Eastern Africa. Am J Trop Med Hyg. 2017;97(6):1669-72.

11. Azman AS, Bouhenia M, Iyer AS, Rumunu J, Laku RL, Wamala JF, et al. High hepatitis e seroprevalence among displaced persons in South Sudan. Am J Trop Med Hyg. 2017;96(6):1296-301.

12. Beltrame A, Guerriero M, Angheben A, Gobbi F, Requena-Mendez A, Zammarchi L, et al. Accuracy of parasitological and immunological tests for the screening of human schistosomiasis in immigrants and refugees from African countries: An approach with Latent Class Analysis. PLoS Negl Trop Dis. 2017;11(6).

13. Benson J. Asymptomatic schistosomiasis in a young Sudanese refugee. Australian Family Physician. 2007;36(4):249-51.

14. Berger SA, Schwartz T, Michaeli D. Infectious disease among Ethiopian immigrants in Israel. Arch Intern Med. 1989;149(1):117-9.

15. Bliss J, Bouhenia M, Hale P, Couturier BA, Iyer AS, Rumunu J, et al. High prevalence of shigella or enteroinvasive Escherichia coli carriage among residents of an internally displaced persons camp in South Sudan. Am J Trop Med Hyg. 2018;98(2):595-7.

16. Bloch-Infanger C, Bättig V, Kremo J, Widmer AF, Egli A, Bingisser R, et al. Increasing prevalence of infectious diseases in asylum seekers at a tertiary care hospital in Switzerland. PLoS One. 2017;12(6):e0179537.

17. Boccia D, Guthmann JP, Klovstad H, Hamid N, Tatay M, Ciglenecki I, et al. High mortality associated with an outbreak of hepatitis E among displaced persons in Darfur, Sudan. Clinical Infectious Diseases. 2006;42(12):1679-84.

18. Borch M, Kiernan M, Rust K, Baron B, Simmons B, Hattala P, et al. Schistosomiasis: a case study. Urol Nurs. 2009;29(1):26-9.

19. Brodine SK, Thomas A, Huang R, Harbertson J, Mehta S, Leake J, et al. Community based parasitic screening and treatment of sudanese refugees: Application and assessment of centers for disease control guidelines. Am J Trop Med Hyg. 2009;80(3):425-30.

20. Brown V, Larouze B, Desve G, Rousset JJ, Thibon M, Fourrier A, et al. Clinical presentation of louse-born relapsing fever among Ethiopian refugees in northern Somalia. Ann Trop Med Parasitol. 1988;82(5):499-502.

21. Browne LB, Menkir Z, Kahi V, Maina G, Asnakew S, Tubman M, et al. Hepatitis E outbreak among refugees from South Sudan - Gambella, Ethiopia, April 2014-January 2015. MMWR. 2015;64(19):537-.

22. Buonfrate D, Gobbi F, Marchese V, Postiglione C, Monteiro GB, Giorli G, et al. Extended screening for infectious diseases among newly-arrived asylum seekers from Africa and Asia, Verona province, Italy, April 2014 to June 2015. Eurosurveillance. 2019;23(16):7-14.

23. Ceccarelli G, d'Ettorre G, Riccardo F, Ceccarelli C, Chiaretti M, Picciarella A, et al. Urinary schistosomiasis in asylum seekers in Italy: an emergency currently undervalued. J Immigr Minor Health. 2013;15(4):846-50.

24. Chalupa P, Vanista J, Burget I, Stary J, Sukova M, Nohynkova M. The review of imported visceral leishmaniosis in the Czech Republic. Bratislavské lekárske listy. 2001;102(2):84-91.

25. Chang AH, Perry S, Du JNT, Agunbiade A, Polesky A, Parsonnet J. Decreasing intestinal parasites in recent northern California refugees. Am J Trop Med Hyg. 2013;88(1):191-7.

26. Cherian P, Junckerstorff RK, Rosen D, Kumarasinghe P, Morling A, Tuch P, et al. Late-stage human African trypanosomiasis in a Sudanese refugee. Medical Journal of Australia. 2010;192(7):417-9.

27. Chernet A, Kling K, Sydow V, Kuenzli E, Hatz C, Utzinger J, et al. Accuracy of diagnostic tests for Schistosoma mansoni infection in asymptomatic Eritrean refugees: Serology and point-of-care circulating cathodic antigen against stool microscopy. Clinical Infectious Diseases. 2017;65(4):568-74.

28. Costescu Strachinaru DI, Cambier J, et-Yattara H, Konopnicki D. Relapsing fever in asylum seekers from Somalia arriving in Belgium in August 2015. Acta Clin Belg. 2016;71(5):353-5.

29. Cutuli SL, De Pascale G, Spanu T, Dell'Anna AM, Bocci MG, Pallavicini F, et al. Lice, rodents, and many hopes: a rare disease in a young refugee. Crit Care. 2017;21:3.

30. Darcis G, Hayette MP, Bontems S, Sauvage AS, Meuris C, Van Esbroeck M, et al. Louse-borne relapsing fever in a refugee from Somalia arriving in Belgium. J Travel Med. 2016;23(3):3.

31. Daveson J, Macdonald G. A case of periportal fibrosis in a Sudanese refugee. Medical Journal of Australia. 2008;188(11):677-8.

32. Dorkenoo MA, Tchankoni MK, Yehadji D, Yakpa K, Tchalim M, Sossou E, et al. Monitoring migrant groups as a post-validation surveillance approach to contain the potential reemergence of lymphatic filariasis in Togo. Parasites & Vectors. 2021;14(1).

33. Edosomwan EU, Evbuomwan IO, Agbalalah C, Dahunsi SO, Abhulimhen-Iyoha BI. Malaria coinfection with Neglected Tropical Diseases (NTDs) in children at Internally Displaced Persons (IDP) camp in Benin City, Nigeria. Heliyon. 2020;6(8).

34. Ehlkes L, George M, Knautz D, Burckhardt F, Jahn K, Vogt M, et al. Negligible import of enteric pathogens by newly-arrived asylum seekers and no impact on incidence of notified Salmonella and Shigella infections and outbreaks in Rhineland-Palatinate, Germany, January 2015 to May 2016. Euro Surveill. 2018;23(20):7-14.

35. Elias AF, Peterson SN, Huntington MK. Typhoid fever in a young immigrant child: a case report and review of the literature. South Dakota medicine : the journal of the South Dakota State Medical Association. 2008;61(7):255-8.

36. Enabulele EE, Platt RN, Adeyemi E, Agbosua E, Aisien MSO, Ajakaye OG, et al. Urogenital schistosomiasis in Nigeria post receipt of the largest single praziquantel donation in Africa. Acta Tropica. 2021:105916.

37. Franco-Paredes C, Dismukes R, Nicolls D, Hidron A, Workowski K, Rodriguez-Morales A, et al. Persistent and untreated tropical infectious diseases among Sudanese refugees in the United States. Am J Trop Med Hyg. 2007;77(4):633-5.

38. Geltman PL, Cochran J, Hedgecock C. Intestinal parasites among African refugees resettled in Massachusetts and the impact of an overseas pre-departure treatment program. Am J Trop Med Hyg. 2003;69(6):657-62.

39. Goldenberger D, Claas GJ, Bloch-Infanger C, Breidthardt T, Suter B, Martinez M, et al. Louse-borne relapsing fever (Borrelia recurrentis) in an Eritrean refugee arriving in Switzerland, August 2015. Euro Surveill. 2015;20(32):2-5.

40. Gray GC, Rodier GR, Matras-Maslin VC, Honein MA, Ismail EA, Botros BA, et al. Serologic evidence of respiratory and rickettsial infections among Somali refugees. Am J Trop Med Hyg. 1995;52(4):349-53. doi: 10.4269/ajtmh.1995.52.349.

41. Grecchi C, Zanotti P, Pontarelli A, Chiari E, Tomasoni LR, Gulletta M, et al. Louse-borne relapsing fever in a refugee from Mali. Infection. 2017;45(3):373-6.

42. Hoch M, Wieser A, Löscher T, Margos G, Pürner F, Zühl J, et al. Louse-borne relapsing fever (Borrelia recurrentis) diagnosed in 15 refugees from northeast Africa: Epidemiology and preventive control measures, Bavaria, Germany, July to October 2015. Euro Surveill. 2015;20(42).

43. Hytönen J, Khawaja T, Grönroos JO, Jalava A, Meri S, Oksi J. Louse-borne relapsing fever in Finland in two asylum seekers from Somalia. APMIS. 2017;125(1):59-62.

44. Jama S, Manivel JC, Abd Alla MD, Stauffer WM. Appendectomy to remember. J Travel Med. 2009;16(4):295-6.

45. Jenkins-Holick DS, Kaul TL. Schistosomiasis. Urol Nurs. 2013;33(4):163-70.

46. Keller C, Zumblick M, Streubel K, Eickmann M, Müller D, Kerwat M, et al. Hemorrhagic diathesis in Borrelia recurrentis infection imported to Germany. Emerg Infect Dis. 2016;22(5):917-9.

47. Lagare A, Ibrahim A, Ousmane S, Issaka B, Zaneidou M, Kadadé G, et al. Outbreak of Hepatitis E virus infection in displaced persons camps in Diffa region, Niger, 2017. Am J Trop Med Hyg. 2018;99(4):1055-7.

48. Lucchini A, Lipani F, Costa C, Scarvaglieri M, Balbiano R, Carosella S, et al. Louseborne relapsing fever among East African refugees, Italy, 2015. Emerg Infect Dis. 2016;22(2):298-301.

49. Lucey JM, McCarthy J, Burgner DP. Encysted seizures: status epilepticus in a recently resettled refugee child. Medical Journal of Australia. 2010;192(4):237.

50. Marlet MVL, Wuillaume F, Jacquet D, Quispe KW, Dujardin JC, Boelaert M. A neglected disease of humans: A new focus of visceral leishmaniasis in Bakool, Somalia. Trans R Soc Trop Med Hyg. 2003;97(6):667-71.

51. Marnell F, Guillet A, Holl, C. A survey of the intestinal helminths of refugees in Juba, Sudan. Annals of Tropical Medicine and Parasitology. 1992;86(4):387-93.

52. Mekonnen GK, Mengistie B, Sahilu G, Kloos H, Mulat W. Etiologies of diarrhea and drug susceptibility patterns of bacterial isolates among under-five year children in refugee camps in Gambella Region, Ethiopia: a case control study. BMC Infect Dis. 2019;19(1).

53. Mérens A, Guérin PJ, Guthmann JP, Nic, E. Outbreak of hepatitis E virus infection in Darfur, Sudan: Effectiveness of real-time reverse transcription-PCR analysis of dried blood spots. J Clin Microbiol. 2009;47(6):1931-3.

54. Miller JM, Boyd HA, Ostrowski SR, Cookson ST, Parise ME, Gonzaga PS, et al. Malaria, intestinal parasites, and schistosomiasis among Barawan Somali refugees resettling to the United States: A strategy to reduce morbidity and decrease the risk of imported infections. Am J Trop Med Hyg. 2000;62(1):115-21.

55. Montour J, Lee D, Snider C, Jentes ES, Stauffer W. Absence of Loa loa microfilaremia among newly arrived congolese refugees in Texas. Am J Trop Med Hyg. 2017;97(6):1833-5.

56. Neal PM. Schistosomiasis--an unusual cause of ureteral obstruction: a case history and perspective. Clin Med Res. 2004;2(4):216-27.

57. Neumayr A, Chernet A, Sydow V, Kling K, Kuenzli E, Marti H, et al. Performance of the point-of-care circulating cathodic antigen (POC-CCA) urine cassette test for follow-up after treatment of S. mansoni infection in Eritrean refugees. Travel Medicine and Infectious Disease. 2019;28:59-63.

58. Nicand E, Armstrong GL, Enouf V, Guthmann JP, Guerin JP, Caron M, et al. Genetic heterogeneity of hepatitis E virus in Darfur, Sudan, and neighboring Chad. Journal of Medical Virology. 2005;77(4):519-21.

59. Nyamusore J, Nahimana MR, Ngoc CT, Olu O, Isiaka A, Ndahindwa V, et al. Risk factors for transmission of Salmonella Typhi in Mahama refugee camp, Rwanda: a matched case-control study. Pan African Medical Journal. 2018;29:13.

60. Ofoezie IE, Asaulu SO, Christensen NØ, Madsen H. Patterns of infection with Schistosoma haematobium in lakeside resettlement communities at the Oyan Reservoir in Ogun State, south-western Nigeria. Ann Trop Med Parasitol. 1997;91(2):187-97.

61. Osthoff M, Schibli A, Fadini D, Lardelli P, Goldenberger D. Louse-borne relapsing fever - report of four cases in Switzerland, June-December 2015. BMC Infect Dis. 2016;16(1).

62. Paran Y, Ben-Ami R, Orlev B, Halutz O, Elalouf O, Wasserman A, et al. Chronic schistosomiasis in African immigrants in Israel: Lessons for the non-endemic setting. Medicine (Baltimore). 2019;98(52).

63. Parenti DM, Lucas D, Lee A, Hollenkamp RH. Health status of Ethiopian refugees in the United States. Am J Public Health. 1987;77(12):1542-3.

64. Perea WA, Ancelle T, Moren A, Nagelkerke M, Sondorp E. Visceral leishmaniasis in southern Sudan. Trans R Soc Trop Med Hyg. 1991;85(1):48-53.

65. Poddighe D, Castelli L, Pulcrano G, Grosini A, Balzaretti M, Spadaro S, et al. Urinary Schistosomiasis in an Adolescent Refugee from Africa: An Uncommon Cause of Hematuria and an Emerging Infectious Disease in Europe. J Immigr Minor Health. 2016;18(5):1237-40.

66. Posey DL, Blackburn BG, Weinberg M, Flagg EW, Ortega L, Wilson M, et al. High prevalence and presumptive treatment of schistosomiasis and strongyloidiasis among African refugees. Clinical Infectious Diseases. 2007;45(10):1310-5.

67. Raoult D, Ndihokubwayo JB, Tissot-Dupont H, Roux V, Faugere B, Abegbinni R, et al. Outbreak of epidemic typhus associated with trench fever in Burundi. The Lancet. 1998;352(9125):353-8.

68. Redditt VJ, Janakiram P, Graziano D, Rashid M. Health status of newly arrived refugees in Toronto, Ont: Part 1: infectious diseases. Can Fam Physician. 2015;61(7):e303-e9.

69. Richter J, Bode JG, Blondin D, Kircheis G, Kubitz R, Holtfreter MC, et al. Severe liver fibrosis caused by Schistosoma mansoni: Management and treatment with a transjugular intrahepatic portosystemic shunt. The Lancet Infectious Diseases. 2015;15(6):731-7.

70. Sahlas DJ, Dick MacLean J, Janevski J, Detsky AS. Clinical problem-solving. Out of Africa. N Engl J Med. 2002;347(10):749-53.

71. Schmid M, Dodt C. Multiple organ failure in a young asylum-seeker. Dtsch Arztebl Int. 2017;114(37):625.

72. Schweickert B, Bollmann R, Loui A, Kaufmann O, Kluttig L, Feiterna-Sperling C, et al. Fatal disseminated toxoplasmosis with congenital transmission in an African migrant. AIDS (London, England). 2008;22(12):1523-5.

73. Shorter D, Makone I, Elliott EJ. Fever and urticaria in an African refugee. Journal of Paediatrics and Child Health. 2006;42(11):731-3.

74. Sulaiman AA, Elmadhoun WM, Noor SK, Bushara SO, Almobarak AO, Awadalla H, et al. An outbreak of cutaneous leishmaniasis among a displaced population in North Sudan: Review of cases. J Family Med Prim Care. 2019;8(2):556-63.

75. Ahmed A, Eldigail M, Elduma A, Breima T, Dietrich I, Ali Y, et al. First report of epidemic dengue fever and malaria co-infections among internally displaced persons in humanitarian camps of North Darfur, Sudan. International Journal of Infectious Diseases. 2021;108:513-6. doi:10.1016/j.ijid.2021.05.052.

76. Alawa J, Al-Ali S, Walz L, Wiles E, Harle N, Awale MA, et al. Knowledge and perceptions of COVID-19, prevalence of pre-existing conditions and access to essential resources in Somali IDP camps: a cross-sectional study. BMJ Open. 2021;11(6). doi:10.1136/bmjopen-2020-044411.

77. Altare C, Kostandova N, Okeeffe J, Omwony E, Nyakoojo R, Kasozi J, et al. COVID-19 epidemiology and changes in health service utilization in Uganda’s refugee settlements during the first year of the pandemic. BMC Public Health. 2022;22(1):1927. doi: 10.1186/s12889-022-14305-3.

78. Binga WE, Houmsou RS, Garba LC, Amuta EU, Suntaya KL. Use of rivers' water, inadequate hygiene, and sanitation as exposure of internally displaced persons (IDPs) to urogenital schistosomiasis and soil-transmitted helminthiasis in Jalingo Local Government Area (LGA), Taraba State, Nigeria. Journal of Water Sanitation and Hygiene for Development. 2022. doi:10.2166/washdev.2022.089.

79. Gozalbo M, Guillen M, Taroncher-Ferrer S, Cifre S, Carmena D, Soriano JM, et al. Assessment of the nutritional status, diet and intestinal parasites in hosted Saharawi children. Children (Basel). 2020;7(12):18.

80. Hershko C, Nesher G, Yinnon AM. Medical problems in Ethiopian refugees airlifted to Israel: Experience in 131 patients admitted to a general hospital. J Trop Med Hyg. 1986;89(3):107-12.

81. Newman RD, Schwartz MA. Hematuria in two school-age refugee brothers from Africa. Pediatr Emerg Care. 1999;15(5):335-7.

82. Summer AP, Stauffer W, Maroushek SR, Nevins TE. Hematuria in children due to schistosomiasis in a nonendemic setting. Clin Pediatr (Phila). 2006;45(2):177-81.

83. Thomson K, Luis Dvorzak J, Lagu J, Laku R, Dineen B, Schilperoord M, et al. Investigation of hepatitis E outbreak among refugees - Upper Nile, South Sudan, 2012-2013. MMWR. 2013;62(29):581-6.

84. Williams B, Boullier M, Cricks Z, Ward A, Naidoo R, Williams A, et al. Screening for infection in unaccompanied asylum-seeking children and young people. Archives of Disease in Childhood. 2020;105(6):530-2.

85. Wilting KR, Stienstra Y, Sinha B, Braks M, Cornish D, Grundmann H. Louse-borne relapsing fever (Borrelia recurrentis) in asylum seekers from Eritrea, the Netherlands, July 2015. Euro Surveill. 2015;20(30):2-4.

86. Yauba SM, Rabasa AI, Farouk AG, Elechi HA, Ummate I, Ibrahim BA, et al. Urinary schistosomiasis in Boko Haram-related internally displaced Nigerian children. Saudi J Kidney Dis Transpl. 2018;29(6):1395-402.

87. Zambrano LD, Samson O, Phares C, Jentes E, Weinberg M, Goers M, et al. Unresolved Splenomegaly in Recently Resettled Congolese Refugees - Multiple States, 2015-2018. MMWR. 2018;67(49):1358-62.

88. Zhao Y, Alex, er B, Bailey JA, Welch L, Greene M, et al. Therapeutic apheresis using a mononuclear cell program to lower the microfilaria burden of a 23-year-old African woman with loiasis. J Clin Apher. 2017;32(3):200-2.

89. Zijlstra EE, Ali MS, El-Hassan AM, El-Toum IA, Satti M, Ghalib Kager HWPA. Direct agglutination test for diagnosis and sero- epidemiological survey of kala-azar in the Sudan. Trans R Soc Trop Med Hyg. 1991;85(4):474-6.

90. Zijlstra EE, Siddig Ali M, El-Hassan AM, El-Toum IA, Satti M, Ghalib HW, et al. Kala-azar in displaced people from southern Sudan: Epidemiological, clinical and therapeutic findings. Trans R Soc Trop Med Hyg. 1991;85(3):365-9.

91. Evbuomwan IO, Edosomwan EU, Idubor V, Bazuaye C, Abhulimhen-Iyoha BI, Adeyemi OS, et al. Survey of intestinal parasitism among schoolchildren in internally displaced persons camp, Benin City, Nigeria. Scientific African. 2022;17. doi: doi:10.1016/j.sciaf.2022.e01373.

92. Fabris S, d'Ettorre G, Spagnolello O, Russo A, Lopalco M, D'Agostino F, et al. SARS-CoV-2 among migrants recently arrived in Europe from low- and middle-income countries: Containment strategies and special features of management in reception centers. Frontiers in Public Health. 2021;9. doi:10.3389/fpubh.2021.735601.

93. Geleto GE, Kassa T, Erko B. Epidemiology of soil-transmitted helminthiasis and associated malnutrition among under-fives in conflict affected areas in southern Ethiopia. Tropical Medicine and Health. 2022;50(1). doi:10.1186/s41182-022-00436-1.

94. Gignoux E, Athanassiadis F, Yarrow AG, Jimale A, Mubuto N, Déglise C, et al. Seroprevalence of SARS-CoV-2 antibodies and retrospective mortality in a refugee camp, Dagahaley, Kenya. PLoS One. 2021;16(12). doi:10.1371/journal.pone.0260989.

95. Gumisiriza N, Kugler M, Brusselaers N, Mubiru F, Anguzu R, Ningwa A, et al. Risk factors for nodding syndrome and other forms of epilepsy in northern uganda: A case-control study. Pathogens. 2021;10(11). doi:10.3390/pathogens10111451.

96. Hoekstra PT, Chernet A, de Dood CJ, Brienen EAT, Corstjens PLAM, Labhardt ND, et al. Sensitive diagnosis and post-treatment follow-up of Schistosoma mansoni infections in asymptomatic Eritrean refugees by circulating anodic antigen detection and polymerase chain reaction. Am J Trop Med Hyg. 2022;106(4):1240-6. doi:10.4269/ajtmh.21-0803.

97. Mellou K, Gkolfinopoulou K, Andreopoulou A, Tsekou A, Papadima K, Stamoulis K, et al. A COVID-19 outbreak among migrants in a hosting facility in Greece, April 2020. Journal of Infection Prevention. 2022;23(5):235-8. doi:10.1177/17571774221092568.

98. Nyakarahuka L, Whitmer S, Kyondo J, Mulei S, Cossaboom CM, Telford CT, et al. Crimean-Congo hemorrhagic fever outbreak in refugee settlement during COVID-19 pandemic, Uganda, April 2021. Emerg Infect Dis. 2022;28(11):2326-9. doi:10.3201/eid2811.220365.

99. Tamarozzi F, Ursini T, Ronzoni N, Monteiro GB, Gobbi FG, Angheben A, et al. Prospective cohort study using ultrasonography of Schistosoma haematobium–infected migrants. Journal of Travel Medicine. 2021;28(6). doi:10.1093/jtm/taab122.

100. (CDC) CfDCaP. Imported dracunculiasis--United States, 1995 and 1997. MMWR. 1998;47(11):209-11.

101. Beltrame A, Buonfrate D, Gobbi F, Angheben A, Marchese V, Monteiro GB, et al. The hidden epidemic of schistosomiasis in recent African immigrants and asylum seekers to Italy. European Journal of Epidemiology. 2017;32(8):733-5.

102. Caruana SR, Kelly HA, Ngeow JYY, Ryan NJ, Bennett CM, Chea L, et al. Undiagnosed and potentially lethal parasite infections among immigrants and refugees in Australia. Journal of Travel Medicine. 2006;13(4):233-9.

103. Guthmann JP, Klovstad H, Boccia D, Hamid N, Pinoges L, Nizou JY, et al. A large outbreak of hepatitis E among a displaced population in Darfur, Sudan, 2004: the role of water treatment methods. Clin Infect Dis. 2006;42(12):1685-91. doi: 10.1086/504321.

104. IsaÃ¤cson M, Frean J, He J, Seriwatana J, Innis BL. An outbreak of hepatitis E in Northern Namibia, 1983. Am J Trop Med Hyg. 2000;62(5):619-25. doi: 10.4269/ajtmh.2000.62.619.

105. Teshale EH, Grytdal SP, Howard C, Barry V, Kamili S, Drobeniuc J, et al. Evidence of person-to-person transmission of hepatitis E virus during a large outbreak in Northern Uganda. Clinical Infectious Diseases. 2010;50(7):1006-10. doi: 10.1086/651077.

106. Wamala JF, Loro F, Deng SJ, Berta KK, Guyo AG, Mpairwe A, et al. Epidemiological characterization of COVID-19 in displaced populations of South Sudan. Pan African Medical Journal. 2022;41(2). doi:10.11604/pamj.supp.2022.42.1.33767.

107. Quandelacy TM, Riefkohl A, Franco-Paredes C. Prevalence of untreated schistosomiasis among Sudanese refugees: “The Lost Boys of Sudan” in the United States. Boletin medico del Hospital Infantil de Mexico. 2010;67:503-6.

108. de Beer P, el Harith A, van Grootheest M, Winkler A. Outbreak of kala-azar in the Sudan. Lancet. 1990;335(8683):224. doi: 10.1016/0140-6736(90)90313-t.

109. Ryan N, Plackett M, Dwyer B. Parasitic infections of refugees. Medical Journal of Australia. 1988;148(10):491-4. doi:10.5694/j.1326-5377.1988.tb99455.x.

110. Haq KUA, Gul NA, Hammad HM, Bibi Y, Bibi A, Mohsan J. Prevalence of Giardia intestinalis and Hymenolepis nana in Afghan refugee population of Mianwali district, Pakistan. Afr Health Sci. 2015;15(2):394-400.

111. Ahmed W, Ahmad M, Rafatullah, Shah F, Sajadullah. Pervasiveness of intestinal protozoan and worm incursion in IDP's (North Waziristan agency, KPK-Pakistan) children of 6-16 years. Journal of the Pakistan Medical Association. 2015;65(9):943-5.

112. Alawieh A, Musharrafieh U, Jaber A, Berry A, Ghosn N, Bizri AR. Revisiting leishmaniasis in the time of war: the Syrian conflict and the Lebanese outbreak. International Journal of Infectious Diseases. 2014;29:115-9.

113. Alhawarat M, Khader Y, Shadfan B, Kaplan N, Iblan I. Trend of Cutaneous Leishmaniasis in Jordan from 2010 to 2016: Retrospective Study. JMIR Public Health and Surveillance. 2020;6(1).

114. Aliskin O, Savas N. Notifiable communicable diseases in Turkey and their notification status: Antakya Sample. Flora the Journal of Infectious Diseases and Clinical Microbiology. 2019;24(1):11-21.

115. Amr ZS, Kanani K, Shadfan B, Hani RB. Cutaneous Leishmaniasis among Syrian Refugees in Jordan: a Retrospective Study. Bull Soc Pathol Exot. 2018;111(5):295-300.

116. Arfaa F. Intestinal parasites among Indochinese refugees and Mexican immigrants resettled in Contra Costa County, California. J Fam Pract. 1981;12(2):223-6.

117. Arthur JD, Bodhidatta L, Echeverria P, Phuphaisan S, Paul S. Diarrheal disease in Cambodian children at a camp in Thailand. Am J Epidemiol. 1992;135(5):541-51.

118. Bizri NA, Alam W, Khoury M, Musharrafieh U, Ghosn N, Berri A, et al. The association between the Syrian crisis and cutaneous Leishmaniasis in Lebanon. Acta Parasitologica. 2021:1-6.

119. Bjazevic J, Golomb D, Silverman MS, Pautler SE, Razvi H. Case report - Primary renal echinococcal infection. Can Urol Assoc J. 2020;14(8):E383-E6.

120. Bradshaw S, Litvinov IV. Dermal leishmaniasis in a 25-year-old Syrian refugee. Canadian Medical Association Journal. 2017;189(45):E1397-E.

121. Brooker S, Mohammed N, Adil K, Agha S, Reithinger R, Rowl, et al. Leishmaniasis in refugee and local Pakistani populations. Emerg Infect Dis. 2004;10(9):1681-4.

122. Brooks AMV, Essex WB, West RH. Cysticercosis of the superior oblique muscle. Aust J Ophthalmol. 1983;11(2):119-22.

123. Brown AE, Meek SR, Maneechai N, Lewis GE. Murine typhus among Khmers living at an evacuation site on the Thai-Kampuchean border. Am J Trop Med Hyg. 1988;38(1):168-71.

124. Chandrasena TGAN, Hapuarachchi HC, Dayanath MYD, Pathmeswaran A, De Silva NR. Intestinal parasites and the growth status of internally displaced children in Sri Lanka. Trop Doct. 2007;37(3):163-5.

125. Chen L, Peek M, Stokich D, Todd R, Anderson M, Murphy FK, et al. Japanese encephalitis in two children-United States, 2010. MMWR. 2011;60(9):276-8.

126. Chironna M, Germinario C, Lopalco PL, Carrozzini F, Barbuti S, Quarto M. Prevalence rates of viral hepatitis infections in refugee Kurds from Iraq and Turkey. Infection. 2003;31(2):70-4.

127. Çizmeci Z, Karakuş M, Karabela ŞN, Erdoğan B, Güleç N. Leishmaniasis in Istanbul; A new epidemiological data about refugee leishmaniasis. Acta Trop. 2019;195:23-7.

128. Çoşkun B, Gülümser Ç, Çoşkun B, Artuk C, Karaşahin KE. Impact of Syrian refugees on congenital TORCH infections screening in Turkey. J Obs and Gynae Research. 2020;46(7):1017-24.

129. Crogan J, Gunasekera H, Wood N, Sheikh M, Isaacs D. Management of old world cutaneous leishmaniasis in refugee children. Pediatr Infect Dis J. 2010;29(4):357-9.

130. D'Alauro F, Lee RV, Pao-In K, Khairallah M. Intestinal parasites and pregnancy. Infect Dis Obstet Gynecol. 1985;66(5):639-43.

131. Dao AH, Gregory DW, McKee LC. Specific health problems of Southeast Asian refugees in middle Tennessee. Southern Medical Journal. 1984;77(8):995-7.

132. DeGirolami PC, Kimber J. Intestinal parasites among Southeast Asian refugees in Massachusetts. Am J Clin Pathol. 1983;79(4):502-4.

133. Doganay M, Demiraslan H. Refugees of the Syrian Civil War: Impact on reemerging infections, health services, and biosecurity in Turkey. Health Secur. 2016;14(4):220-5.

134. Duffy PE, Le Guillouzic H, Gass RF, Innis BL. Murine typhus identified as a major cause of febrile illness in a camp for displaced Khmers in Thailand. Am J Trop Med Hyg. 1990;43(5):520-6.

135. Dunya G, Habib R, Moukarbel RV, Khalifeh I. Head and neck cutaneous leishmania: clinical characteristics, microscopic features and molecular analysis in a cohort of 168 cases. Eur Arch Otorhinolaryngol. 2016;273(11):3819-26.

136. Eksi F, Ozgoztasi O, Karsligil T, Saglam M. Genotyping Leishmania promastigotes isolated from patients with cutaneous leishmaniasis in south-eastern Turkey. Journal of International Medical Research. 2016;45(1):114-22.

137. El Hajj R, El Hajj H, Khalifeh I. Fatal visceral Leishmaniasis caused by Leishmania infantum, Lebanon. Emerg Infect Dis. 2018;24(5):906-7.

138. El Safadi D, Merhabi S, Rafei R, Mallat H, Hamze M, Acosta-Serrano A. Cutaneous leishmaniasis in north Lebanon: Re-emergence of an important neglected tropical disease. Trans R Soc Trop Med Hyg. 2019;113(8):471-6.

139. Engström ELS, Salih GN, Wiese L. Seronegative, complicated hydatid cyst of the lung: A case report. Respir Med Case Rep. 2017;21:96-8.

140. Eroglu F, Ozgoztasi O. The increase in neglected cutaneous leishmaniasis in Gaziantep province of Turkey after mass human migration. Acta Trop. 2019;192:138-43.

141. Fan CK, Liao CW, Wu MS, Su KE, Han BC. Seroepidemiology of Toxoplasma gondii infection among Chinese aboriginal and Han people residing in mountainous areas of northern Thailand. J Parasitol. 2003;89(6):1239-42.

142. Franco-Paredes C, Nicolls D, Kempker R, Dismukes R, Kozarsky P. Pelvic echinococcosis in a Northern Iraqi refugee. J Travel Med. 2006;13(2):119-22.

143. Fritzsche M, Gottstein B, Wigglesworth MC, Eckert J. Serological survey of human cysticercosis in Irianese refugee camps in Papua New Guinea. Acta Tropica. 1990;47(2):69-77.

144. Goswami ND, Shah JJ, Corey GR, Stout JE. Short report: Persistent eosinophilia and Strongyloides infection in Montagnard refugees after presumptive albendazole therapy. Am J Trop Med Hyg. 2009;81(2):302-4.

145. Grunow R, Jacob D, Klee S, Schlembach D, Jackowski-Dohrmann S, Loenning-Baucke V, et al. Brucellosis in a refugee who migrated from Syria to Germany and lessons learnt, 2016. Eurosurveillance. 2016;21(31):5-8.

146. Gurses G, Ozaslan M, Zeyrek FY, Kilic IH, Doni NY, Karagoz ID, et al. Molecular identification of Leishmania spp. isolates causes cutaneous leishmaniasis (CL) in Sanliurfa Province, Turkey, where CL is highly endemic. Folia Microbiol (Praha). 63(3):353-9.

147. Gyorkos TW, Frappier-Davignon L, MacLean JD, Viens P. Effect of screening and treatment on imported intestinal parasite infections: Results from a randomized, controlled trial. Am J Epidemiol. 1989;129(4):753-61.

148. Gyorkos TW, MacLean JD, Viens P, Chheang C, Kokoskin-Nelson E. Intestinal parasite infection in the Kampuchean refugee population 6 years after resettlement in Canada. Infect Dis. 1992;166(2):413-7.

149. Halici-Ozturk F, Yakut K, Öcal FD, Erol A, Gökay S, Çağlar AT, et al. Seroprevalence of Toxoplasma gondii infections in Syrian pregnant refugee women in Turkey. European Journal of Obstetrics and Gynecology and Reproductive Biology. 2021;256:91-4.

150. Hassan AO, Mero WMS. Prevalence of intestinal parasites among displaced people living in displacement camps in duhok province/Iraq. Internet Journal of Microbiology. 2020;17(1).

151. Heendeniya A, Bogoch I. Multiple Hepatic Hydatid Cysts in an Iraqi Refugee. Am J Trop Med Hyg. 2018;99(5):1107.

152. Hijawi KJF, Hijjawi NS, Ibbini JH. Detection, genotyping, and phylogenetic analysis of Leishmania isolates collected from infected Jordanian residents and Syrian refugees who suffered from cutaneous leishmaniasis. Parasitol Res. 2019;118(3):793-805.

153. Hoffman SL, Barrett-Connor E, Norcross W, Nguyen D. Intestinal parasites in Indochinese immigrants. Am J Trop Med Hyg. 1981;30(2):340-3.

154. Hofstetter M, Nash TE, Cheever AW. Infection with Schistosoma mekongi in Southeast Asian refugees. J Infect Dis. 1981;144(5):420-6.

155. Hussain M, Munir S, Jamal MA, Ayaz S, Akhoundi M, Mohamed K. Epidemic outbreak of anthroponotic cutaneous leishmaniasis in Kohat District, Khyber Pakhtunkhwa, Pakistan. Acta Tropica. 2017;172:147-55.

156. Inci R, Ozturk P, Mulayim MK, Ozyurt K, Alatas ET, Inci MF. Effect of the Syrian civil war on prevalence of cutaneous Leishmaniasis in Southeastern Anatolia, Turkey. Medical Science Monitor. 2015;21:5.

157. Jamal Q, Shah A, Ali N, Ashraf M, Awan MM, Lee CM. Prevalence and comparative analysis of cutaneous Leishmaniasis in Dargai Region in Pakistan. Pakistan Journal of Zoology. 2013;45(2):537-41.

158. Jensenius M, Hoiby EA, Berild D, Stiris M, Ringertz SH. Difficulties in diagnosing Brucella spondylitis. Scand J Infect Dis. 2000;32(4):425-6.

159. Jones MJ, Thompson Jr JH, Brewer NS. Infectious diseases of Indochinese refugees. Mayo Clin Proc. 1980;55(8):482-8.

160. Kanani K, Amr ZS, Shadfan B, Khorma R, Rø G, Abid M, et al. Cutaneous leishmaniasis among Syrian refugees in Jordan. Acta Tropica. 2019;194:169-71.

161. Keittivuti B, D'Agnes T, Keittivuti A, Viravaidya M. Prevalence of schistosomiasis and other parasitic diseases among Cambodian refugees residing in Bang-Kaeng holding center, Prachinburi Province, Thailand. Am J Trop Med Hyg. 1982;31(5):988-90.

162. Keittivuti B, Keittivuti A, D'Agnes T. Schistosomiasis in Cambodian refugees at Ban-Kaeng holding centre, Prachinburi province, Thailand. Southeast Asian J Trop Med Public Health. 1982;13(2):216-9.

163. Keittivuti B, Keittivuti A, O'Rourke T, D'Agnes T. Treatment of Schistosoma mekongi with praziquantel in Cambodian refugees in holding centres in Prachinburi Province, Thailand. Trans R Soc Trop Med Hyg. 1984;78(4):477-9.

164. Keittivuti B, Keittivuti A, O'Rourke TF. Parasitic diseases with emphasis on schistosomiasis in Cambodian refugees, in Prachinburi Province Thailand. Southeast Asian J Trop Med Public Health. 1983;14(4):491-4.

165. Khan MI, Muhammad M, Khan W, Khan N, Noor SM. Nasal involvement in cutaneous leishmaniasis. Journal of Postgraduate Medical Institute. 2010;24(3):202-6.

166. Kolaczinski J, Brooker S, Reyburn H, Rowland M. Epidemiology of anthroponotic cutaneous leishmaniasis in Afghan refugee camps in northwest Pakistan. Transactions of the Royal Society of Tropical Medicine and Hygiene. 2004;98(6):373-8.

167. Lin CY, Chen TC, Dai CY, Yu ML, Lu PL, Yen JH, et al. Serological investigation to identify risk factors for post-flood infectious diseases: a longitudinal survey among people displaced by Typhoon Morakot in Taiwan. BMJ Open. 2015;5(5):e007008.

168. Lindner AK, Richter J, Gertler M, Nikolaus M, Martinez GE, Muller K, et al. Cutaneous leishmaniasis in refugees from Syria: complex cases in Berlin 2015-2020. Journal of Travel Medicine. 2020;27(7):8.

169. McAuley JB, Michelson MK, Hightower AW, Engeran S, Wintermeyer LA, Schantz PM. A trichinosis outbreak among Southeast Asian refugees. Am J Epidemiol. 1992;135(12):1404-10.

170. McCleery EJ, Patchanee P, Pongsopawijit P, Chailangkarn S, Tiwananthagorn S, Jongchansittoe P, et al. Taeniasis among refugees living on Thailand–Myanmar border, 2012. Emerg Infect Dis. 2015;21(10):1824-6.

171. McGready R, Ashley EA, Wuthiekanun V, Tan SO, Pimanpanarak M, Viladpai-Nguen SJ, et al. Arthropod borne disease: The leading cause of fever in pregnancy on the thai-burmese border. PLoS Negl Trop Dis. 2010;4(11).

172. Mitchell T, Lee D, Weinberg M, Phares C, James N, Amornpaisarnloet K, et al. Impact of enhanced health interventions for United States-bound refugees: Evaluating best practices in migration health. Am J Trop Med Hyg. 2018;98(3):920-8.

173. Moaven L, Van Asten M, Crofts N, Locarnini SA. Seroepidemiology of hepatitis E in selected Australian populations. J Med Virol. 1995;45(3):326-30.

174. Mockenhaupt FP, Barbre KA, Jensenius M, Larsen CS, Barnett ED, Stauffer W, et al. Profile of illness in syrian refugees: A geosentinel analysis, 2013 to 2015. Eurosurveillance. 2016;21(10).

175. Molina CD, Molina MM, Molina JM. Intestinal parasites in Southeast Asian refugees two years after immigration. West J Med. 1988;149(4):422-5.

176. Motamedi MH, Har, i P, Azizi T. Leishmaniasis of the face: Report of a case. Indian Journal of Dermatology. 2009;54(5):S37-S40.

177. Mumtaz N, Saqulain G, Mumtaz N. Dilemma of health rights of vulnerable citizens: A narrative review. J Pak Med Assoc. 2021;71(12):2782-6. doi: 10.47391/jpma.2078.

178. Nash TE, Hofstetter M, Cheever AW, Ottesen EA. Treatment of Schistosoma mekongi with praziquantel: a double-blind study. Am J Trop Med Hyg. 1982;31(5):977‐82.

179. Ntais P, Christodoulou V, Tsirigotakis N, Dokianakis E, Dedet J-P, Pratlong F, et al. Will the introduction of Leishmania tropica MON-58, in the island of Crete, lead to the settlement and spread of this rare zymodeme? Acta Tropica. 2014;132:125-30. doi:10.1016/j.actatropica.2014.01.003.

180. O'Neal SE, Robbins NM, Townes JM. Neurocysticercosis among resettled refugees from Burma. J Travel Med. 2012;19(2):118-21.

181. Parish RA. Intestinal parasites in Southeast Asian refugee children. West J Med. 1985;143(1):47-9.

182. Paxton GA, Sangster KJ, Maxwell EL, McBride CRJ, Drewe RH. Post-arrival health screening in Karen refugees in Australia. PLoS One. 2012;7(5).

183. Qazi M, Weimer AC, Bedard BA, Kennedy BS. Q-fever in a refugee after exposure to a central New York State livestock farm. Annals of Tropical Medicine and Public Health. 2016;9(4):266-70.

184. Rab MA, al Rustamani L, Bhutta RA, Mahmood MT, Evans DA. Cutaneous leishmaniasis: iso-enzyme characterisation of Leishmania tropica. J Pak Med Assoc. 1997;47(11):270-3.

185. Relić T, Kačarević H, Ilić N, Jovanović D, Tambur Z, Doder R, et al. Intestinal parasitosis in asylum seekers from the middle east and South Asia. Vojnosanitetski pregled. 2018;75(11):1101-5.

186. Rowland M, Munir A, Durrani N, Noyes H, Reyburn H. An outbreak of cutaneous leishmaniasis in an Afghan refugee settlement in north-west Pakistan. J Transactions of the Royal Society of Tropical Medicine and Hygiene. 1999;93(2):133-6.

187. Saab M, El Hage H, Charafeddine K, Habib RH, Khalifeh I. Diagnosis of cutaneous leishmaniasis: Why punch when you can scrape? Am J Trop Med Hyg. 2015;92(3):518-22.

188. Saroufim M, Charafeddine K, Issa G, Khalifeh H, Habib RH, Berry A, et al. Ongoing epidemic of cutaneous Leishmaniasis among Syrian refugees, Lebanon. Emerg Infect Dis. 2014;20(10):1712-5.

189. Sencan I, Sahin I, Kaya D, Oksuz S, Yildirim M. Assessment of HAV and HEV seroprevalence in children living in post-earthquake camps from Düzce, Turkey. Eur J Epidemiol. 2004;19(5):461-5.

190. Shen C, Li S, Zheng S, Choi MH, Bae YM, Hong ST. Tissue parasitic helminthiases are prevalent at Cheongjin, North Korea. Korean J Parasitol. 2007;45(2):139-44.

191. Spicher VM, Genin B, Jordan AR, Rubbia-Brandt L, Le Coultre C. Peritoneal schistosomiasis: an unusual laparoscopic finding. J Pediatr Surg. 2004;39(4):631-3.

192. Stehr-Green JK, Schantz PM. Trichinosis in Southeast Asian refugees in the United States. Am J Public Health. 1986;76(10):1238-9.

193. Storer E, Wayte J. Cutaneous leishmaniasis in Afghani refugees. Australasian Journal of Dermatology. 2005;46(2):80-3.

194. Sullivan R, Linneman Jr CC, Clark CS, Walzer PD. Seroepidemiologic study of giardiasis patients and high-risk groups in a midwestern city in the United States. Am J Public Health. 1987;77(8):960-3.

195. Tappe D, Weise D, Ziegler U, Müller A, Müllges W, Stich A. Brain and lung metastasis of alveolar echinococcosis in a refugee from a hyperendemic area. J Med Microbiol. 2008;57(11):1420-3.

196. Taylor DN, Echeverria P, Pitarangsi C, Seriwatana J, Sethabutr O, Bodhidatta L, et al. Application of DNA hybridization techniques in the assessment of diarrheal disease among refugees in Thailand. Am J Epidemiol. 1988;127(1):179-87.

197. Temcharoen P, Viboolyavatana J, Tongkoom B. A survey on intestinal parasitic infections in Laotian refugees at Ubon Province, northeastern Thailand, with special reference to schistosomiasis. Southeast Asian J Trop Med Public Health. 1979;10(4):552-5.

198. Tittle BS, Harris JA, Chase PA. Health screening of Indochinese refugee children. Am J Dis Child. 1982;136(8):697-700.

199. Ul Haq KA, Gul NA, Muhammad Hammad H, Bibi Y, Bibi A, Mohsan J. Prevalence of giardia intestinalis and hymenolepis nana in afghan refugee population of mianwali district, pakistan. African Health Sciences. African Health Sciences. 2015;15(2):394-400.

200. Um J, Nam Y, Lim JN, Kim M, An Y, Hwang SH, et al. Seroprevalence of scrub typhus, murine typhus and spotted fever groups in North Korean refugees. International Journal of Infectious Diseases. 2021;106:23-8.

201. Van Enter BJD, Lau YL, Ling CL, Watthanaworawit W, Sukthana Y, Lee WC, et al. Seroprevalence of toxoplasma gondii infection in refugee and migrant pregnant women along the Thailand-myanmar border. Am J Trop Med Hyg. 2017;97(1):232-5.

202. Van Kesteren L, Maniewski U, Bottieau E, Cnops L, Huits R. Cutaneous leishmaniasis in syrian refugee children: A case series. Pediatr Infect Dis J. 2020:E154-E6.

203. Wiesenthal AM, Nickels MK, Hashimoto KG. Intestinal parasites in Southeast-Asian refugees. Prevalence in a community of Laotians. JAMA. 1980;244(22):2543-4.

204. Wollina U, Koch A, Guarneri C, Tchernev G, Lotti T. Cutaneous leishmaniasis – A case series from Dresden. Open Access Maced J Med Sci. 2018;6(1):89-92.

205. Yangco BG, Vincent AL, Vickery AC. A survey of filariasis among refugees in South Florida. Am J Trop Med Hyg. 1984;33(2):246-51.

206. Yasar AS, Karaman K, Geylan H, Cetin M, Guven B, Oner AF. Typhoid fever accompanied with hematopoetic lymphohistiocytosis and rhabdomyolysis in a refugee child. Journal of Pediatric Hematology Oncology. 2019;41(4):E233-E4.

207. Yasin AM, Esa HAH, Hameed AA, Wahid W, Ahamed POS. First case of pulmonary hydatid cyst in a pregnant syrian refugee woman in malaysia. Med J Malaysia. 2021;76(1):103-6.

208. Yeaney GA, Kolar BS, Silberstein HJ, Wang HZ. Case 163: Solitary neurocysticercosis. Radiology. 2010;257(2):581-5.

209. Yentur Doni N, Gurses G, Dikme R, Aksoy M, Yildiz Zeyrek F, Simsek Z, et al. Cutaneous leishmaniasis due to three Leishmania species among Syrian refugees in Sanliurfa, Southeastern Turkey. Acta Parasitol. 2020;65(4):936-48.

210. Yildirim C, Arda B, Uz I, Uyar M, Ersel M, Yamazhan T, et al. Wars do not kill only with guns: A case of rabies in a Syrian refugee. Mediterranean Journal of Infection Microbes and Antimicrobials. Eur J Epidemiol. 2017;6:2.

211. Zahedi D, Moori P, Ashraf I, Hafeez I. Pleuritic chest pain in a young asylum seeker. Breathe (Sheff). 2020;16(1):190294.

212. Abu Mourad TA. Palestinian refugee conditions associated with intestinal parasites and diarrhoea: Nuseirat refugee camp as a case study. Public Health. 2004;118(2):131-42.

213. Aksin S, Cim N, Andan C, Tunc S, Goklu MR. Comparison of obstetric and infectious results among Syrian pregnant women. Annals of Clinical and Analytical Medicine. 2021;12(5):501-5. doi:10.4328/acam.20411.

214. Al-Hatamleh MAI, Hatmal MM, Mustafa SHF, Alzu'bi M, AlSou'b AF, Abughanam SNS, et al. Experiences and perceptions of COVID-19 infection and vaccination among Palestinian refugees in Jerash camp and Jordanian citizens: a comparative cross-sectional study by face-to-face interviews. Infectious diseases of poverty. 2022;11(1):123. doi:10.1186/s40249-022-01047-y.

215. Altare C, Kostandova N, Okeeffe J, Hayek H, Fawad M, Musa Khalifa A, et al. COVID-19 epidemiology and changes in health service utilization in Azraq and Zaatari refugee camps in Jordan: A retrospective cohort study. PLoS Medicine. 2022;19(5). doi:10.1371/journal.pmed.1003993.

216. Altinel Y, Tas B. How to predict the diagnosis of cutaneous leishmaniasis in a non-endemic region. Indian Journal of Dermatology. 2022;67(3):232-8. doi:10.4103/ijd.IJD_452_20.

217. Azlin MY, Esa HAH, Hameed AA, Wahid W, Pakeer O. First case of pulmonary hydatid cyst in a pregnant Syrian refugee woman in Malaysia. Med J Malaysia. 2021;76(1):103-6.

218. Balakrishnan VS. Impact of COVID-19 on migrants and refugees. The Lancet Infectious diseases. 2021;21(8):1076-7. doi:10.1016/S1473-3099(21)00410-2.

219. Debus D, Genç S, Kurz P, Holzer M, Bauer K, Heimke-Brinck R, et al. Case Report: Local treatment of a Leishmania tropica infection in a Syrian child with a novel filmogenic preparation of pharmaceutical sodium chlorite. Am J Trop Med Hyg. 2022;106(3):857-60. doi:10.4269/ajtmh.21-0962.

220. Hammoud S, Onchonga D, Amer F, Kocsis B. The burden of communicable diseases in Lebanon: Trends in the past decade. Disaster Medicine and Public Health Preparedness. 2022;16(5):1725-7. doi:10.1017/dmp.2021.200.

221. Hanapi IRM, Sahimin N, Maackara MJB, Annisa AS, Mutalib R, Lewis JW, et al. Prevalence of anti-Leptospira antibodies and associated risk factors in the Malaysian refugee communities. BMC Infect Dis. 2021;21(1). doi:10.1186/s12879-021-06830-0.

222. Kalani N, Hatami N, Haghbeen M, Yaqoob U, Raeyat Doost E. Covid-19 health care for afghan refugees as a minor ethnicity in iran; clinical differences and racial equality in health. Acta Medica Iranica. 2021;59(8):466-71.

223. Khachfe HH, Zayyoun FJ, Sharif-Askari E, Ramadan W, Hallal N, Khachfe HM. Effect of leishmaniasis on the performance of elementary school students: A case study among syrian refugees in some bekaa (lebanon) area schools. 2019;9(4):266-73.

224. Kheirallah KA, Ababneh BF, Bendak H, Alsuwaidi AR, Elbarazi I. Exploring the mental, social, and lifestyle effects of a positive COVID-19 infection on Syrian refugees in Jordan: A qualitative study. International Journal of Environmental Research and Public Health. 2022;19(19). doi:10.3390/ijerph191912588.

225. Barbier D, Demenais F, Lefait JF, David B, Blanc M, Hors J, et al. Susceptibility to human cutaneous leishmaniasis and HLA, Gm, Km markers. Tissue Antigens. 1987;30(2):63-7.

226. Enkelmann J, Stark K, Faber M. Epidemiological trends of notified human brucellosis in Germany, 2006–2018. Int J Infect Dis. 2020;93:353-8.

227. Francke E. Medical evaluation of Indochinese refugees conditions to consider. Postgraduate Medicine. 1982;72(5):92-3.

228. Khan S, Akbar SMF, Kimitsuki K, Saito N, Yahiro T, Al Mahtab M, et al. Recent downhill course of COVID-19 at Rohingya refugee camps in Bangladesh: Urgent action solicited. J Glob Health. 2021;11:03097. doi: 10.7189/jogh.11.03097.

229. Mazhar MKA, Finger F, Evers ES, Kuehne A, Ivey M, Yesurajan F, et al. An outbreak of acute jaundice syndrome (AJS) among the Rohingya refugees in Cox’s Bazar, Bangladesh: Findings from enhanced epidemiological surveillance. PLoS One. 2021;16(4). doi:10.1371/journal.pone.0250505.

230. McDowell D, Harper CG. Neurocysticercosis - Two Australian cases. Med J Aust. 1990;152(4):217-8.

231. Rahim M, Kazi BM, Bile KM, Munir M, Khan AR. The impact of the disease early warning system in responding to natural disasters and conflict crises in Pakistan. Eastern Mediterranean Health Journal. 2010;16:S114-21. doi:10.26719/2010.16.supp.114.

232. Samuda GM, Chan SP, Yeung CY. Vietnamese child health in a Hong Kong closed camp. Aust Paediatr J. 1988;24(2):115-7.

233. Southwood T, Davidson GP, Phillips GE, Rice M. Hepatosplenic schistosomiasis in a South-East Asian refugee child in South Australia. Aust N Z J Med. 1983;13(4):384-6.

234. Tolunay O, Çelik Ü, Arslan I, Tutun B, Özkaya M. Evaluation of clinical findings and treatment results of Coronavirus disease 2019 (COVID-19) in pediatric cancer patients: A single center experience. Frontiers in Pediatrics. 2022;10. doi:10.3389/fped.2022.848379.

235. Webster JL, Stauffer WM, Mitchell T, Lee D, O’Connell EM, Weinberg M, et al. Cross-sectional assessment of the association of eosinophilia with intestinal parasitic infection in U.S.-bound refugees in Thailand: Prevalent, age dependent, but of limited clinical utility. Am J Trop Med Hyg. 2022;106(5):1552-9. doi:10.4269/ajtmh.21-0853.

236. Yan XY, Xiao W, Zhou SP, Wang XC, Wang ZK, Zhao MC, et al. A four-generation family transmission chain of COVID-19 along the China-Myanmar border in October to November 2021. Frontiers in Public Health. 2022;10. doi:10.3389/fpubh.2022.1004817.

237. Chaves NJ, Gibney KB, Leder K, O'Brien DP, Marshall C, Biggs BA. Screening practices for infectious diseases among Burmese refugees in Australia. Emerg Infect Dis. 2009;15(11):1769-72. doi: 10.3201/eid1511.090777.

238. Koçarslan S, Turan E, Ekinci T, Yesilova Y, Apari R. Clinical and histopathological characteristics of cutaneous Leishmaniasis in Sanliurfa City of Turkey including Syrian refugees. Indian J Pathol Microbiol. 2013;56(3):211-5. doi: 10.4103/0377-4929.120367.

239. Lurio J, Verson H, Karp S. Intestinal parasites in Cambodians: comparison of diagnostic methods used in screening refugees with implications for treatment of populations with high rates of infestation. J Am Board Fam Pract. 1991;4(2):71-8.

240. Saikal SL, Ge L, Mir A, Pace J, Abdulla H, Leong KF, et al. Skin disease profile of Syrian refugees in Jordan: a field-mission assessment. J Eur Acad Dermatol Venereol. 2020;34(2):419-25. doi: 10.1111/jdv.15909.

241. Sharara SL, Kanj SS. War and infectious diseases: challenges of the Syrian civil war. PLoS Pathog. 2014;10(10):e1004438. doi: 10.1371/journal.ppat.1004438.

242. Jacoby H, Rawling RA, Granato PA. Cutaneous leishmaniasis in a Central American refugee. Clinical Microbiology Newsletter. 2014;36(3):22-4.

243. Rodríguez-Morales AJ, Bonilla-Aldana DK, Bonilla-Aldana JC, Mondragón-Cardona Á. Arboviral diseases among internally displaced people of Neiva, Colombia, 2015-2017. Travel Med Infect Dis. 2019;26(2).

244. Schroeder Jr HW, Yarrish RL, Perkins TF, Lee C. Sequential disseminated tuberculosis and toxoplasmosis in a Haitian refugee. Southern Medical Journal. 1984;77(4):533-4.

245. Bojorquez-Chapela I, Strathdee SA, Garfein RS, Benson CA, Chaillon A, Ignacio C, et al. The impact of the COVID-19 pandemic among migrants in shelters in Tijuana, Baja California, Mexico. BMJ Global Health. 2022;7(3). doi:10.1136/bmjgh-2021-007202.

246. da Costa e Silva GR, Martins TLS, de Almeida Silva C, Caetano KAA, dos Santos Carneiro MA, Silva BVDE, et al. Hepatitis A and E among immigrants and refugees in Central Brazil. Revista de Saude Publica. 2022;56. doi:10.11606/S1518-8787.2022056003839.

247. da Silva HP, Abreu IN, Lima CNC, de Lima ACR, Barbosa AD, de Oliveira LR, et al. Migration in times of pandemic: SARS-CoV-2 infection among the Warao indigenous refugees in Belem, Para, Amazonia, Brazil. BMC Public Health. 2021;21(1). doi:10.1186/s12889-021-11696-7.

248. Pham PN, Keegan K, Johnston LG, Rodas J, Restrepo MA, Wei C, et al. Assessing the impact of the COVID-19 pandemic among Venezuelan refugees and migrants in Colombia using respondent-driven sampling (RDS). BMJ Open. 2022;12(10). doi:10.1136/bmjopen-2021-054820.

249. Vallejo-Janeta AP, Morales-Jadan D, Freire-Paspuel B, Lozada T, Cherrez-Bohorquez C, Garcia-Bereguiain MA, et al. COVID-19 outbreaks at shelters for women who are victims of gender-based violence from Ecuador. International Journal of Infectious Diseases. 2021;108:531-6. doi:10.1016/j.ijid.2021.06.012.

250. Steele LS, MacPherson DW, Kim J, Keystone JS, Gushulak BD. The sero-prevalence of antibodies to Trypanosoma cruzi in Latin American refugees and immigrants to Canada. Journal of Immigrant and Minority Health. 2007;9(1):43-7. doi: 10.1007/s10903-006-9014-x.
