# Supplementary material for: Status of zoonotic disease research in refugees, asylum seekers and internally displaced people, globally: A scoping review of forty clinically important zoonotic pathogens
Source: PLoS Negl Trop Dis. 2024 May 20;18(5):e0012164. doi: 10.1371/journal.pntd.0012164 (PMC11142688; doi:10.1371/journal.pntd.0012164)
Supplement: S9 Table — (DOCX) [file pntd.0012164.s011.docx]

**S9 Table:** Publications included in the scoping review reporting on zoonotic pathogens associated with disease outbreaks

| Pathogen | References |
| --- | --- |
| Hepatitis E virus | [1-15] |
| SARS CoV-2 | [16-27] |
| Leishmania spp. | [6, 28-37] |
| Dengue virus | [6, 38-40] |
| Salmonella enterica | [6, 41, 42] |
| CCHF virus | [38, 39] |
| Rift Valley Fever virus | [6, 39] |
| West Nile virus | [38, 39] |
| Yellow Fever virus | [6, 39] |
| Borrelia recurrentis | [6] |
| Chikungunya virus | [39] |
| Ebola virus | [6] |
| Lassa virus | [6] |
| Marburg virus | [6] |
| Rickettsia spp. | [43] |
| Trichinella spp. | [44] |
| Trypanosoma spp. | [6] |
| Zika virus | [39] |

**References**

1. Enterically transmitted non-A, non-B hepatitis--East Africa. MMWR. 1987;36(16):241-4.

2. Hepatitis E, Chad. Weekly epidemiological record / Health Section of the Secretariat of the League of Nations. 2004;79(35):313.

3. Ahmed JA, Moturi E, Spiegel P, Schilperoord M, Burton W, Kassim NH, et al. Hepatitis E outbreak, Dadaab refugee camp, Kenya, 2012. Emerg Infect Dis. 2013;19(6):1010-2.

4. Boccia D, Guthmann JP, Klovstad H, Hamid N, Tatay M, Ciglenecki I, et al. High mortality associated with an outbreak of hepatitis E among displaced persons in Darfur, Sudan. Clinical Infectious Diseases. 2006;42(12):1679-84.

5. Browne LB, Menkir Z, Kahi V, Maina G, Asnakew S, Tubman M, et al. Hepatitis E outbreak among refugees from South Sudan - Gambella, Ethiopia, April 2014-January 2015. MMWR. 2015;64(19):537-.

6. Desai AN, Ramatowski JW, Marano N, Madoff LC, Lassmann B. Infectious disease outbreaks among forcibly displaced persons: An analysis of ProMED reports 1996-2016. Conflict and Health. 2020;14(1).

7. Lagare A, Ibrahim A, Ousmane S, Issaka B, Zaneidou M, Kadadé G, et al. Outbreak of Hepatitis E virus infection in displaced persons camps in Diffa region, Niger, 2017. Am J Trop Med Hyg. 2018;99(4):1055-7.

8. Mérens A, Guérin PJ, Guthmann JP, Nic, E. Outbreak of hepatitis E virus infection in Darfur, Sudan: Effectiveness of real-time reverse transcription-PCR analysis of dried blood spots. J Clin Microbiol. 2009;47(6):1931-3.

9. Nicand E, Armstrong GL, Enouf V, Guthmann JP, Guerin JP, Caron M, et al. Genetic heterogeneity of hepatitis E virus in Darfur, Sudan, and neighboring Chad. Journal of Medical Virology. 2005;77(4):519-21.

10. Thomson K, Luis Dvorzak J, Lagu J, Laku R, Dineen B, Schilperoord M, et al. Investigation of hepatitis E outbreak among refugees - Upper Nile, South Sudan, 2012-2013. MMWR. 2013;62(29):581-6.

11. Ahmed A, Ali Y, Siddig EE, Hamed J, Mohamed NS, Khairy A, et al. Hepatitis E virus outbreak among Tigray war refugees from Ethiopia, Sudan. Emerg Infect Dis. 2022;28(8):1722-4. doi:10.3201/eid2808.220397.

12. Desai AN, Mohareb AM, Elkarsany MM, Desalegn H, Madoff LC, Lassmann B. Viral hepatitis E outbreaks in refugees and internally displaced populations, sub-Saharan Africa, 2010–2020. Emerg Infect Dis. 2022;28(5):1074-6. doi:10.3201/eid2805.212546.

13. Guthmann JP, Klovstad H, Boccia D, Hamid N, Pinoges L, Nizou JY, et al. A large outbreak of hepatitis E among a displaced population in Darfur, Sudan, 2004: the role of water treatment methods. Clin Infect Dis. 2006;42(12):1685-91. doi: 10.1086/504321.

14. IsaÃ¤cson M, Frean J, He J, Seriwatana J, Innis BL. An outbreak of hepatitis E in Northern Namibia, 1983. Am J Trop Med Hyg. 2000;62(5):619-25. doi: 10.4269/ajtmh.2000.62.619.

15. Teshale EH, Grytdal SP, Howard C, Barry V, Kamili S, Drobeniuc J, et al. Evidence of person-to-person transmission of hepatitis E virus during a large outbreak in Northern Uganda. Clinical Infectious Diseases. 2010;50(7):1006-10. doi: 10.1086/651077.

16. Baggett TP, Racine MW, Lewis E, De Las Nueces D, O’Connell JJ, Bock B, et al. Addressing COVID-19 among people experiencing homelessness: Description, adaptation, and early findings of a multiagency response in Boston. Public Health Rep. 2020;135(4):435-41.

17. Le Bihan C, Faucherre V, Le Moing V, Mehenni A, Nantes D, Da Silva A, et al. COVID-19: The forgotten cases of hidden exiles. Infect Dis Now. 2021.

18. Redditt V, Wright V, Rashid M, Male R, Bogoch I. Outbreak of SARS-CoV-2 infection at a large refugee shelter in Toronto, April 2020: a clinical and epidemiologic descriptive analysis. CMAJ Open. 2020;8(4):E819-e24.

19. Turunen T, Kontunen K, Sugulle K, Hieta P, Snellman O, Hussein I, et al. COVID-19 outbreak at a reception centre for asylum seekers in Espoo, Finland. J Migr Health. 2021;3:100043.

20. Dressler A, Finci I, Wagner-Wiening C, Eichner M, Brockmann SO. Epidemiological analysis of 3,219 COVID-19 outbreaks in the state of Baden-Wuerttemberg, Germany. Epidemiology and Infection. 2021. doi:10.1017/S0950268821000911.

21. Fabris S, d'Ettorre G, Spagnolello O, Russo A, Lopalco M, D'Agostino F, et al. SARS-CoV-2 among migrants recently arrived in Europe from low- and middle-income countries: Containment strategies and special features of management in reception centers. Frontiers in Public Health. 2021;9. doi:10.3389/fpubh.2021.735601.

22. Gignoux E, Athanassiadis F, Yarrow AG, Jimale A, Mubuto N, Déglise C, et al. Seroprevalence of SARS-CoV-2 antibodies and retrospective mortality in a refugee camp, Dagahaley, Kenya. PLoS One. 2021;16(12). doi:10.1371/journal.pone.0260989.

23. Kheirallah KA, Ababneh BF, Bendak H, Alsuwaidi AR, Elbarazi I. Exploring the mental, social, and lifestyle effects of a positive COVID-19 infection on Syrian refugees in Jordan: A Qualitative Study. International Journal of Environmental Research and Public Health. 2022;19(19). doi:10.3390/ijerph191912588.

24. Knust B, Wongjindanon N, Moe AA, Herath L, Kaloy W, Soe TT, et al. Enhancing respiratory disease surveillance to detect COVID-19 in shelters for displaced persons, Thailand-Myanmar Border, 2020-2021. Emerg Infect Dis. 2022;28(13):S17-s25. doi:10.3201/eid2813.220324.

25. Kondilis E, Papamichail D, McCann S, Carruthers E, Veizis A, Orcutt M, et al. The impact of the COVID-19 pandemic on refugees and asylum seekers in Greece: A retrospective analysis of national surveillance data from 2020. EClinicalMedicine. 2021;37. doi:10.1016/j.eclinm.2021.100958.

26. Palacios CF, Tucker EW, Travassos MA. Coronavirus disease 2019 burden among unaccompanied minors in US custody. Clinical Infectious Diseases. 2022. doi:10.1093/cid/ciac636.

27. Khan S, Akbar SMF, Kimitsuki K, Saito N, Yahiro T, Al Mahtab M, et al. Recent downhill course of COVID-19 at Rohingya refugee camps in Bangladesh: Urgent action solicited. J Glob Health. 2021;11:03097. doi: 10.7189/jogh.11.03097.

28. Alawieh A, Musharrafieh U, Jaber A, Berry A, Ghosn N, Bizri AR. Revisiting leishmaniasis in the time of war: the Syrian conflict and the Lebanese outbreak. International Journal of Infectious Diseases. 2014;29:115-9.

29. Hussain M, Munir S, Jamal MA, Ayaz S, Akhoundi M, Mohamed K. Epidemic outbreak of anthroponotic cutaneous leishmaniasis in Kohat District, Khyber Pakhtunkhwa, Pakistan. Acta Tropica. 2017;172:147-55.

30. Kolaczinski J, Brooker S, Reyburn H, Rowland M. Epidemiology of anthroponotic cutaneous leishmaniasis in Afghan refugee camps in northwest Pakistan. J Transactions of the Royal Society of Tropical Medicine and Hygiene. 2004;98(6):373-8.

31. Rowland M, Munir A, Durrani N, Noyes H, Reyburn H. An outbreak of cutaneous leishmaniasis in an Afghan refugee settlement in north-west Pakistan. J Transactions of the Royal Society of Tropical Medicine and Hygiene. 1999;93(2):133-6.

32. Saroufim M, Charafeddine K, Issa G, Khalifeh H, Habib RH, Berry A, et al. Ongoing epidemic of cutaneous Leishmaniasis among Syrian Refugees, Lebanon. Emerg Infect Dis. 2014;20(10):1712-5.

33. Sulaiman AA, Elmadhoun WM, Noor SK, Bushara SO, Almobarak AO, Awadalla H, et al. An outbreak of cutaneous leishmaniasis among a displaced population in North Sudan: Review of cases. J Family Med Prim Care. 2019;8(2):556-63.

34. Zijlstra EE, Ali MS, El-Hassan AM, El-Toum IA, Satti M, Ghalib Kager HWPA. Direct agglutination test for diagnosis and sero- epidemiological survey of kala-azar in the Sudan. Trans R Soc Trop Med Hyg. 1991;85(4):474-6.

35. Zijlstra EE, Siddig Ali M, El-Hassan AM, El-Toum IA, Satti M, Ghalib HW, et al. Kala-azar in displaced people from southern Sudan: Epidemiological, clinical and therapeutic findings. Trans R Soc Trop Med Hyg. 1991;85(3):365-9.

36. Hammoud S, Onchonga D, Amer F, Kocsis B. The Burden of Communicable Diseases in Lebanon: Trends in the Past Decade. Disaster Medicine and Public Health Preparedness. 2022;16(5):1725-7. doi:10.1017/dmp.2021.200.

37. de Beer P, el Harith A, van Grootheest M, Winkler A. Outbreak of kala-azar in the Sudan. Lancet. 1990;335(8683):224. doi: 10.1016/0140-6736(90)90313-t.

38. Ahmed A, Elduma A, Magboul B, Higazi T, Ali Y. The first outbreak of dengue fever in Greater Darfur, Western Sudan. Trop Med Infect Dis. 2019;4(1).

39. Botros BAM, Watts DM, Soliman AK, Salib AW, Moussa MI, Mursal H, et al. Serological evidence of dengue fever among refugees, Hargeysa, Somalia. Am J Trop Med Hyg. 1989;29(2):79-81.

40. Ahmed A, Eldigail M, Elduma A, Breima T, Dietrich I, Ali Y, et al. First report of epidemic dengue fever and malaria co-infections among internally displaced persons in humanitarian camps of North Darfur, Sudan. International Journal of Infectious Diseases. 2021;108:513-6. doi:10.1016/j.ijid.2021.05.052.

41. Bradarić N, Punda-Polić V, Milas I, Ivić I, Grgić D, Radosević N, et al. Two outbreaks of typhoid fever related to the war in Bosnia and Herzegovina. European Journal of Epidemiology. 1996;12(4):409-12.

42. Nyamusore J, Nahimana MR, Ngoc CT, Olu O, Isiaka A, Ndahindwa V, et al. Risk factors for transmission of Salmonella Typhi in Mahama refugee camp, Rwanda: a matched case-control study. Pan African Medical Journal. 2018;29:13.

43. Brown AE, Meek SR, Maneechai N, Lewis GE. Murine typhus among Khmers living at an evacuation site on the Thai-Kampuchean border. Am J Trop Med Hyg. 1988;38(1):168-71.

44. McAuley JB, Michelson MK, Hightower AW, Engeran S, Wintermeyer LA, Schantz PM. A trichinosis outbreak among Southeast Asian refugees. Am J Epidemiol. 1992;135(12):1404-10.
