# Supplementary material for: Status of zoonotic disease research in refugees, asylum seekers and internally displaced people, globally: A scoping review of forty clinically important zoonotic pathogens
Source: PLoS Negl Trop Dis. 2024 May 20;18(5):e0012164. doi: 10.1371/journal.pntd.0012164 (PMC11142688; doi:10.1371/journal.pntd.0012164)
Supplement: S11 Table — (DOCX) [file pntd.0012164.s013.docx]

**S11 Table:** Publications included in the scoping review reporting on preventative measures against infections and transmission of zoonotic pathogens

| Preventative measures | References |
| --- | --- |
| Vaccine/ prophalaxis/ MDA | [1-37] |
| Surveillance/ screening | [1, 4, 11, 16, 26, 27, 38-65] |
| Improved hygeine/ sanatation facilites | [1, 16, 21, 25, 26, 28, 30, 31, 40, 44, 47, 48, 54, 60, 66-78] |
| Animal/ vector control | [1, 4, 7, 12, 35, 38, 47, 48, 52, 61, 69, 75, 79-86] |
| Community education | [25, 30, 31, 40, 54, 60, 63, 70, 84-92] |
| Isolation/ social distancing | [60, 70, 76, 78, 87, 91-99] |
| Health care workers eduction | [38, 47, 63, 83, 87, 91, 100, 101] |
| Improved access to healthcare | [4, 38, 39, 58] |
| Nutrition programs/ vitamin supplements | [50, 80] |
| Other | [84] |

**References**

1. Berger SA, Schwartz T, Michaeli D. Infectious disease among Ethiopian immigrants in Israel. Arch Intern Med. 1989;149(1):117-9.

2. Chandrasena TGAN, Hapuarachchi HC, Dayanath MYD, Pathmeswaran A, De Silva NR. Intestinal parasites and the growth status of internally displaced children in Sri Lanka. Trop Doct. 2007;37(3):163-5.

3. Chang AH, Perry S, Du JNT, Agunbiade A, Polesky A, Parsonnet J. Decreasing intestinal parasites in recent northern California refugees. Am J Trop Med Hyg. 2013;88(1):191-7.

4. Desai AN, Ramatowski JW, Marano N, Madoff LC, Lassmann B. Infectious disease outbreaks among forcibly displaced persons: An analysis of ProMED reports 1996-2016. Conflict and Health. 2020;14(1).

5. Dorkenoo MA, Tchankoni MK, Yehadji D, Yakpa K, Tchalim M, Sossou E, et al. Monitoring migrant groups as a post-validation surveillance approach to contain the potential reemergence of lymphatic filariasis in Togo. Parasites & Vectors. 2021;14(1).

6. Enabulele EE, Platt RN, Adeyemi E, Agbosua E, Aisien MSO, Ajakaye OG, et al. Urogenital schistosomiasis in Nigeria post receipt of the largest single praziquantel donation in Africa. Acta Tropica. 2021:105916.

7. Fritzsche M, Gottstein B, Wigglesworth MC, Eckert J. Serological survey of human cysticercosis in Irianese refugee camps in Papua New Guinea. Acta Tropica. 1990;47(2):69-77.

8. Geltman PL, Cochran J, Hedgecock C. Intestinal parasites among African refugees resettled in Massachusetts and the impact of an overseas pre-departure treatment program. Am J Trop Med Hyg. 2003;69(6):657-62.

9. Gray GC, Rodier GR, Matras-Maslin VC, Honein MA, Ismail EA, Botros BA, et al. Serologic evidence of respiratory and rickettsial infections among Somali refugees. Am J Trop Med Hyg. 1995;52(4):349-53. doi: 10.4269/ajtmh.1995.52.349.

10. Hassan AO, Mero WMS. Prevalence of intestinal parasites among displaced people living in displacement camps in duhok province/Iraq. Internet Journal of Microbiology. 2020;17(1).

11. Hofstetter M, Nash TE, Cheever AW. Infection with Schistosoma mekongi in Southeast Asian refugees. J Infect Dis. 1981;144(5):420-6.

12. Jenkins-Holick DS, Kaul TL. Schistosomiasis. Urol Nurs. 2013;33(4):163-70.

13. Lin CY, Chen TC, Dai CY, Yu ML, Lu PL, Yen JH, et al. Serological investigation to identify risk factors for post-flood infectious diseases: a longitudinal survey among people displaced by Typhoon Morakot in Taiwan. BMJ Open. 2015;5(5):e007008.

14. Maaßen W, Wiemer D, Frey C, Kreuzberg C, Tannich E, Hinz R, et al. Microbiological screenings for infection control in unaccompanied minor refugees: The German Armed Forces Medical Service's experience. Military Medical Research volume. 2017;4(1).

15. Masters PJ, Lanfranco PJ, Sneath E, Wade AJ, Huffam S, Pollard J, et al. Health issues of refugees attending an infectious disease refugee health clinic in a regional Australian hospital. Australian Journal of General Practice. 2018;47(5):305-10.

16. Miller JM, Boyd HA, Ostrowski SR, Cookson ST, Parise ME, Gonzaga PS, et al. Malaria, intestinal parasites, and schistosomiasis among Barawan Somali refugees resettling to the United States: A strategy to reduce morbidity and decrease the risk of imported infections. Am J Trop Med Hyg. 2000;62(1):115-21.

17. Moaven L, Van Asten M, Crofts N, Locarnini SA. Seroepidemiology of hepatitis E in selected Australian populations. J Med Virol. 1995;45(3):326-30.

18. O'Neal SE, Robbins NM, Townes JM. Neurocysticercosis among resettled refugees from Burma. J Travel Med. 2012;19(2):118-21.

19. O'Neal SE, Townes JM, Wilkins PP, Noh JC, Lee D, Rodriguez S, et al. Seroprevalence of antibodies against Taenia solium cysticerci among refugees resettled in United States. Emerg Infect Dis. 2012;18(3):431-8.

20. Raoult D, Ndihokubwayo JB, Tissot-Dupont H, Roux V, Faugere B, Abegbinni R, et al. Outbreak of epidemic typhus associated with trench fever in Burundi. The Lancet. 1998;352(9125):353-8.

21. Ahmed A, Ali Y, Siddig EE, Hamed J, Mohamed NS, Khairy A, et al. Hepatitis E virus outbreak among Tigray war refugees from Ethiopia, Sudan. Emerg Infect Dis. 2022;28(8):1722-4. doi:10.3201/eid2808.220397.

22. Al-Hatamleh MAI, Hatmal MM, Mustafa SHF, Alzu'bi M, AlSou'b AF, Abughanam SNS, et al. Experiences and perceptions of COVID-19 infection and vaccination among Palestinian refugees in Jerash camp and Jordanian citizens: a comparative cross-sectional study by face-to-face interviews. Infectious diseases of poverty. 2022;11(1):123. doi:10.1186/s40249-022-01047-y.

23. Bojorquez-Chapela I, Strathdee SA, Garfein RS, Benson CA, Chaillon A, Ignacio C, et al. The impact of the COVID-19 pandemic among migrants in shelters in Tijuana, Baja California, Mexico. BMJ Global Health. 2022;7(3). doi:10.1136/bmjgh-2021-007202.

24. da Costa e Silva GR, Martins TLS, de Almeida Silva C, Caetano KAA, dos Santos Carneiro MA, Silva BVDE, et al. Hepatitis A and E among immigrants and refugees in Central Brazil. Revista de Saude Publica. 2022;56. doi:10.11606/S1518-8787.2022056003839.

25. da Silva HP, Abreu IN, Lima CNC, de Lima ACR, Barbosa AD, de Oliveira LR, et al. Migration in times of pandemic: SARS-CoV-2 infection among the Warao indigenous refugees in Belem, Para, Amazonia, Brazil. BMC Public Health. 2021;21(1). doi:10.1186/s12889-021-11696-7.

26. Desai AN, Mohareb AM, Elkarsany MM, Desalegn H, Madoff LC, Lassmann B. Viral hepatitis E outbreaks in refugees and internally displaced populations, sub-Saharan Africa, 2010–2020. Emerg Infect Dis. 2022;28(5):1074-6. doi:10.3201/eid2805.212546.

27. Dressler A, Finci I, Wagner-Wiening C, Eichner M, Brockmann SO. Epidemiological analysis of 3,219 COVID-19 outbreaks in the state of Baden-Wuerttemberg, Germany. Epidemiology and Infection. 2021. doi:10.1017/S0950268821000911.

28. Evbuomwan IO, Edosomwan EU, Idubor V, Bazuaye C, Abhulimhen-Iyoha BI, Adeyemi OS, et al. Survey of intestinal parasitism among schoolchildren in internally displaced persons camp, Benin City, Nigeria. Scientific African. 2022;17. doi:10.1016/j.sciaf.2022.e01373.

29. Swanson SJ, Phares CR, Mamo B, Smith KE, Cetron MS, Stauffer WM. Albendazole therapy and enteric parasites in United States-bound refugees. New England Journal of Medicine. 2012;366(16):1498-507.

30. Taylor R. Typhoid fever in the Basque Refugee Camp. British Medical Journal. 1937;1937:760-1.

31. Ul Haq KA, Gul NA, Muhammad Hammad H, Bibi Y, Bibi A, Mohsan J. Prevalence of giardia intestinalis and hymenolepis nana in afghan refugee population of mianwali district, pakistan. African Health Sciences. 2015;15(2):394-400.

32. Yauba SM, Rabasa AI, Farouk AG, Elechi HA, Ummate I, Ibrahim BA, et al. Urinary schistosomiasis in Boko Haram-related internally displaced Nigerian children. Saudi J Kidney Dis Transpl. 2018;29(6):1395-402.

33. Yildirim C, Arda B, Uz I, Uyar M, Ersel M, Yamazhan T, et al. Wars do not kill only with guns: A case of rabies in a Syrian refugee. Mediterranean Journal of Infection Microbes and Antimicrobials. 2017;6:2.

34. Zambrano LD, Samson O, Phares C, Jentes E, Weinberg M, Goers M, et al. Unresolved Splenomegaly in Recently Resettled Congolese Refugees - Multiple States, 2015-2018. MMWR. 2018;67(49):1358-62.

35. Gumisiriza N, Kugler M, Brusselaers N, Mubiru F, Anguzu R, Ningwa A, et al. Risk factors for nodding syndrome and other forms of epilepsy in northern uganda: A case-control study. Pathogens. 2021;10(11). doi:10.3390/pathogens10111451.

36. Quandelacy TM, Riefkohl A, Franco-Paredes C. Prevalence of untreated schistosomiasis among Sudanese refugees: “The Lost Boys of Sudan” in the United States. Boletin medico del Hospital Infantil de Mexico. 2010;67:503-6.

37. Samuda GM, Chan SP, Yeung CY. Vietnamese child health in a Hong Kong closed camp. Aust Paediatr J. 1988;24(2):115-7.

38. Alawieh A, Musharrafieh U, Jaber A, Berry A, Ghosn N, Bizri AR. Revisiting leishmaniasis in the time of war: the Syrian conflict and the Lebanese outbreak. International Journal of Infectious Diseases. 2014;29:115-9.

39. Bradarić N, Punda-Polić V, Milas I, Ivić I, Grgić D, Radosević N, et al. Two outbreaks of typhoid fever related to the war in Bosnia and Herzegovina. European Journal of Epidemiology. 1996;12(4):409-12.

40. Browne LB, Menkir Z, Kahi V, Maina G, Asnakew S, Tubman M, et al. Hepatitis E Outbreak Among Refugees from South Sudan - Gambella, Ethiopia, April 2014-January 2015. MMWR. 2015;64(19):537.

41. Ceccarelli G, d'Ettorre G, Riccardo F, Ceccarelli C, Chiaretti M, Picciarella A, et al. Urinary schistosomiasis in asylum seekers in Italy: an emergency currently undervalued. J Immigr Minor Health. 2013;15(4):846-50.

42. Franco-Paredes C, Dismukes R, Nicolls D, Hidron A, Workowski K, Rodriguez-Morales A, et al. Persistent and untreated tropical infectious diseases among Sudanese refugees in the United States. Am J Trop Med Hyg. 2007;77(4):633-5.

43. Garg PK, Perry S, Dorn M, Hardcastle L, Parsonnet J. Risk of intestinal helminth and protozoan infection in a refugee population. Am J Trop Med Hyg. 2005;73(2):386-91.

44. Goldenberger D, Claas GJ, Bloch-Infanger C, Breidthardt T, Suter B, Martinez M, et al. Louse-borne relapsing fever (Borrelia recurrentis) in an Eritrean refugee arriving in Switzerland, August 2015. Euro Surveill. 2015;20(32):2-5.

45. Gyorkos TW, Frappier-Davignon L, MacLean JD, Viens P. Effect of screening and treatment on imported intestinal parasite infections: Results from a randomized, controlled trial. Can J Public Health. 1989;129(4):753-61.

46. Halici-Ozturk F, Yakut K, Öcal FD, Erol A, Gökay S, Çağlar AT, et al. Seroprevalence of Toxoplasma gondii infections in Syrian pregnant refugee women in Turkey. European Journal of Obstetrics and Gynecology and Reproductive Biology. 2021;256:91-4.

47. Hoch M, Wieser A, Löscher T, Margos G, Pürner F, Zühl J, et al. Louse-borne relapsing fever (Borrelia recurrentis) diagnosed in 15 refugees from northeast Africa: Epidemiology and preventive control measures, Bavaria, Germany, July to October 2015. Euro Surveill. 2015;20(42).

48. Inci R, Ozturk P, Mulayim MK, Ozyurt K, Alatas ET, Inci MF. Effect of the Syrian Civil War on Prevalence of Cutaneous Leishmaniasis in Southeastern Anatolia, Turkey. Medical Science Monitor. 2015;21:5.

49. Jones MJ, Thompson Jr JH, Brewer NS. Infectious diseases of Indochinese refugees. Mayo Clin Proc. 1980;55(8):482-8.

50. Mitchell T, Lee D, Weinberg M, Phares C, James N, Amornpaisarnloet K, et al. Impact of Enhanced Health Interventions for United States-Bound Refugees: Evaluating Best Practices in Migration Health. Am J Trop Med Hyg. 2018;98(3):920-8.

51. Montour J, Lee D, Snider C, Jentes ES, Stauffer W. Absence of Loa loa microfilaremia among newly arrived congolese refugees in Texas. Am J Trop Med Hyg. 2017;97(6):1833-5.

52. Ntais P, Christodoulou V, Tsirigotakis N, Dokianakis E, Dedet J-P, Pratlong F, et al. Will the introduction of Leishmania tropica MON-58, in the island of Crete, lead to the settlement and spread of this rare zymodeme? Acta Tropica. 2014;132:125-30. doi:10.1016/j.actatropica.2014.01.003.

53. Parenti DM, Lucas D, Lee A, Hollenkamp RH. Health status of Ethiopian refugees in the United States. Am J Public Health. 1987;77(12):1542-3.

54. Van Enter BJD, Lau YL, Ling CL, Watthanaworawit W, Sukthana Y, Lee WC, et al. Seroprevalence of toxoplasma gondii infection in refugee and migrant pregnant women along the Thailand-myanmar border. Am J Trop Med Hyg. 2017;97(1):232-5.

55. Varkey P, Jerath AU, Bagniewski S, Lesnick T. Intestinal parasitic infection among new refugees to Minnesota, 1996-2001. Travel Med Infect Dis. 2007;5(4):223-9.

56. Wiesenthal AM, Nickels MK, Hashimoto KG. Intestinal parasites in Southeast-Asian refugees. Prevalence in a community of Laotians. JAMA. 1980;244(22):2543-4.

57. Williams B, Boullier M, Cricks Z, Ward A, Naidoo R, Williams A, et al. Screening for infection in unaccompanied asylum-seeking children and young people. Archives of Disease in Childhood. 2020;105(6):530-2.

58. Carreras-Abad C, Oliveira-Souto I, Pou-Ciruelo D, Pujol-Morro JM, Soler-Palacín P, Soriano-Arandes A, et al. Health and vaccination status of unaccompanied minors after arrival in a european border country: A cross-sectional study (2017-2020). Pediatric Infectious Disease Journal. 2022;41(11):872-7. doi:10.1097/INF.0000000000003670.

59. Chaves NJ, Gibney KB, Leder K, O'Brien DP, Marshall C, Biggs BA. Screening practices for infectious diseases among Burmese refugees in Australia. Emerg Infect Dis. 2009;15(11):1769-72. doi: 10.3201/eid1511.090777.

60. Fabris S, d'Ettorre G, Spagnolello O, Russo A, Lopalco M, D'Agostino F, et al. SARS-CoV-2 Among Migrants Recently Arrived in Europe From Low- and Middle-Income Countries: Containment Strategies and Special Features of Management in Reception Centers. Frontiers in Public Health. 2021;9. doi:10.3389/fpubh.2021.735601.

61. Hammoud S, Onchonga D, Amer F, Kocsis B. The burden of communicable diseases in Lebanon: Trends in the past decade. Disaster Medicine and Public Health Preparedness. 2022;16(5):1725-7. doi:10.1017/dmp.2021.200.

62. Kumar GS, Pezzi C, Payton C, Mamo B, Urban K, Scott K, et al. Health of ssylees compared to refugees in the United States using domestic medical examination data, 2014-2016: A cross-sectional analysis. Clinical Infectious Diseases. 2021;73(8):1492-9. doi:10.1093/cid/ciab502.

63. Rahim M, Kazi BM, Bile KM, Munir M, Khan AR. The impact of the disease early warning system in responding to natural disasters and conflict crises in Pakistan. Eastern Mediterranean Health Journal. 2010;16:S114-21. doi:10.26719/2010.16.supp.114.

64. Tambuzzi S, Cummaudo M, Maggioni L, Tritella S, Lucchesi B, Montedoro P, et al. A pilot COVID-19 surveillance program at the Zendrini center in Milan (Italy) for unaccompanied foreign minors. Children (Basel). 2022;9(10). doi:10.3390/children9101485.

65. Zwi K, Morton N, Woodland L, Mallitt K-A, Palasanthiran P. Screening and primary care access for newly arrived paediatric refugees in regional Australia: A 5 year cross-sectional analysis (2007–12). Journal of Tropical Pediatrics. 2016;63(2):109-17. doi: 10.1093/tropej/fmw059.

66. Ahmed JA, Moturi E, Spiegel P, Schilperoord M, Burton W, Kassim NH, et al. Hepatitis E outbreak, Dadaab refugee camp, Kenya, 2012. Emerg Infect Dis. 2013;19(6):1010-2.

67. Darcis G, Hayette MP, Bontems S, Sauvage AS, Meuris C, Van Esbroeck M, et al. Louse-borne relapsing fever in a refugee from Somalia arriving in Belgium. J Travel Med. 2016;23(3):3.

68. Edosomwan EU, Evbuomwan IO, Agbalalah C, Dahunsi SO, Abhulimhen-Iyoha BI. Malaria coinfection with Neglected Tropical Diseases (NTDs) in children at Internally Displaced Persons (IDP) camp in Benin City, Nigeria. Heliyon. 2020;6(8).

69. Hijawi KJF, Hijjawi NS, Ibbini JH. Detection, genotyping, and phylogenetic analysis of Leishmania isolates collected from infected Jordanian residents and Syrian refugees who suffered from cutaneous leishmaniasis. Parasitol Res. 2019;118(3):793-805.

70. Lagare A, Ibrahim A, Ousmane S, Issaka B, Zaneidou M, Kadadé G, et al. Outbreak of Hepatitis E virus infection in displaced persons camps in Diffa region, Niger, 2017. Am J Trop Med Hyg. 2018;99(4):1055-7.

71. Nyamusore J, Nahimana MR, Ngoc CT, Olu O, Isiaka A, Ndahindwa V, et al. Risk factors for transmission of *Salmonella* Typhi in Mahama refugee camp, Rwanda: a matched case-control study. Pan African Medical Journal. 2018;29:13.

72. Oboth P, Gavamukulya Y, Barugahare BJ. Prevalence and clinical outcomes of *Plasmodium falciparum* and intestinal parasitic infections among children in Kiryandongo refugee camp, mid-Western Uganda: A cross sectional study. BMC Infect Dis. 2019;19(1).

73. Osthoff M, Schibli A, Fadini D, Lardelli P, Goldenberger D. Louse-borne relapsing fever - report of four cases in Switzerland, June-December 2015. BMC Infect Dis. 2016;16(1).

74. Thomson K, Luis Dvorzak J, Lagu J, Laku R, Dineen B, Schilperoord M, et al. Investigation of hepatitis E outbreak among refugees - Upper Nile, South Sudan, 2012-2013. MMWR. 2013;62(29):581-6.

75. Wilting KR, Stienstra Y, Sinha B, Braks M, Cornish D, Grundmann H. Louse-borne relapsing fever (Borrelia recurrentis) in asylum seekers from Eritrea, the Netherlands, July 2015. Euro Surveill. 2015;20(30):2-4.

76. Altare C, Kostandova N, Okeeffe J, Omwony E, Nyakoojo R, Kasozi J, et al. COVID-19 epidemiology and changes in health service utilization in Uganda’s refugee settlements during the first year of the pandemic. BMC Public Health. 2022;22(1):1927. doi: 10.1186/s12889-022-14305-3.

77. Binga WE, Houmsou RS, Garba LC, Amuta EU, Suntaya KL. Use of rivers' water, inadequate hygiene, and sanitation as exposure of internally displaced persons (IDPs) to urogenital schistosomiasis and soil-transmitted helminthiasis in Jalingo Local Government Area (LGA), Taraba State, Nigeria. Journal of Water Sanitation and Hygiene for Development. 2022. doi:10.2166/washdev.2022.089.

78. Pham PN, Keegan K, Johnston LG, Rodas J, Restrepo MA, Wei C, et al. Assessing the impact of the COVID-19 pandemic among Venezuelan refugees and migrants in Colombia using respondent-driven sampling (RDS). BMJ Open. 2022;12(10). doi:10.1136/bmjopen-2021-054820.

79. Antinori S, Mediannikov O, Corbellino M, Raoult D. Louse-borne relapsing fever among East African refugees in Europe. Travel Med Infect Dis. 2016;14(2):110-4.

80. Brown V, Larouze B, Desve G, Rousset JJ, Thibon M, Fourrier A, et al. Clinical presentation of louse-born relapsing fever among Ethiopian refugees in northern Somalia. Ann Trop Med Parasitol. 1988;82(5):499-502.

81. Dunya G, Habib R, Moukarbel RV, Khalifeh I. Head and neck cutaneous leishmania: clinical characteristics, microscopic features and molecular analysis in a cohort of 168 cases. Eur Arch Otorhinolaryngol. 2016;273(11):3819-26.

82. Gurses G, Ozaslan M, Zeyrek FY, Kilic IH, Doni NY, Karagoz ID, et al. Molecular identification of Leishmania spp. isolates causes cutaneous leishmaniasis (CL) in Sanliurfa Province, Turkey, where CL is highly endemic. Folia Microbiol (Praha). 63(3):353-9.

83. Hytönen J, Khawaja T, Grönroos JO, Jalava A, Meri S, Oksi J. Louse-borne relapsing fever in Finland in two asylum seekers from Somalia. APMIS. 2017;125(1):59-62.

84. Kanani K, Amr ZS, Shadfan B, Khorma R, Rø G, Abid M, et al. Cutaneous leishmaniasis among Syrian refugees in Jordan. Acta Tropica. 2019;194:169-71.

85. Ahmed A, Eldigail M, Elduma A, Breima T, Dietrich I, Ali Y, et al. First report of epidemic dengue fever and malaria co-infections among internally displaced persons in humanitarian camps of North Darfur, Sudan. International Journal of Infectious Diseases. 2021;108:513-6. doi:10.1016/j.ijid.2021.05.052.

86. Nyakarahuka L, Whitmer S, Kyondo J, Mulei S, Cossaboom CM, Telford CT, et al. Crimean-Congo hemorrhagic fever outbreak in refugee settlement during COVID-19 pandemic, Uganda, April 2021. Emerg Infect Dis. 2022;28(11):2326-9. doi:10.3201/eid2811.220365.

87. Ly TDA, Nguyen NN, Hoang VT, Goumballa N, Louni M, Canard N, et al. Screening of SARS-CoV-2 among homeless people, asylum-seekers and other people living in precarious conditions in Marseille, France, March–April 2020. International Journal of Infectious Diseases. 2021;105:1-6.

88. Stehr-Green JK, Schantz PM. Trichinosis in Southeast Asian refugees in the United States. Am J Public Health. 1986;76(10):1238-9.

89. Sulaiman AA, Elmadhoun WM, Noor SK, Bushara SO, Almobarak AO, Awadalla H, et al. An outbreak of cutaneous leishmaniasis among a displaced population in North Sudan: Review of cases. J Family Med Prim Care. 2019;8(2):556-63.

90. Tittle BS, Harris JA, Chase PA. Health screening of Indochinese refugee children. Am J Dis Child. 1982;136(8):697-700.

91. Knust B, Wongjindanon N, Moe AA, Herath L, Kaloy W, Soe TT, et al. Enhancing respiratory disease surveillance to detect COVID-19 in shelters for displaced persons, Thailand-Myanmar Border, 2020-2021. Emerg Infect Dis. 2022;28(13):S17-s25. doi:10.3201/eid2813.220324.

92. Mellou K, Gkolfinopoulou K, Andreopoulou A, Tsekou A, Papadima K, Stamoulis K, et al. A COVID-19 outbreak among migrants in a hosting facility in Greece, April 2020. Journal of Infection Prevention. 2022;23(5):235-8. doi:10.1177/17571774221092568.

93. Bloch-Infanger C, Bättig V, Kremo J, Widmer AF, Egli A, Bingisser R, et al. Increasing prevalence of infectious diseases in asylum seekers at a tertiary care hospital in Switzerland. PLoS One. 2017;12(6):e0179537.

94. Le Bihan C, Faucherre V, Le Moing V, Mehenni A, Nantes D, Da Silva A, et al. COVID-19: The forgotten cases of hidden exiles. Infect Dis Now. 2021.

95. Altare C, Kostandova N, Okeeffe J, Hayek H, Fawad M, Musa Khalifa A, et al. COVID-19 epidemiology and changes in health service utilization in Azraq and Zaatari refugee camps in Jordan: A retrospective cohort study. PLoS Medicine. 2022;19(5). doi:10.1371/journal.pmed.1003993.

96. Kondilis E, Papamichail D, McCann S, Carruthers E, Veizis A, Orcutt M, et al. The impact of the COVID-19 pandemic on refugees and asylum seekers in Greece: A retrospective analysis of national surveillance data from 2020. EClinicalMedicine. 2021;37. doi:10.1016/j.eclinm.2021.100958.

97. Sisti LG, Di Napoli A, Petrelli A, Rossi A, Diodati A, Menghini M, et al. Covid-19 impact in the italian reception system for migrants during the nationwide lockdown: A national observational study. International Journal of Environmental Research and Public Health. 2021;18(23). doi:10.3390/ijerph182312380.

98. Turunen T, Kontunen K, Sugulle K, Hieta P, Snellman O, Hussein I, et al. COVID-19 outbreak at a reception centre for asylum seekers in Espoo, Finland. 2021;3:100043.

99. Zöllkau J, Ankert J, Pletz MW, Mishra S, Seliger G, Lobmaier SM, et al. Hepatitis E, Schistosomiasis and Echinococcosis–Prevalence in a cohort of pregnant migrants in Germany and their influence on fetal growth restriction. Pathogens. 2022;11(1). doi:10.3390/pathogens11010058.

100. Paran Y, Ben-Ami R, Orlev B, Halutz O, Elalouf O, Wasserman A, et al. Chronic schistosomiasis in African immigrants in Israel: Lessons for the non-endemic setting. Medicine (Baltimore). 2019;98(52).

101. Schroeder Jr HW, Yarrish RL, Perkins TF, Lee C. Sequential disseminated tuberculosis and toxoplasmosis in a Haitian refugee. Southern Medical Journal. 1984;77(4):533-4.
