# Supplementary material for: Status of zoonotic disease research in refugees, asylum seekers and internally displaced people, globally: A scoping review of forty clinically important zoonotic pathogens
Source: PLoS Negl Trop Dis. 2024 May 20;18(5):e0012164. doi: 10.1371/journal.pntd.0012164 (PMC11142688; doi:10.1371/journal.pntd.0012164)
Supplement: Attachment — Submitted filename: Response to reviewers comments.docx [file pntd.0012164.s015.docx]

We are grateful to the reviewers for taking time to review our manuscript and providing constructive feedback. Please find our point-by-point responses below. Line numbers refer to the track changed version.

**Methods**

**Reviewer #1:** Line 127: the reference is missing, then it is not clear why the work is only about 40 zoonotic pathogens.

Thank you, this was meant to refer to Table 1. Unfortunately, the automated cross-reference feature in word created an error in the submitted version. This has now been corrected in lines 164 and 173. Further details regarding limiting the review to 40 pathogens are provided in lines 137-147.

Line 135: reference missing, comment as above

Please see our response above.

Table 1 (page 7) maybe presenting pathogens in alphabetic order within pathogen groups (bacteria, viruses, parasites) could be appropriate (in this table it does not seem to be about relevance, occurrence)

Thank you, the table has been modified as suggested and included at line 188.

**Reviewer #2:** Methodology is sound.

Thank you.

**Results**

**Reviewer #1**: Figure 1: maybe this will feet better in the method section

We included Figure 1 in the results section to keep in line with the PRISMA-ScR guidelines, provided in supplementary text 1.

Line 208: check for spelling (retrieved)

The spelling has been corrected at line 249.

Line 270: it is not clear why sample collection as in the introduction (line 128) it is said that “Only publication with a clinical or laboratory diagnosis of one of the pathogens were included”. That means that for some there were no lab diagnosis. Please check and

We excluded publications were self-reported cases were described. For example, we retrieved a number of publications on mental health during the COVID-19 pandemic where participants reported if they experienced COVID-19, but details about the diagnosis were not provided in the publication. We have clarified this at line 165-166.

Line 270-276: maybe this will fit better in the method section

This is the legend for Figure 5, however, we have now also provided these details in the methods section at line 209-211.

Line 289-294: this can be deleted as the entire list of the 40 pathogens of interest is provided (cfr lines 310-311)
Line 300-304: same as above
These sections have been removed.

Lines 330-342: it is not clear what exactly was/were “suggestive potential predictors of infection”

We have rephrased this sentence to clarify the meaning of presumptive predictors at line 390-391.

Figure 8: spelling (Hygiene)

The figure has been corrected at line 405.

Figure 8 is reported two times, please check

The second figure has been removed.

Line 370: spelling

Several authors reviewed the sentence at line 370; however, the spelling appears to be correct.

“Animal and vector control programs were included in 20/99 (20.2%) publications while healthcare worker education and improved access to healthcare were discussed in 8/99 (8.1%) and 5/99 (5.1%) publications, respectively.”

Figure 9: please provide spelling for acronym
The spelling for MDA has been added at line 425.

**Discussion**

Line 380-381: there are gap regarding impact but also regarding description, maybe this aspect could be added

Thank you, this sentence has been revised as suggested at line 431.

Line 406: please provide spelling for IFAs (Immunofluorescence Agglutination, Immunofluorescence Assay, Indirect Fluorescent Antibody test…)
Spelling for IFA had been included at line 456-457.

Line 423: please confirm it is “OF” or should be “OR”
Thank you, this has been corrected to “OR” at line 474.

Line 436:please rephrase

This sentence has been rephrased at line 488-491.

Line 440: Latin America instead of Africa
This has been corrected to Latin America at line 495.

Line 465 & 478 (also line 338): As most of the original works were cross-sectional studies, then it is more about “associated factors as you mentioned on lines 319 320) regarding outbreaks in the results section. Please make appoint on the that in the discussion and rephrase where appropriate (risk, predictors).

Thank you, we have rephrased the section on outbreaks in the discussion at line 520 and 524.

**Limitations**

Lines 532-534: Not including publication might also had influence on the number of publications (from Africa)
Thank you for this comment, we have elaborated on this point at line 596-597.

**Reviewer #2**: Results are fine. However, I found two Figure 8 in the manuscript, so the authors have to revise the manuscript accordingly.

The second figure has been removed.

**Conclusions**

**Reviewer #1:** Line 557 : spelling

The spelling has been corrected at line 620.

**Reviewer #2:** Conclusions are supported by the data.
Thank you.

**Editorial and Data Presentation Modifications**

**Reviewer #1**: Abstract

Line 36: For a reader relying only to the abstract, it may appear that there are only 40 clinically zoonotic pathogens of interest (in general), please rephrase to highlight that your work is about chosen 40 pathogens.

We have rephrased the abstract to highlight that we selected 40 pathogens to include in this review, at line 39.

Line 46 : maybe it could be better to add “dates” of publications ranged from 1937 to 2022 (need to check in the text it said date of sample collection; please confirm.

We have added “dates of” to this sentence at line 44. Date of sample collection was used only for analysis of migration routes, this has been now been explained in the methods section at lines 203-205.

**Introduction**

Line 87: please delete “are” after LMICs

This has been deleted at line 113.

Line 89-90: It is no clear whether 20% represent illnesses & mortality

The 20% represent both illnesses and mortality, this has been rephrased to “disease burden” at line 115.

**Reviewer #2**: I suggest that the manuscript undergo Major Revision before it could be considered again for publication.

--------------------

**Summary and General Comments**

**Reviewer #1:** Thank you for the opportunity to review this manuscript.

The manuscript by Oakley and colleagues touches an important issue: zoonotic pathogens in a forcibly displaced people (refugees, asylum seekers and internally displaced people).
As the authors show in their manuscript, only few data exist regarding zoonotic infection in this “special population” of displaced people and for the few that exist there is a gap in the spectrum of pathogens studied.
One of the strength is to bring attention to zoonotic infection in displaced people, knowing that more than 60% of emerging and re-emerging infections are zoonotic and that forcibly displaced people are often in crowded settings with poor hygiene and sanitation and low access to health services . These results highlight the fact that there is still need to describe the burden and the impact of zoonotic diseases in displaced population for a better planning of interventions.
However, in the manuscript, the authors fail to be more clear in what was the rationale for choosing only 40 zoonotic pathogens. The author mentioned tuberculosis among the most reported infection in human (line 210) not included in this review. One could hope to see data on zoonotic tuberculosis (tuberculosis/bovine tuberculosis) and other zoonotic infections; or a clear explanation why the restricted 40 pathogens only.

We would like to thank the reviewer for their comments. The selection of 40 pathogens was due feasibility of developing a database search string that accurately included all search terms for all zoonotic pathogens and their diseases. This has now been explained in more detail in the introduction at line 137-147, and further in the limitations section at line 579-588.

**Reviewer #2**: It will be great if the authors elaborate on the differences between refugees, asylum seekers and internally displaced people, since these respective cohorts are central to the story of the manuscript. I particularly enjoyed reading the authors describing an example of the Rohingya refugees migrating to Bangladesh with their livestock to show the risk of disease transmission (Lines 504-506). I believe the same should be described for refugees, asylum seekers and internally displaced people, so that the manuscript is further strengthened.

We have now provided definitions in the introduction to describe the three populations included in the review at lines 85-109.

Does the study require registration with the IRB? I suggest the manuscript to be sent for language editing.
The study does not require registration with the IRB.

Several revisions to be made, see below;
Table 1 - replace MERS virus with MERS CoV, replace SARS virus with SARS CoV and Covid with coronavirus disease (COVID-19)

Thank you, these terms have been updated throughout the manuscript, figures and supplementary tables.

Line 190 - Hanta virus or hantavirus?

Hantavirus is the correct term, <https://www.cdc.gov/hantavirus/index.html>

Line 196 - severe acute respiratory syndrome coronavirus (SARS CoV). Please standardize throughout.

Thank you, this term has been updated through the manuscript, figures and supplementary tables.

Line 197 - Middle East Respiratory Syndrome coronavirus (MERS CoV). Please standardize throughout.

Thank you, this term has been updated through the manuscript, figures and supplementary tables.

Line 284-286 - Geographical terms eg. Northern/Western/Eastern should probably be checked for accuracy. The commonly accepted terms are North Africa/West Africa/Southeast Asia etc.

The geographical terms used in our review are in line with the United Nations Statistics Divisions geographical regions (<https://unstats.un.org/unsd/methodology/m49/>) which were used for the analysis.

Line 292 & 302 – chikungunya

These sections were deleted as per the recommendation of reviewer 1.

Line 293 & 303 - West Nile virus

Thank you, this term has been updated through the manuscript, figures and supplementary tables.

Fig 8 - There are two Fig 8 in the document.
The second figure has been removed.
