# Supplementary material for: Status of zoonotic disease research in refugees, asylum seekers and internally displaced people, globally: A scoping review of forty clinically important zoonotic pathogens
Source: PLoS Negl Trop Dis. 2024 May 20;18(5):e0012164. doi: 10.1371/journal.pntd.0012164 (PMC11142688; doi:10.1371/journal.pntd.0012164)
Supplement: Attachment — Submitted filename: ScR_Response to reviewers comments 2.docx [file pntd.0012164.s016.docx]

Reviewer's Responses to Questions

Key Review Criteria Required for Acceptance?
As you describe the new analyses required for acceptance, please consider the following:

Methods
-Are the objectives of the study clearly articulated with a clear testable hypothesis stated?
-Is the study design appropriate to address the stated objectives?
-Is the population clearly described and appropriate for the hypothesis being tested?
-Is the sample size sufficient to ensure adequate power to address the hypothesis being tested?
-Were correct statistical analysis used to support conclusions?
-Are there concerns about ethical or regulatory requirements being met?

Reviewer #2: OK
--------------------

Results
-Does the analysis presented match the analysis plan?
-Are the results clearly and completely presented?
-Are the figures (Tables, Images) of sufficient quality for clarity?

Reviewer #2: OK
--------------------

Conclusions
-Are the conclusions supported by the data presented?
-Are the limitations of analysis clearly described?
-Do the authors discuss how these data can be helpful to advance our understanding of the topic under study?
-Is public health relevance addressed?

Reviewer #2: OK
--------------------

Editorial and Data Presentation Modifications?
Use this section for editorial suggestions as well as relatively minor modifications of existing data that would enhance clarity. If the only modifications needed are minor and/or editorial, you may wish to recommend “Minor Revision” or “Accept”.

Reviewer #2: (No Response)

--------------------

Summary and General Comments
Use this section to provide overall comments, discuss strengths/weaknesses of the study, novelty, significance, general execution and scholarship. You may also include additional comments for the author, including concerns about dual publication, research ethics, or publication ethics. If requesting major revision, please articulate the new experiments that are needed.

Reviewer #2: The authors have revised the manuscript according to my earlier comments, however I still find that the manuscript could be further improved. Please refer to my comments below;

Line 414-hantavirus (please revise)
Corrected at line 227 and 425 (track changed version).

Lines 462-466-The feasibility of conducting research in IDP and AS populations can also be affected by these challenging conditions and may explain the disparity seen with research in refugees, some of who will have reached more stable settings and undergone pre-departure or post-arrival screening

(Can the authors give an example of any country that has such health screening initiative for refugees? Perhaps even discuss the role of UNHCR?)

Thank you for your comment, further discussion has been provided at lines 477 – 487 (track changed version).

Figure 5-The descriptions of the respective continents/regions have to be corrected. Eg. North America, Southeast Asia, West Africa, Central Africa; not Northern America, Southeastern Asia, Western Africa, Middle Africa. It is not just limited to the above mentioned regions, so please revise them accordingly. Also, please describe New Zealand separately.

The geographical terms used in our review are in line with the United Nations Statistics Divisions Geoscheme, which was used for the analysis, described at line 202 – 207 (track changed version).

These geological regions while based on continental regions (e.g. North America) have been “…further subdivided into sub-regions and intermediary regions drawn as to obtain greater homogeneity in sizes of population, demographic circumstances and accuracy of demographic statistics.” (<https://unstats.un.org/unsd/methodology/m49/>). The sub-regions were used for the analysis in our review rather than the larger continental regions as it was expected that there may be differences in disease profiles and migration patterns between sub-regions belonging to the same continental region, reflecting differences in climate and environment as well as socio-economic determinants of the populations.

For example:

“Q: What's the difference between North America and Northern America in M49?

A: North America (numerical code 003) contains Northern America (numerical code 021), the Caribbean (numerical code 029) and Central America (numerical code 013).” (<https://unstats.un.org/unsd/methodology/m49/#fn2>)

The UN geographical region termed “Australia and New Zealand” includes Australia, Christmas Island, Cocos (Keeling) Islands, Heard Island and McDonald Islands, New Zealand and Norfolk Island.


 
